# Palladium(0)-Catalysed Stereoselective Synthesis of Unsaturated Aryl β-*O*-Glycosides and Oligosaccharides: Influence of Protecting Groups and Reaction Conditions

**DOI:** 10.3390/molecules31172965

**Published:** 2026-08-25

**Authors:** Aloïs Chenet, Robert Kołodziuk, Anna Zawisza

**Affiliations:** 1 Ecole Superieure de Chimie Organique et Minerale (ESCOM), Allée du Réseau Jean Marie Buckmaster 1, 60200 Compiegne, France; alois.chenet@orange.fr; 2 Department of Organic and Applied Chemistry, Faculty of Chemistry, University of Lodz, Tamka 12, 91-403 Lodz, Poland

**Keywords:** d-glucal, *O*-glycosides, oligosaccharides, Ferrier rearrangement, allylic alkylation reaction, homogeneous catalysis, palladium

## Abstract

The Pd(0)-catalysed aryloxylation of 6-*O*-*tert*-butyldimethylsilyl-3,4-di-*O*-isobutyloxycarbonyl-d-glucal (**3**) with a series of phenolic nucleophiles was investigated as an efficient approach to the synthesis of unsaturated aryl *O*-glycosides. Under the optimized reaction conditions, the corresponding 2,3- and 3,4-unsaturated *O*-glycosides were obtained in good to excellent yields and with high β-selectivity. In most cases, the reactions proceeded with a marked preference for the formation of 2,3-unsaturated β-glycosides, whereas chlorophenols exhibited distinct reactivity patterns, resulting in diminished regioselectivity and the additional formation of α-anomeric 2,3-unsaturated glycosides. Comparison with the previously reported reactions of 6-*O*-*tert*-butyldiphenylsilyl-3,4-di-*O*-isobutyloxycarbonyl-d-glucal demonstrated a pronounced influence of the silyl protecting group on the product yields and regioselectivity, with the TBDMS-protected donor generally affording higher yields and enhanced selectivity toward 2,3-unsaturated products. Furthermore, the developed methodology was successfully extended to carbohydrate nucleophiles, enabling the synthesis of model trisaccharides and demonstrating its applicability to oligosaccharide synthesis.

## 1. Introduction

Carbohydrates are fundamental organic compounds that play vital roles in living organisms, serving as energy sources, structural components, and carriers of biological information. Among them, glycosides are of particular interest because of their remarkable structural diversity and broad spectrum of biological activities. These features make glycosides attractive targets in organic synthesis, medicinal chemistry, and pharmaceutical research [1,2,3,4,5,6].

Unsaturated glycosides, especially 2,3- and 3,4-unsaturated *O*-glycosides, have long attracted considerable attention due to their enhanced chemical reactivity and their utility as versatile intermediates in the synthesis of oligosaccharides, glycoconjugates, and other biologically active molecules. In particular, β-*O*-glycosides are of special importance, as their stereochemistry closely resembles that of naturally occurring glycosidic linkages, which strongly influences both their chemical behavior and biological recognition [7,8].

Traditional methods for the synthesis of unsaturated *O*-glycosides, such as Ferrier rearrangements [9,10,11,12,13] and elimination–reduction strategies [14,15,16,17,18,19,20,21,22,23,24,25,26,27,28], require careful control of reaction conditions to achieve high levels of regio- and stereoselectivity. By the late twentieth century, transition-metal catalysis, particularly that involving Pd(0) complexes, had also become a powerful tool in the synthesis of unsaturated glycosides. Pd(0)-catalysed reactions exploit the unique reactivity of unsaturated substrates, enabling transformations to proceed under mild conditions. Moreover, the appropriate choice of ligands, solvents, and reaction temperature allows precise control over selectivity, while the broad compatibility of Pd(0) catalysis with various functional groups facilitates the synthesis of complex molecules without the need for extensive protecting-group manipulations and subsequent deprotection steps in carbohydrate substrates [29,30,31,32,33,34,35].

Protecting groups play a crucial role in determining both the reactivity and selectivity of glycosylation reactions. Strategic protection of hydroxyl functionalities directs the formation of specific glycoside isomers, minimizes side reactions, and improves overall yields. Therefore, optimization of reaction parameters is essential to fully exploit Pd(0)-catalysed methodologies for the synthesis of 2,3- and 3,4-unsaturated β-*O*-glycosides.

**Scheme 1:**
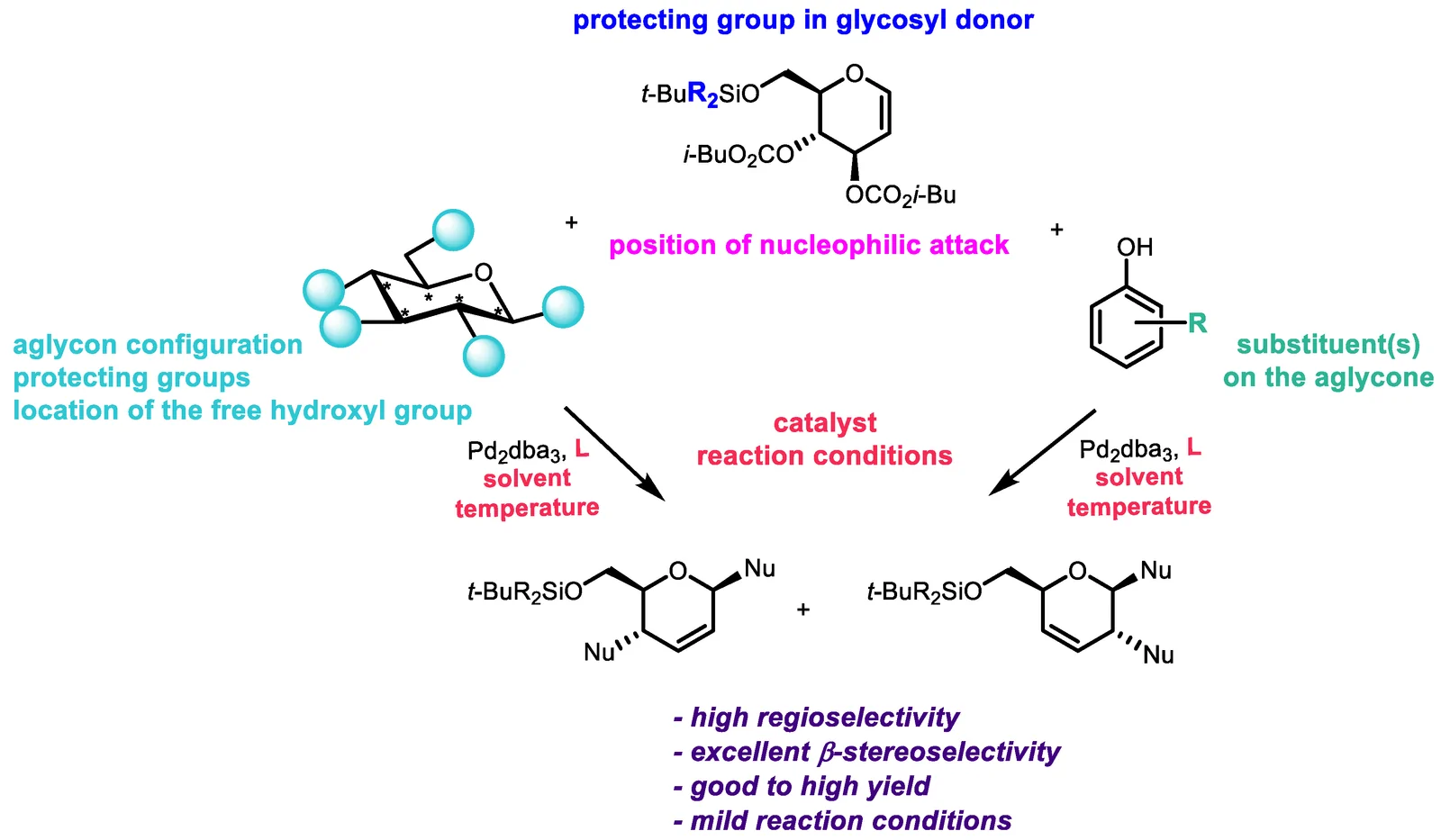
Proposed synthetic strategy for unsaturated aryl β-*O*-glycosides and oligosaccharides—key synthetic challenges.

In the present study, we report a systematic investigation of the Pd(0)-catalysed synthesis of otherwise difficult-to-access 2,3- and 3,4-unsaturated aryl β-*O*-glycosides via Tsuji–Trost-type glycosylation, together with the extension of the developed methodology to oligosaccharide synthesis. In contrast to the widely used Ferrier rearrangement, which typically provides such unsaturated glycosides with an α-configuration, the present approach enables access to the β-configured products. The key synthetic challenges associated with the preparation of these compounds are summarized in Scheme 1. Particular emphasis is placed on elucidating the influence of protecting groups in the glycosyl donor, substituents in the aglycone, and reaction conditions on reactivity and stereoselectivity. These studies establish a versatile platform for the stereoselective synthesis of structurally well-defined unsaturated glycosides with potential applications in medicinal chemistry and glycobiology.

## 2. Results and Discussion

In continuation of our studies on Pd(0)-catalysed carbon–oxygen bond formation [36], and with particular emphasis on applying this mild approach in carbohydrate chemistry, we decided to investigate the use of 6-*O*-*tert*-butyldimethylsilyl-3,4-di-*O*-isobutyloxycarbonyl-d-glucal for the synthesis of 2,3- and 3,4-unsaturated *O*-glycosides.

**Scheme 2:**
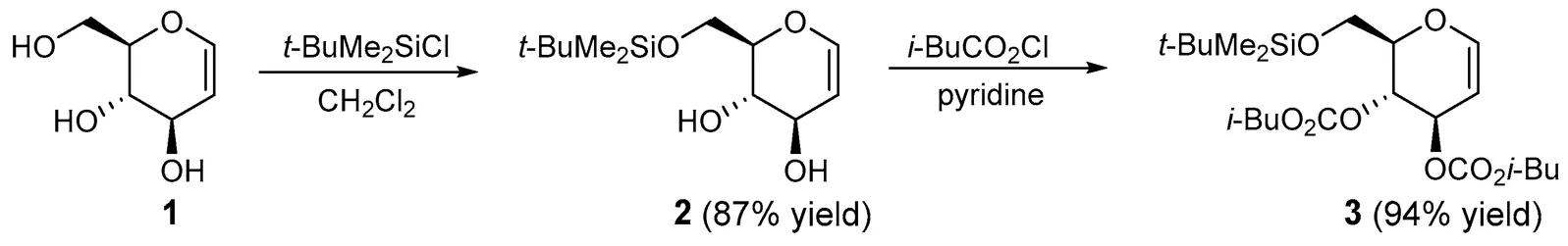
Synthesis of unsaturated carbohydrate **3**.

We anticipated that phenols could react with a π-allyl palladium intermediate, generated from a Pd(0) complex and an unsaturated carbohydrate bearing a suitable leaving group at the allylic position, leading to the formation of unsaturated *O*-glycosides. For this purpose, we selected the 1,2-unsaturated carbohydrate **3**, which can be readily obtained from d-glucal (**1**) as outlined in Scheme 2.

Commercially available d-glucal (**1**) was initially treated with *tert*-butyldimethylsilyl chloride in dichloromethane, affording the monosilylated derivative **2** in 87% yield. Subsequently, compound **2** was reacted with isobutyl chloroformate at 0 °C to produce the corresponding dicarbonate **3** in 94% yield.

**Scheme 3:**
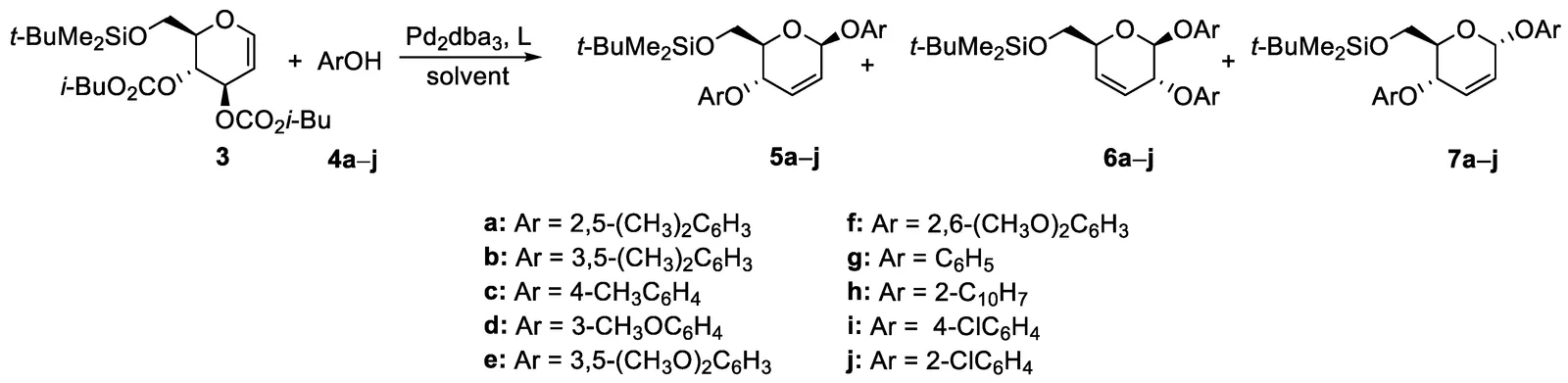
Synthesis of 2,3-and 3,4-unsaturated *O*-glycosides.

The optimization of the *O*-glycosylation reaction was carried out using dicarbonate **3** as the glycosyl donor and 2,5-dimethylphenol **4a** as the acceptor (Scheme 3, Table 1).

Initial experiments performed in tetrahydrofuran at 20 °C with dppb as the ligand resulted in a strong preference for the formation of 2,3-unsaturated *O*-glycoside **5a** over its 3,4-unsaturated isomer **6a** (94:6), although the overall yield remained low (Table 1, entry 1).

Increasing the reaction temperature to 60 °C led to a significant improvement in conversion, affording a combined yield of 79% with a **5a**:**6a** ratio of 86:14 (Table 1, entry 2). A similar selectivity was observed when dppe was used as the ligand, providing **5a** and **6a** in a ratio of 87:13 and a total yield of 74% (Table 1, entry 3). In contrast, the use of triphenylphosphine resulted in a notable decrease in selectivity, giving a nearly equimolar mixture of both isomers (56:44) and a lower overall yield (39%, Table 1, entry 4).

The effect of the solvent was also investigated (Table 1, entries 5–7). When acetonitrile was employed, the reaction proceeded with good selectivity toward **5a** (84:16), but with a moderate total yield of 43% (Table 1, entry 5). The use of dichloromethane improved the overall yield to 59%, although with slightly reduced selectivity (72:28, Table 1, entry 6). In toluene, comparable results were obtained, affording a 70:30 ratio of **5a** to **6a** and a total yield of 52% (Table 1, entry 7).

The structures of the isomeric products **5a** and **6a** were determined based on spectral analysis, including ^1^H-NMR, ^13^C-NMR, ^1^H-^1^H COSY, and ^1^H-^13^C HMQC techniques. Characteristic differences in the chemical shifts of the protons and carbons were identified for the 1,4- and 1,2-disubstituted derivatives, specifically for H-4 and C-4 in **5a** (4.84 ppm and 67.9 ppm, respectively) and H-2 and C-2 in **6a** (4.93 ppm and 72.9 ppm, respectively). Furthermore, a downfield shift for carbon C-1 was recorded at 99.3 ppm for compound **6a**, compared to 93.8 ppm for **5a**, suggesting the presence of a 3,4-unsaturated bond in the sugar ring (Table 2, entry 1) [37]. The presence of duplicate signals for protons and carbon atoms originating from the nucleophile indicates two non-equivalent *O*-phenyl substituents in both isomers. Importantly, the reaction proceeds in a fully stereospecific manner, leading exclusively to the formation of β-anomers. This is confirmed by the proton H-1 signal for the 2,3-unsaturated isomer **5a**, which is observed at 5.79 ppm. According to literature data, the signal for the α-anomer would appear at lower values—around 5.69 ppm [38]. Notably, obtaining a β-anomer via Ferrier rearrangement is particularly challenging due to the anomeric effect. Detailed signal assignments and stereochemical configurations of products **5a** and **6a** are provided in the Supporting Information.

Aryloxylation of unsaturated carbohydrate **3** was subsequently investigated with a series of substituted phenols 4**a**–**j** under the optimized Pd(0)-catalysed conditions in THF at 60 °C using dppb as the ligand. In most cases, the reactions proceeded with high regioselectivity toward the formation of the 2,3-unsaturated *O*-glycosides **5** (Table 3). The reaction of carbohydrate **3** with 3,5-dimethylphenol (**4b**) afforded predominantly the 2,3-unsaturated *O*-glycoside **5b**, giving a **5**:**6** ratio of 91:9 and a total isolated yield of 75% (Table 3, entry 2). Similarly, coupling with 4-methylphenol (**4c**) provided the corresponding unsaturated glycosides with a **5**:**6** ratio of 90:10 and an excellent total yield of 87% (Table 3, entry 3). When 3-methoxyphenol (**4d**) was employed as the nucleophile, the reaction remained highly selective toward the 2,3-unsaturated isomer **5d**, affording the products in a **5**:**6** ratio of 88:12 and 64% isolated yield (Table 3, entry 4). In the case of 3,5-dimethoxyphenol (**4e**), the desired glycosides were obtained in 78% yield with a strong preference for isomer **5** (**5**:**6** = 85:15, Table 3, entry 5). A markedly different result was observed for 2,6-dimethoxyphenol (**4f**). The reaction exhibited very low conversion and furnished exclusively the 2,3-unsaturated glycoside **5f** in only 5% yield (Table 3, entry 6). The diminished reactivity is most likely associated with the increased steric hindrance generated by the two ortho methoxy substituents. Unsubstituted phenol (**4g**) reacted smoothly under the applied conditions, yielding the corresponding *O*-glycosides in 80% total yield with a **5**:**6** ratio of 87:13 (Table 3, entry 7).

Similarly, naphth-2-ol (**4h**) showed comparable reactivity and regioselectivity, affording predominantly the 2,3-unsaturated *O*-glycoside **5h** with a **5**:**6** ratio of 84:16 and a total isolated yield of 49% (Table 3, entry 8). A different behavior was observed for the chlorophenols. The reaction with 4-chlorophenol (**4i**) afforded three products in a ratio of 59:30:11. The corresponding *O*-glycosides **5i** and **6i** were formed in a **5**:**6** ratio of 59:30, while the third product was identified as 2,3-dideoxy-α-d-*erythro*-hex-2-enopyranoside **7i**. The combined isolated yield of all products reached 90% (Table 3, entry 9) (detailed spectroscopic data for products **5i**, **6i**, and **7i** are provided in the Supporting Information). In contrast, coupling of glycal **3** with 2-chlorophenol (**4j**) resulted in a marked preference for the 3,4-unsaturated isomer **6j**. The products were obtained in a **5**:**6**:**7** ratio of 11:78:11 with a total isolated yield of 74% (Table 3, entry 10). In this case, the additional product was likewise identified as 2,3-dideoxy-α-d-erythro-hex-2-enopyranoside **7j**.

**Scheme 4:**
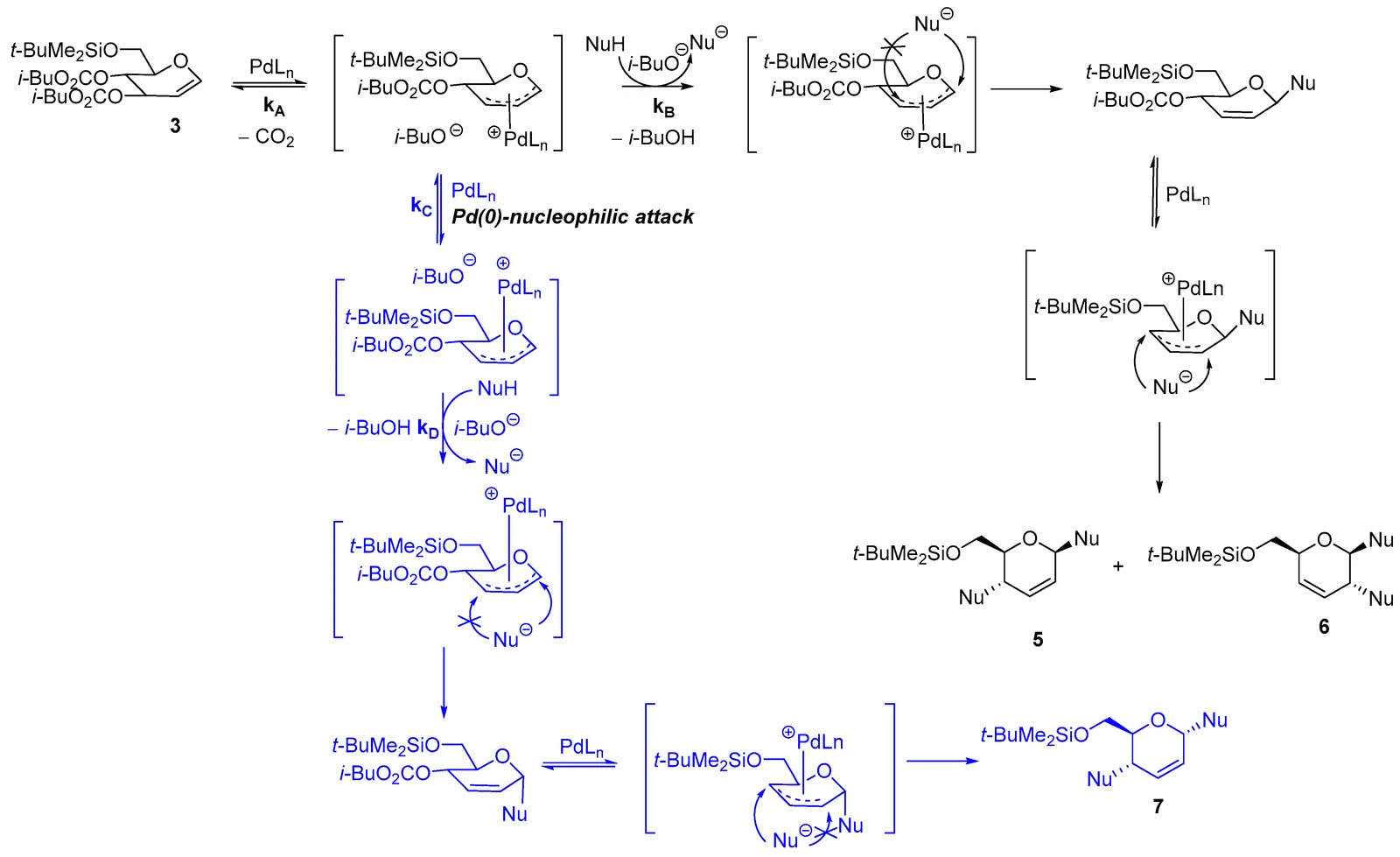
Mechanism of the Pd(0)-catalysed aryloxylation of unsaturated carbohydrate **3** with phenols.

The formation of glycosides **5** and **6** can be explained by the initially generated η^2^-π-alkene-palladium complex, followed by oxidative addition to give the corresponding η^3^-π-allyl intermediate (Scheme 4) [39]. Subsequent nucleophilic attack at C-1 leads predominantly to β-glycosidic products, while further rearrangement of the double bond enables nucleophilic attack at either C-2 or C-4, resulting in the formation of the 2,3-unsaturated glycosides **5** and the 3,4-unsaturated isomers **6**. In the case of less reactive nucleophiles, such as chlorophenols, the reduced rate of nucleophilic attack increases the lifetime of the allylic palladium intermediate and facilitates competitive isomerization during the initial stage of the catalytic cycle. This process may additionally lead to the formation of the 2,3-unsaturated α-glycoside 7.

The isomerization process, which formally corresponds to nucleophilic attack by Pd(0) on the allylic intermediate, was described by Bäckvall and Granberg [40,41]. When the oxidative addition step is rate-determining (k_A_ ≪ k_B_ ≈ k_D_), a relatively high concentration of the Pd(0)-phosphine complex remains in the reaction mixture because it is consumed more slowly than it is generated (Scheme 4). If Pd(0) attack on the intermediate occurs more rapidly than nucleophilic attack (k_C_ ≫ k_B_ ≈ k_D_), isomerization takes place. In the case of highly reactive nucleophiles (k_C_ ≪ k_B_ ≈ k_D_), isomerization is not observed.

When the nucleophile is less reactive, sterically demanding, or the approach required for nucleophilic attack is hindered, the nucleophilic attack may become the rate-limiting step (k_A_ ≫ k_B_ ≈ k_D_). Under these conditions, the lifetime of the (η^3^-allyl)palladium intermediate increases, thereby enhancing the probability of Pd(0) attack and consequently promoting isomerization of the intermediate.

The comparison of the 6-*O*-TBDMS- and 6-*O*-TBDPS-protected glucals reveals that the silyl protecting group exerts a substantial influence on the selectivity of the Pd(0)-catalysed aryloxylation. Since both the TBDMS- and TBDPS-protected donors afforded β-glycosides predominantly under the optimized conditions, the principal difference between the two systems concerns the regioselectivity, namely the distribution between the 2,3-unsaturated product **5** and the 3,4-unsaturated product **6**. For the TBDMS derivative, the 2,3-unsaturated products were generally favored, whereas the TBDPS analogue often showed lower regioselectivity and, in the case of **4c** the product ratio was reversed. Thus, changing the protecting group from TBDMS to the significantly bulkier TBDPS group can alter the relative accessibility of the two reaction pathways from the π-allyl palladium intermediate. Rather than acting solely through a simple steric blocking effect, the silyl group may modify the conformational preferences of the carbohydrate-derived π-allyl palladium complex and the spatial relationship between the metal centre, the allylic framework, and the nucleophile. This may change the relative barriers to nucleophilic attack at the two reactive positions and consequently shift the **5**:**6** ratio.

The available data do not support the conclusion that a particular position of the substituent on the phenolic nucleophile is generally required to obtain high regioselectivity with either a TBDMS- or TBDPS-protected donor. For example, the TBDMS system gives high selectivity with 2,5-dimethylphenol (**4a**; **5**:**6** = 86:14) and 3,5-dimethylphenol (**4b**; 91:9), while 4-methylphenol (**4c**) gives a ratio of 90:10. Unsubstituted phenol (**4g**) and naphth-2-ol (**4h**) also give similar high regioselectivity (87:13 and 86:14, respectively). These results indicate that the effect of substituent position cannot be described simply in terms of steric bulk. The TBDPS system gives similar lower regioselectivity with 2,5-dimethylphenol (**4a**; **5**:**6** = 72:28) and 3,5-dimethylphenol (**4b**; 70:30), whereas 4-methylphenol (**4c**) gives, unexpectedly, the opposite preference (27:73). Unsubstituted phenol (**4g**) and naphth-2-ol (**4h**) also give only moderate selectivity (46:54 and 54:46, respectively). These results suggest that the effect of the substituent cannot be described simply in terms of steric bulk. Instead, both the electronic properties and the spatial orientation of the nucleophile appear to contribute to the relative rates of the competing nucleophilic attack pathways. In particular, the *para* substituent in **4c** is remote from the phenolic oxygen and is therefore unlikely to control the reaction through direct steric shielding of the nucleophilic site. Its principal effect may instead be associated with modulation of the electronic character of the phenol nucleophile and, in combination with the conformation imposed by the protecting group, with the relative stabilization of the transition states leading to **5** and **6**. The pronounced difference between **4c** and **4a**/**4b** for the TBDPS series, together with the absence of such a pronounced difference in the TBDMS series, further suggests that the relationship between nucleophile structure and regioselectivity is not governed by steric demand alone.

A particularly informative comparison is provided by **4c** and **4i**. Whereas *para*-cresol (**4c**) is relatively electron-rich and reacts with the TBDMS donor to give predominantly **5c** (**5**:**6** = 90:10), 4-chlorophenol (**4i**) is less nucleophilic because of the electron-withdrawing effect of the chlorine substituent and gives a substantially less selective product distribution (**5**:**6**:**7** = 59:30:11). The lower nucleophilic of **4i** may result in slower nucleophilic attack, increasing the lifetime of the η^3^-π-allyl palladium intermediate and thereby allowing competitive palladium-mediated isomerization to become more important. This interpretation is consistent with the formation of the additional α-anomer **7i** observed with **4i**.

In contrast, the more nucleophilic **4c** may intercept the π-allyl intermediate more rapidly, thereby suppressing the competing isomerization pathway. Thus, the difference between **4c** and **4i** is consistent with an electronic effect on the rate of C–O bond formation. The experimental data nevertheless indicate that electronic and conformational effects operate simultaneously, and a purely electronic assignment cannot be established from the present dataset alone.

The optimization studies further demonstrate that the reaction outcome is sensitive to the ligand and solvent environment. Rather than acting as independent parameters, these factors are likely to influence the electronic and coordination properties of the palladium centre and, consequently, the relative rates of the elementary steps involved in the catalytic cycle. The superior performance of bidentate phosphine ligands compared with the monodentate PPh_3_ system suggests that the coordination environment around palladium plays an important role in controlling the reaction pathway. Similarly, the differences observed upon changing the solvent indicate that the reaction is sensitive to the medium in which the palladium intermediates and transition states are formed. These effects may arise from changes in the electronic properties and coordination state of Pd, as well as from differential stabilization of the π-allyl palladium intermediate and the transition states associated with nucleophilic attack. Thus, the ligand and solvent effects observed during optimization are consistent with a mechanism in which the regioselectivity is governed by the relative rates of competing pathways rather than by a single steric or electronic factor.

Overall, the combined results support a mechanistic picture in which the **5**:**6** ratio is determined by the competition between nucleophilic attack at the two accessible positions of the π-allyl palladium intermediate and its reversible isomerization. The TBDMS/TBDPS protecting-group effect is therefore best described as a conformational and steric modulation of this competition rather than as a simple requirement for a bulky substituent. Likewise, the lower regioselectivity observed with electron-deficient chlorophenols is consistent with slower nucleophilic attack and a longer-lived palladium intermediate, thereby providing greater opportunity for competing isomerization. The present data are consistent with this interpretation, although distinguishing the individual contributions of electronic, steric, and conformational effects would require additional kinetic or computational studies.

**Scheme 5:**
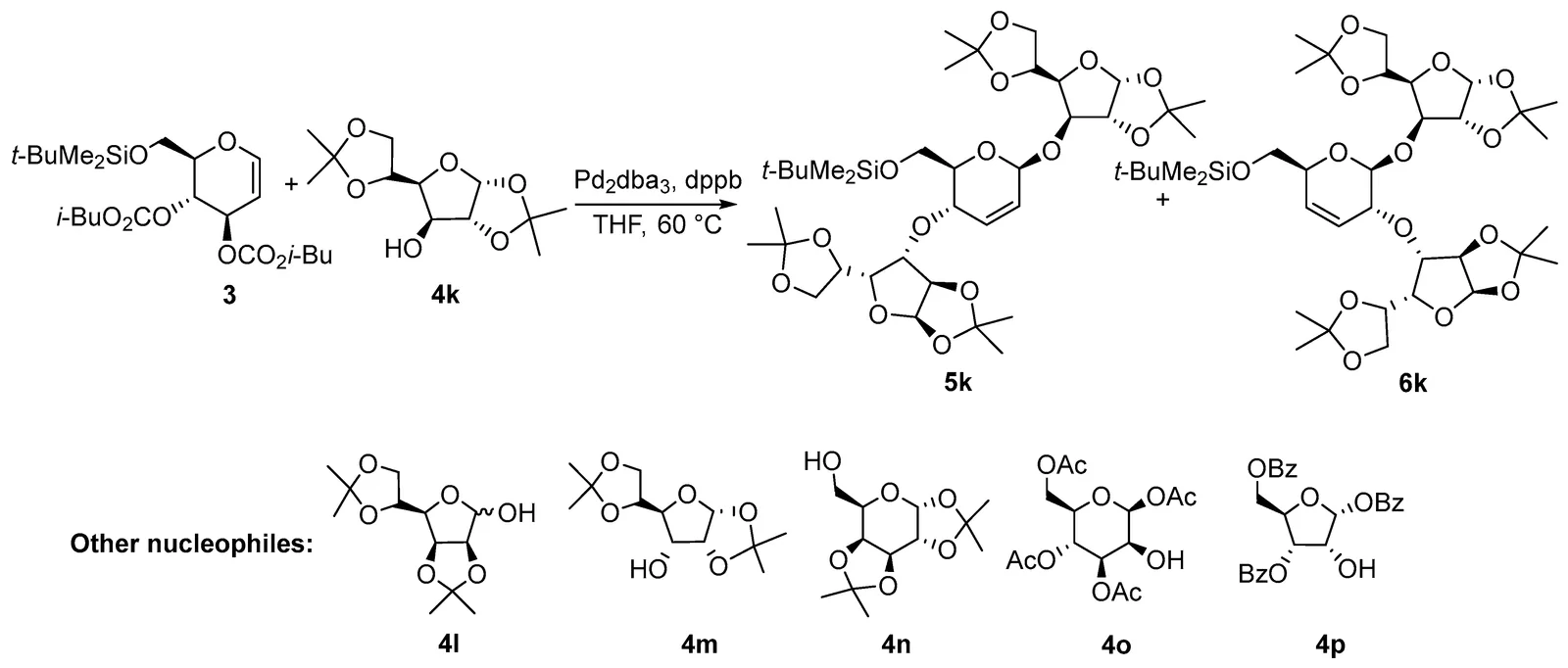
Alkoxylation reaction of dicarbonate **3** with selected monosaccharide nucleophiles.

In the next stage of the study, the developed methodology was extended toward more complex nucleophiles, namely monosaccharides bearing a free hydroxyl group, in order to evaluate its applicability to the synthesis of trisaccharide architectures (Scheme 5). The investigated reactions revealed a pronounced influence of the position and configuration of the free hydroxyl group, as well as the nature of the protecting groups, on both the course and efficiency of the transformation.

Reaction of dicarbonate **3** with 1,2:5,6-di-*O*-isopropylidene-α-d-glucofuranose (**4k**), containing a free secondary hydroxyl group at C-3 (Scheme 5), afforded two unsaturated trisaccharide products, namely the 2,3-unsaturated derivative **5k** and its 3,4-unsaturated isomer **6k**, in 64% and 22% yields, respectively (**5k**:**6k** = 74:26). A similar outcome was observed for 2,3:5,6-di-*O*-isopropylidene-d-mannofuranose, bearing a free anomeric hydroxyl group, which provided trisaccharides **5l** and **6l** in a comparable product ratio (**5l**:**6l** = 72:28) but with a higher combined yield of 92%. In contrast, 1,2:5,6-di-*O*-isopropylidene-α-d-allofuranose (**4m**), differing only in the configuration at C-3 relative to glucofuranose **4k**, proved completely unreactive under the applied conditions. The lack of reactivity is most likely associated with steric shielding of the hydroxyl group by the 1,2-*O*-isopropylidene moiety, preventing effective nucleophilic attack. Similarly, no reaction was observed for 1,2:3,4-di-*O*-isopropylidene-α-d-galactopyranose (**4n**), containing a primary hydroxyl group at C-6, indicating that primary hydroxyl groups are insufficiently reactive in this transformation. The influence of protecting groups was further examined using ester-protected monosaccharides. Both tetra-*O*-acetyl derivative **4o** and tri-*O*-benzoyl-α-d-ribofuranose (**4p**) failed to undergo the reaction, and only starting materials were recovered. Overall, the study demonstrated that monosaccharides bearing ether-type protecting groups and free secondary hydroxyl groups are suitable nucleophiles for this transformation, whereas ester-protected sugars and substrates containing primary hydroxyl groups remain unreactive under these conditions.

## 3. Materials and Methods

Commercially available chemicals used in this work were purchased from Merck (Darmstadt, Germany) and were used as supplied, without additional purification. NMR spectra were recorded in CDCl_3_ on a Bruker Avance III (Bruker, Rheinstetten, Germany) (600 MHz for ^1^H NMR, 150 MHz for ^13^C NMR); coupling constants are reported in hertz (Hz). The chemical shift values were expressed in ppm (parts per million) with tetramethylsilane (TMS) as an internal reference. Chromatographic purification of compounds was achieved with 230–400 mesh size silica gel. The progress of reactions was monitored by silica gel thin-layer chromatography plates (Merck TLC Silicagel 60 F254, Merck, Darmstadt, Germany). The optical rotations were measured using an Anton Paar MCP 500 polarimeter (Anton Paar, Graz, Austria). Melting points measured on the DigiMelt apparatus are uncorrected. THF was distilled from sodium/benzophenone and kept under an argon atmosphere. Reactions involving palladium complexes were carried out in a Schlenk tube under an argon atmosphere.

### 3.1. Synthesis of Unsaturated Dicarbonate ***3***

#### 3.1.1. 6-*O*-*tert*-Butyldimethylsilyl-d-glucal (**2**)

To a solution of d-glucal **1** (2.55 g, 17.5 mmol), imidazole (0.68 g, 1 mmol), and triethylamine (2.9 mL) in dichloromethane (150 mL) under an argon atmosphere, *tert*-butyldimethylsilyl chloride (3.16 g, 21.0 mmol) was added dropwise over 30 min at room temperature. The reaction flask was fitted with a reflux condenser equipped with a drying tube filled with anhydrous calcium chloride. Stirring was continued at room temperature for 24 h, while the progress of the reaction was monitored by thin-layer chromatography (TLC). After completion of the reaction, the solution was washed three times with water. The organic phase was dried over anhydrous magnesium sulfate. After filtration of the drying agent, the solvent was removed under reduced pressure, and the crude product was purified by flash chromatography using a mixture of hexane and ethyl acetate (1:1) as the eluent.

Colorless oil, 3.96 g, 87% yield; R*_f_* = 0.42 (hexane/ethyl acetate, 1:1); [α]_D_^20^ = +1.2 (*c* 1.0, CHCl_3_), {Lit. [42]: [α]_D_^20^ = 6.0 (*c* 1.0, CHCl_3_)}; ^1^H-NMR (600 MHz, CDCl_3_) δ 0.11 (s, 6H, 2 × CH_3_), 0.91 (s, 9H, 3 × CH_3_), 2.55 (s, 1H, OH), 3.20 (s, 1H, OH), 3.79–3.81 (m, 2H, H-4, H-5), 3.90–3.92 (m, 1H, H-6), 3.98–4.00 (m, 1H, H-6′), 4.25–4.26 (m, 1H, H-3), 4.73 (dd, 1H, *J* = 6.1, 2.3, H-2), 6.31 (dd, 1H, *J* = 6.1, 1.5, H-1); ^13^C-NMR (150 MHz, CDCl_3_) δ −5.3, −5.2 (2 × CH_3_), 18.4 (Cq), 26.0 (3 × CH_3_), 63.7 (C-6), 69.5 (C-3), 71.8 (C-4), 77.2 (C-5), 102.6 (C-2), 144.3 (C-1).

Spectral data matched that reported by Yadav [42].

#### 3.1.2. 6-*O*-*tert*-Butyldimethylsilyl-3,4-*O*-diisobutyloxycarbonyl-d-glucal (**3**)

A solution of 6-*O*-*tert*-butyldimethylsilyl-d-glucal (2.2 g, 8.48 mmol) and pyridine (2.7 mL, 33.92 mmol) in dichloromethane (100 mL) was cooled in an ice–water bath. Subsequently, isobutyl chloroformate (4.4 mL, 33.92 mmol) was added dropwise over 30 min. The reaction flask was fitted with a reflux condenser equipped with a drying tube filled with calcium chloride. Stirring was continued at room temperature for 24 h, and the progress of the reaction was monitored by TLC analysis. Upon completion of the reaction, water (30 mL) was added, and the mixture was extracted twice with dichloromethane (2 × 30 mL). To remove residual pyridine, the combined organic phases were washed with saturated copper (II) sulfate solution (30 mL) and dried over anhydrous magnesium sulfate. After filtration, the solvent was evaporated under reduced pressure, and the crude product was purified by flash chromatography using a 1:1 mixture of hexane and ethyl acetate as the eluent.

Yellow oil, 3.67 g, 94% yield; R*_f_* = 0.90 (hexane/ethyl acetate, 1:1); [α]_D_^20^ = −16.0 (*c* 1.0, CHCl_3_); ^1^H-NMR (600 MHz, CDCl_3_) δ 0.06, 0.06 (2s, 6H, CH_3_ each), 0.89 (s, 9H, 3 × CH_3_), 0.93, 0.94, 0.94, 0.95 (4s, 12H, CH_3_ each), 1.92–2.02 (m, 2H, 2 × CH), 3.85–3.87 (m, 2H, C-6, C-6′), 3.89–3.97 (m, 4H, 2 × CH_2_), 4.14–4.19 (m, 1H, H-5), 4.87 (dd, 1H, *J* = 6.2, 3.4, H-2), 5.19 (dd, 1H, *J* = 6.8, 5.3, H-4), 5.24 (dd, 1H, *J* = 6.8, 3.4, H-3), 6.48 (d, 1H, *J* = 6.2, H-1); ^13^C-NMR (150 MHz, CDCl_3_) δ −5.4, −5.3 (2 × CH_3_), 18.4 (Cq), 19.0 (2 × CH_3_), 19.0 (2 × CH_3_), 25.9 (3 × CH_3_), 27.9 (CH), 27.9 (CH), 61.0 (C-6), 70,8 (C-3), 71.0 (C-4), 74.4 (OCH_2_), 74.7 (OCH_2_), 76.5 (C-5), 97.8 (C-2), 146.5 (C-1), 154.2 (CO), 154.9 (CO); Elemental analysis: C_22_H_40_O_8_Si (460.64 g/mol) calculated: C% 57.36, H% 8.75; found: C% 57.43, H% 8.77.

### 3.2. General Procedure for the Pd(0)-Catalysed Alkylation Reaction

The catalytic system was prepared by stirring Pd_2_(dba)_3_ (22.9 mg, 0.025 mmol) and the ligand (0.055 mmol or 0.11 mmol) in an appropriate anhydrous solvent (3 mL) for 0.5 h in a Schlenk tube under argon. This resulting solution was transferred under argon to a Schlenk tube containing the unsaturated carbohydrate (1 mmol) and nucleophile (3 mmol) in the same solvent (3 mL). The reaction mixture was stirred at 25 °C (or 60 °C), and the progress of the reaction was monitored by TLC. After the appropriate reaction time, the solvent was evaporated under reduced pressure, and the residue was purified by column chromatography on silica gel to afford the corresponding *O*-glycosides.

#### 3.2.1. Reaction of Dicarbonate **3** with 2,5-Dimethylphenol (**4a**)

##### 2,5-Dimethylphenyl 6-*O*-*tert*-Butyldimethylsilyl-4-*O*-(2,5-dimethylphenyl)-2,3-dideoxy-β-d-*erythro*-hex-2-enopyranoside (**5a**)

Yellowish oil, 80 mg, 68% yield; R*_f_* = 0.78 (hexane/ethyl acetate, 10:1); ^1^H-NMR (600 MHz, CDCl_3_) δ 0.01, 0.04 (2s, 6H, CH_3_ each), 0.88 (s, 9H, 3 × CH_3_), 2.17, 2.18, 2.30, 2.31 (4s, 12H, CH_3_ each), 3.79 (dd, 1H, *J* = 10.7, 5.2, H-6), 3.95 (dd, 1H, *J* = 10.7, 6.4, H-6′), 4.19 (ddd ≈ dt, 1H, *J* = 6.1, 5.2, 5.2, H-5), 4.84 (ddd ≈ dt, 1H, *J* = 5.2, 3.7, 3.7, H-4), 5.79 (s, 1H, H-1), 6.05 (dd, 1H, *J* = 10.2, 1.1, H-2), 6.21 (dd, 1H, *J* = 10.2, 3.7, H-3), 6.68–6.82 (m, 3H, H_ar_), 7.00–7.06 (m, 3H, H_ar_); ^13^C-NMR (150 MHz, CDCl_3_) δ −5.3, −5.2 (2 × CH_3_), 16.1, 16.2 (2 × CH_3_), 18.5 (Cq), 21.4, 21.5 (2 × CH_3_), 26.0 (3 × CH_3_), 63.2 (C-6), 67.8 (C-4), 76.7 (C-5), 93.7 (C-1), 114.1, 115.6, 121.8, 122.6, 124.3, 124.8 (C_ar_), 127.6 (C-3), 128.8 (C-2), 130.5, 130.9, 136.7, 136.8, 155.4, 155.7 (C_ar_).

##### 2,5-Dimethylphenyl 6-*O*-*tert*-Butyldimethylsilyl-2-*O*-(2,5-dimethylphenyl)-3,4-dideoxy-β-d-*erythro*-hex-3-enopyranoside (**6a**)

Yellowish oil, 13 mg, 11% yield; R*_f_* = 0.78 (hexane/ethyl acetate, 10:1); ^1^H-NMR (600 MHz, CDCl_3_) δ 0.00, 0.05 (2s, 6H, CH_3_ each), 0.89 (s, 9H, 3 × CH_3_), 2.07, 2.13, 2.30, 2.31 (4s, 12H, CH_3_ each), 3.62 (dd, 1H, *J* = 10.0, 6.8, H-6), 3.76 (dd, 1H, *J* = 10.0, 6.0, H-6′), 4.46–4.51 (m, 1H, H-5), 4.91–4.95 (m, 1H, H-2), 5.37 (d, 1H, *J* = 5.6, H-1), 5.98 (ddd ≈ dt, 1H, *J* = 10.3, 2.2, 2.2, H-4), 6.03 (ddd ≈ dt, 1H, *J* = 10.3, 1.2, 1.2, H-3), 6.68–6.81 (m, 2H, H_ar_), 6.83–6.91 (m, 2H, H_ar_), 6.96–7.01 (m, 2H, H_ar_); ^13^C-NMR (150 MHz, CDCl_3_) δ −5.3, −5.2 (2 × CH_3_), 16.0, 16.1 (2 × CH_3_), 18.4 (Cq), 21.4, 21.5 (2 × CH_3_), 26.0 (3 × CH_3_), 65.7 (C-6), 72.9 (C-2), 75.3 (C-5), 99.3 (C-1), 114.7, 115.7, 121.9, 122.9 (C_ar_), 125.1 (C-4), 128.8 (C_ar_), 130.0 (C-3), 130.8, 131.0, 132.7, 136.6, 136.7, 155.5, 156.1 (C_ar_).

Elemental analysis for the mixture of both isomers: C_28_H_40_O_4_Si (468.71 g/mol) calculated: C% 71.75, H% 8.60; found: C% 71.81, H% 8.52.

#### 3.2.2. Reaction of Dicarbonate **3** with 3,5-Dimethylphenol (**4b**)

##### 3,5-Dimethylphenyl 6-*O*-*tert*-Butyldimethylsilyl-4-*O*-(3,5-dimethylphenyl)-2,3-dideoxy-β-d-*erythro*-hex-2-enopyranoside (**5b**)

Yellowish oil, 80 mg, 68% yield; R*_f_* = 0.76 (hexane/ethyl acetate, 10:1); ^1^H-NMR (600 MHz, CDCl_3_) δ 0.05, 0.07 (2s, 6H, CH_3_ each), 0.90 (s, 9H, 3 × CH_3_), 2.29, 2.30 (2s, 12H, 2 × CH_3_ each), 3.79 (dd, 1H, *J* = 10.7, 5.0, H-6), 3.94 (dd, 1H, *J* = 10.7, 7.3, H-6′), 4.18–4.23 (m, 1H, H-5), 4.85 (dd, 1H, *J* = 4.0, 3.9, H-4), 5.81 (s, 1H, H-1), 6.06 (dd, 1H, *J* = 10.2, 1.1, H-2), 6.23 (dd, 1H, *J* = 10.2, 4.0, H-3), 6.63 (s, 1H, H_ar_), 6.66 (s, 2H, H_ar_), 6.68 (s, 1H, H_ar_), 6.74 (s, 2H, H_ar_); ^13^C-NMR (150 MHz, CDCl_3_) δ −5.3, −5.2 (2 × CH_3_), 18.5 (Cq), 21.5, 21.6 (4 × CH_3_), 26.1 (3 × CH_3_), 63.2 (C-6), 67.0 (C-4), 76.2 (C-5), 93.1 (C-1), 113.7, 114.5, 123.3, 124.0 (C_ar_), 127.0 (C-3), 128.8 (C-2), 139.3, 139.5, 157.3, 157.7 (C_ar_).

##### 3,5-Dimethylphenyl 6-*O*-*tert*-Butyldimethylsilyl-2-*O*-(3,5-dimethylphenyl)-3,4-dideoxy-β-d-*erythro*-hex-3-enopyranoside (**6b**)

Yellowish oil, 8 mg, 7% yield; R*_f_* = 0.76 (hexane/ethyl acetate, 10:1); ^1^H-NMR (600 MHz, CDCl_3_) δ 0.02, 0.04 (2s, 6H, CH_3_ each), 0.89 (s, 9H, 3 × CH_3_), 2.28, 2.29 (2s, 12H, 2 × CH_3_ each), 3.62 (dd, 1H, *J* = 9.9, 7.1, H-6), 3.74 (dd, 1H, *J* = 9.9, 6.0, H-6′), 4.45–4.49 (m, 1H, H-5), 4.83–4.87 (m, 1H, H-2), 5.42 (d, 1H, *J* = 4.9, H-1), 6.01 (ddd ≈ dt, 1H, *J* = 10.1, 2.5, 2.5, H-4), 6.10 (ddd ≈ dt, 1H, *J* = 10.1, 1.5, 1.5, H-3), 6.62–6.68 (m, 4H, H_ar_), 6.74 (s, 2H, H_ar_); ^13^C-NMR (150 MHz, CDCl_3_) δ −5.3, −5.2 (2 × CH_3_), 19.1 (Cq), 21.5, 21.6 (4 × CH_3_), 26.0 (3 × CH_3_), 65.9 (C-6), 71.8 (C-2), 74.8 (C-5), 98.3 (C-1), 114.1, 114.6, 123.4, 124.2 (C_ar_), 124.3 (C-4), 130.5 (C-3), 139.2, 139.4, 157.2, 157.9 (C_ar_).

Elemental analysis for the mixture of both isomers: C_28_H_40_O_4_Si (468.71 g/mol) calculated: C% 71.75, H% 8.60; found: C% 71.78, H% 8.56.

#### 3.2.3. Reaction of Dicarbonate **3** with 4-Methylphenol (**4c**)

##### 4-Methylphenyl 6-*O*-*tert*-Butyldimethylsilyl-4-*O*-(4-methylphenyl)-2,3-dideoxy-β-d-*erythro*-hex-2-enopyranoside (**5c**)

Yellowish oil, 86 mg, 78% yield; R*_f_* = 0.70 (hexane/ethyl acetate, 10:1); ^1^H-NMR (600 MHz, CDCl_3_) δ 0.03, 0.05 (2s, 6H, CH_3_ each), 0.91 (s, 9H, 3 × CH_3_), 2.29, 2.30 (2s, 6H, CH_3_ each), 3.75 (dd, 1H, *J* = 10.6, 4.9, H-6), 3.94 (dd, 1H, *J* = 10.6, 7.4, H-6′), 4.17–4.25 (m, 1H, H-5), 4.78–4.87 (m, 1H, H-4), 5.79 (s, 1H, H-1), 6.08 (dd, 1H, *J* = 10.1, 1.1, H-2), 6.22 (dd, 1H, *J* = 10.1, 3.5, H-3), 6.92–6.96 (m, 2H, H_ar_), 6.99–7.03 (m, 2H, H_ar_), 7.06–7.11 (m, 4H, H_ar_); ^13^C-NMR (150 MHz, CDCl_3_) δ −5.3, −5.3 (2 × CH_3_), 18.4 (Cq), 20.6, 20.7 (2 × CH_3_), 26.0 (3 × CH_3_), 63.2 (C-6), 67.3 (C-4), 76.1 (C-5), 93.3 (C-1), 115.8, 116.7 (C_ar_), 127.0 (C-3), 128.8 (C-2), 130.0, 130.1, 130.7, 131.6, 155.1, 155.5 (C_ar_).

##### 4-Methylphenyl 6-*O*-*tert*-Butyldimethylsilyl-2-*O*-(4-methylphenyl)-3,4-dideoxy-β-d-*erythro*-hex-3-enopyranoside (**6c**)

Yellowish oil, 10 mg, 9% yield; R*_f_* = 0.70 (hexane/ethyl acetate, 10:1); ^1^H-NMR (600 MHz, CDCl_3_) δ 0.03, 0.04 (2s, 6H, CH_3_ each), 0.89 (s, 9H, 3 × CH_3_), 2.29, 2.30 (2s, 6H, CH_3_ each), 3.61 (dd, 1H, *J* = 9.9, 7.3, H-6), 3.72 (dd, 1H, *J* = 9.9, 5.9, H-6′), 4.42–4.48 (m, 1H, H-5), 4.78–4.87 (m, 1H, H-2), 5.38 (d, 1H, *J* = 5.0, H-1), 6.00 (ddd ≈ dt, 1H, *J* = 10.3, 2.3, 2.3, H-4), 6.10 (ddd ≈ dt, 1H, *J* = 10.3, 1.3, 1.3, H-3), 6.89–6.93 (m, 4H, H_ar_), 7.05–7.09 (m, 4H, H_ar_); ^13^C-NMR (150 MHz, CDCl_3_) δ −5.3, −5.2 (2 × CH_3_), 19.1 (Cq), 20.6, 20.7 (2 × CH_3_), 26.0 (3 × CH_3_), 65.7 (C-6), 72.3 (C-2), 74.8 (C-5), 98.8 (C-1), 116.3, 116.8 (C_ar_), 124.1 (C-4), 130.0 (C-3), 130.1, 130.5131.0, 131.8, 155.1, 155.8 (C_ar_).

Elemental, analysis for the mixture of both isomers: C_26_H_36_O_4_Si (440.66 g/mol) calculated: C% 70.87, H% 8.24; found: C% 70.69, H% 8.06.

#### 3.2.4. Reaction of Dicarbonate **3** with 3-Methoxyphenol (**4d**)

##### 3-Methoxyphenyl 6-*O*-*tert*-Butyldimethylsilyl-4-*O*-(3-methoxyphenyl)-2,3-dideoxy-β-d-*erythro*-hex-2-enopyranoside (**5d**)

Yellowish oil, 66 mg, 56% yield; R*_f_* = 0.32 (hexane/ethyl acetate, 10:1); ^1^H-NMR (600 MHz, CDCl_3_) δ 0.04, 0.05 (2s, 6H, CH_3_ each), 0.90 (s, 9H, 3 × CH_3_), 3.77 (dd, 1H, *J* = 10.0, 5.0, H-6), 3.78, 3.79 (2s, 6H, CH_3_ each), 3.94 (dd, 1H, *J* = 10.0, 7.7, H-6′), 4.23 (ddd ≈ dt, 1H, *J* = 7.7, 4.1, 4.1, H-5), 4.84–4.89 (m, 1H, H-4), 5.83 (s, 1H, H-1), 6.10 (dd, 1H, *J* = 10.2, 1.1, H-2), 6.25 (dd, 1H, *J* = 10.2, 4.0, H-3), 6.51–6.56 (m, 1H, H_ar_), 6.57–6.61 (m, 2H, H_ar_), 6.64–6.68 (m, 2H, H_ar_), 6.70–6.74 (m, 2H, H_ar_), 7.15–7.22 (m, 1H, H_ar_); ^13^C-NMR (150 MHz, CDCl_3_) δ −5.3, −5.3 (2 × CH_3_), 18.4 (Cq), 26.0 (3 × CH_3_), 55.4 (2 × OCH_3_), 63.2 (C-6), 67.1 (C-4), 76.1 (C-5), 92.9 (C-1), 102.6, 103.1, 107.3, 107.5, 108.0, 108.9 (C_ar_), 126.8 (C-3), 128.7 (C-2), 130.0, 130.1, 158.3, 158.8, 160.9, 161.2 (C_ar_).

##### 3-Methoxyphenyl 6-*O*-*tert*-Butyldimethylsilyl-2-*O*-(3-methoxyphenyl)-3,4-dideoxy-β-d-*erythro*-hex-3-enopyranoside (**6d**)

Yellowish oil, 10 mg, 8% yield; R*_f_* = 0.32 (hexane/ethyl acetate, 10:1); ^1^H-NMR (600 MHz, CDCl_3_) δ 0.03, 0.04 (2s, 6H, CH_3_ each), 0.89 (s, 9H, 3 × CH_3_), 3.62 (dd, 1H, *J* = 10.0, 6.8, H-6), 3.74 (dd, 1H, *J* = 10.0, 6.6, H-6′), 3.77, 3.78 (2s, 6H, CH_3_ each), 4.45–4.49 (m, 1H, H-5), 4.87–4.90 (m, 1H, H-2), 5.45 (d, 1H, *J* = 4.8, H-1), 6.01 (ddd ≈ dt, 1H, *J* = 10.4, 2.4, 2.4, H-4), 6.14 (ddd ≈ dt, 1H, *J* = 10.4, 1.2, 1.2, H-3), 6.54–6.57 (m, 1H, H_ar_), 6.57–6.62 (m, 2H, H_ar_), 6.63–6.67 (m, 2H, H_ar_), 6.70–6.74 (m, 1H, H_ar_), 7.15–7.22 (m, 2H, H_ar_); ^13^C-NMR (150 MHz, CDCl_3_) δ −5.3, −5.2 (2 × CH_3_), 18.4 (Cq), 26.0 (3 × CH_3_), 55.4 (2 × OCH_3_), 65.7 (C-6), 71.8 (C-2), 74.9 (C-5), 98.3 (C-1), 102.7, 103.2, 108.2, 108.3, 109.0 (C_ar_), 123.7 (C-4), 130.0 (C-3), 130.1, 130.8, 158.3, 159.0, 160.8, 161.1 (C_ar_).

Elemental analysis for the mixture of both isomers: C_26_H_36_O_6_Si (472.65 g/mol) calculated: C% 66.07, H% 7.68; found: C% 66.04, H% 7.55.

#### 3.2.5. Reaction of Dicarbonate **3** with 3,5-Dimethoxyphenol (**4e**)

##### 3,5-Dimethoxyphenyl 6-*O*-*tert*-Butyldimethylsilyl-4-*O*-(3,5-dimethoxyphenyl)-2,3-dideoxy-β-d-*erythro*-hex-2-enopyranoside (**5e**)

Yellowish oil, 98 mg, 66% yield; R*_f_* = 0.31 (hexane/ethyl acetate, 6:1); ^1^H-NMR (600 MHz, CDCl_3_) δ 0.04, 0.05 (2s, 6H, CH_3_ each), 0.89 (s, 9H, 3 × CH_3_), 3.75, 3.76 (2s, 12H, 2 × CH_3_ each), 3.78 (dd, 1H, *J* = 10.6, 4.7, H-6), 3.93 (dd, 1H, *J* = 10.6, 7.6, H-6′), 4.22 (ddd ≈ dt, 1H, *J* = 7.6, 4.7, 4.7, H-5), 4.81–4.85 (m, 1H, H-4), 5.81 (s, 1H, H-1), 6.08 (dd, 1H, *J* = 10.2, 1.1, H-2), 6.11 (t, 1H, *J* = 1.9, H_ar_), 6.16 (t, 1H, *J* = 1.9, H_ar_), 6.21 (d, 2H, *J* = 1.9, H_ar_), 6.25 (dd, 1H, *J* = 10.2, 4.1, H-3), 6.29 (d, 2H, *J* = 1.9, H_ar_); ^13^C-NMR (150 MHz, CDCl_3_) δ −5.3, −5.2 (2 × CH_3_), 18.5 (Cq), 26.0 (3 × CH_3_), 55.4, 55.5 (2 × OCH_3_), 63.1 (C-6), 69.9 (C-4), 76.0 (C-5), 92.7 (C-1), 93.6, 94.6, 94.7, 95.4 (C_ar_), 126.7 (C-3), 128.7 (C-2), 158.9, 159.3, 161.5, 161.8 (C_ar_).

##### 3,5-Dimethoxyphenyl 6-*O*-*tert*-Butyldimethylsilyl-2-*O*-(3,5-dimethoxyphenyl)-3,4-dideoxy-β-d-*erythro*-hex-3-enopyranoside (**6e**)

Yellowish oil, 8 mg, 12% yield; R*_f_* = 0.31 (hexane/ethyl acetate, 6:1); ^1^H-NMR (600 MHz, CDCl_3_) δ 0.02, 0.04 (2s, 6H, CH_3_ each), 0.88 (s, 9H, 3 × CH_3_), 3.60 (dd, 1H, *J* = 10.0, 7.4, H-6), 3.75, 3.76 (2s, 12H, 2 × CH_3_ each), 3.93 (dd, 1H, *J* = 10.0, 5.9, H-6′), 4.44–4.48 (m, 1H, H-5), 4.81–4.84 (m, 1H, H-2), 5.44 (d, 1H, *J* = 4.7, H-1), 6.01 (ddd ≈ dt, 1H, *J* = 10.2, 2.6, 2.6, H-4), 6.12 (t, 1H, *J* = 1.9, H_ar_), 6.14 (ddd ≈ dt, 1H, *J* = 10.2, 1.2, 1.2, H-3), 6.16 (t, 1H, *J* = 1.9, H_ar_), 6.18 (d, 2H, *J* = 1.9, H_ar_), 6.22 (d, 2H, *J* = 1.9, H_ar_); ^13^C-NMR (150 MHz, CDCl_3_) δ −5.3, −5.3 (2 × CH_3_), 18.4 (Cq), 25.9 (3 × CH_3_), 55.4, 55.5 (2 × OCH_3_), 65.6 (C-6), 71.5 (C-2), 74.7 (C-5), 94.1, 94.8, 94.9, 95.5 (C_ar_), 97.9 (C-1), 123.3 (C-4), 130.9 (C-3), 158.8, 159.5, 161.5, 161.7 (C_ar_).

Elemental analysis for the mixture of both isomers: C_28_H_40_O_8_Si (532.71 g/mol) calculated: C% 63.13, H% 7.57; found: C% 63.04, H% 7.41.

#### 3.2.6. Reaction of Dicarbonate **3** with 2,6-Dimethoxyphenol (**4f**)

##### 2,6-Dimethoxyphenyl 6-*O*-*tert*-Butyldimethylsilyl-4-*O*-(2,6-dimethoxyphenyl)-2,3-dideoxy-β-d-*erythro*-hex-2-enopyranoside (**5f**)

Yellowish oil, 7 mg, 5% yield; R*_f_* = 0.24 (hexane/ethyl acetate, 6:1); ^1^H-NMR (600 MHz, CDCl_3_) δ −0.11, −0.03 (2s, 6H, CH_3_ each), 0.74 (s, 9H, 3 × CH_3_), 3.82 (s, 12H, 4 × CH_3_), 3.94 (dd, 1H, *J* = 10.9, 4.2, H-6), 3.99 (dd, 1H, *J* = 10.9, 7.5, H-6′), 4.14–4.18 (m, 1H, H-5), 4.54 (dd, 1H, *J* = 4.2, 3.8, H-4), 5.74 (s, 1H, H-1), 6.22 (dd, 1H, *J* = 10.2, 1.3, H-2), 6.26 (dd, 1H, *J* = 10.2, 3.8, H-3), 6.54–6.60 (m, 4H, H_ar_), 6.94–7.00 (m, 2H, H_ar_).

Elemental analysis: C_28_H_40_O_8_Si (532.71 g/mol) calculated: C% 63.13, H% 7.57; found: C% 63.11, H% 7.32.

#### 3.2.7. Reaction of Dicarbonate **3** with Phenol (**4g**)

##### Phenyl 6-*O*-*tert*-Butyldimethylsilyl-4-*O*-phenyl-2,3-dideoxy-β-d-*erythro*-hex-2-enopyranoside (**5g**)

Yellowish oil, 71 mg, 69% yield; R*_f_* = 0.75 (hexane/ethyl acetate, 10:1); ^1^H-NMR (600 MHz, CDCl_3_) δ 0.02, 0.04 (2s, 6H, CH_3_ each), 0.89 (s, 9H, 3 × CH_3_), 3.75 (dd, 1H, *J* = 10.5, 4.9, H-6), 3.94 (dd, 1H, *J* = 10.5, 7.5, H-6′), 4.21–4.25 (m, 1H, H-5), 4.86–4.89 (m, 1H, H-4), 5.83 (s, 1H, H-1), 6.10 (dd, 1H, *J* = 10.2, 1.0, H-2), 6.23 (dd, 1H, *J* = 10.2, 4.0, H-3), 6.95–7.05 (m, 4H, H_ar_), 7.09–7.11 (m, 2H, H_ar_), 7.26–7.30 (m, 4H, H_ar_); ^13^C-NMR (150 MHz, CDCl_3_) δ −5.5, −5.4 (2 × CH_3_), 18.2 (Cq), 25.8 (3 × CH_3_), 63.0 (C-6), 66.9 (C-4), 75.9 (C-5), 92.8 (C-1), 115.7, 116.6, 121.3, 122.1 (C_ar_), 126.8 (C-3), 128.6 (C-2), 129.4, 129.6, 157.0, 157.5 (C_ar_).

##### Phenyl 6-*O*-*tert*-Butyldimethylsilyl-2-*O*-phenyl-3,4-dideoxy-β-d-*erythro*-hex-3-enopyranoside (**6g**)

Yellowish oil, 11 mg, 11% yield; R*_f_* = 0.75 (hexane/ethyl acetate, 10:1); ^1^H-NMR (600 MHz, CDCl_3_) δ 0.02, 0.03 (2s, 6H, CH_3_ each), 0.87 (s, 9H, 3 × CH_3_), 3.61 (dd, 1H, *J* = 10.0, 7.1, H-6), 3.72 (dd, 1H, *J* = 10.0, 6.1, H-6′), 4.45–4.49 (m, 1H, H-5), 4.89–4.93 (m, 1H, H-2), 5.45 (d, 1H, *J* = 5.0, H-1), 6.01 (ddd ≈ dt, 1H, *J* = 10.3, 2.5, 2.5, H-4), 6.12 (d, 1H, *J* = 10.3, H-3), 6.95–7.05 (m, 6H, H_ar_), 7.26–7.30 (m, 4H, H_ar_); ^13^C-NMR (150 MHz, CDCl_3_) δ −5.5, −5.4 (2 × CH_3_), 18.2 (Cq), 25.8 (3 × CH_3_), 65.5 (C-6), 71.8 (C-2), 74.8 (C-5), 98.3 (C-1), 116.2, 116.7, 121.5, 122.4 (C_ar_), 123.8 (C-4), 129.4, 129.6 (C_ar_), 130.5 (C-3), 157.0, 157.7 (C_ar_).

Elemental analysis for the mixture of both isomers: C_24_H_32_O_4_Si (412.60 g/mol) calculated: C% 69.87, H% 7.82; found: C% 69.90, H% 7.72.

#### 3.2.8. Reaction of Dicarbonate **3** with Naphth-2-ol (**4h**)

##### Naphth-2-yl 6-*O*-*tert*-Butyldimethylsilyl-4-*O*-(naphth-2-yl)-2,3-dideoxy-β-d-*erythro*-hex-2-enopyranoside (**5h**)

Yellowish oil, 52 mg, 41% yield; R*_f_* = 0.64 (hexane/ethyl acetate, 20:1); ^1^H-NMR (600 MHz, CDCl_3_) δ 0.05, 0.07 (2s, 6H, CH_3_ each), 0.91 (s, 9H, 3 × CH_3_), 3.83 (dd, 1H, *J* = 10.5, 4.7, H-6), 4.05 (dd, 1H, *J* = 10.5, 7.8, H-6′), 4.36–4.40 (m, 1H, H-5), 5.06–5.09 (m, 1H, H-4), 6.01 (s, 1H, H-1), 6.19 (d, 1H, *J* = 10.2, H-2), 6.35 (dd, 1H, *J* = 10.2, 4.2, H-3), 7.21–7.27 (m, 2H, H_ar_), 7.32–7.38 (m, 3H, H_ar_), 7.40–7.46 (m, 2H, H_ar_), 7.52–7.55 (m, 1H, H_ar_), 7.70–7.80 (m, 6H, H_ar_); ^13^C-NMR (150 MHz, CDCl_3_) δ −5.3, −5.2 (2 × CH_3_), 18.4 (Cq), 26.0 (3 × CH_3_), 63.1 (C-6), 66.9 (C-4), 75.9 (C-5), 92.8 (C-1), 108.5, 110.8, 119.0, 119.3, 123.9, 124.2, 126.4, 126.5 (C_ar_), 126.6 (C-3), 126.9, 127.2, 127.6, 127.7 (C_ar_), 128.8 (C-2), 129.3, 129.4, 129.7, 129.8, 134.5, 134.5, 154.8, 155.3 (C_ar_).

##### Naphth-2-yl 6-*O*-*tert*-Butyldimethylsilyl-2-*O*-(naphth-2-yl)-3,4-dideoxy-β-d-*erythro*-hex-3-enopyranoside (**6h**)

Yellowish oil, 10 mg, 8% yield; R*_f_* = 0.64 (hexane/ethyl acetate, 20:1); ^1^H-NMR (600 MHz, CDCl_3_) δ 0.03, 0.09 (2s, 6H, CH_3_ each), 0.87 (s, 9H, 3 × CH_3_), 3.67 (dd, 1H, *J* = 9.9, 7.1, H-6), 3.77 (dd, 1H, *J* = 9.9, 6.0, H-6′), 4.56–4.60 (m, 1H, H-5), 5.11–5.14 (m, 1H, H-2), 5.68 (d, 1H, *J* = 4.7, H-1), 6.14 (d, 1H, *J* = 10.4, H-4), 6.20 (d, 1H, *J* = 10.4, H-3), 7.21–7.27 (m, 2H, H_ar_), 7.32–7.38 (m, 4H, H_ar_), 7.40–7.46 (m, 2H, H_ar_), 7.70–7.80 (m, 6H, H_ar_); ^13^C-NMR (150 MHz, CDCl_3_) δ −5.4, −5.3 (2 × CH_3_), 18.3 (Cq), 25.9 (3 × CH_3_), 65.6 (C-6), 71.7 (C-2), 74.9 (C-5), 98.3 (C-1), 109.2, 111.0, 119.3, 119.5, 121.5, 122.4 (C_ar_), 123.5 (C-4), 124.0, 124.3, 126.4, 126.5, 126.9, 127.2, 129.4, 129.4, 129.7, 129.8 (C_ar_), 130.8 (C-3), 134.4, 134.5, 154.8, 155.5 (C_ar_).

Elemental analysis for the mixture of both isomers: C_32_H_36_O_4_Si (512.24 g/mol) calculated: C% 74.96, H% 7.08; found: C% 74.78, H% 7.07.

#### 3.2.9. Reaction of Dicarbonate **3** with 4-Chlorophenol (**4i**)

##### 4-Chlorophenyl 6-*O*-*tert*-Butyldimethylsilyl-4-*O*-(4-chlorophenyl)-2,3-dideoxy-β-d-*erythro*-hex-2-enopyranoside (**5i**)

Yellowish oil, 63 mg, 53% yield; R*_f_* = 0.74 (hexane/dichloromethane, 1:1); ^1^H-NMR (600 MHz, CDCl_3_) δ 0.05 (s, 6H, 2 × CH_3_), 0.90 (s, 9H, 3 × CH_3_), 3.72 (dd, 1H, *J* = 9.9, 4.6, H-6), 3.90 (dd, 1H, *J* = 9.9, 7.7, H-6′), 4.20 (ddd ≈ dt, 1H, *J* = 7.7, 4.6, 4.6, H-5), 4.81–4.84 (m, 1H, H-4), 5.80 (s, 1H, H-1), 6.10 (dd, 1H, *J* = 10.3, 1.4, H-2), 6.22 (dd, 1H, *J* = 10.3, 3.7, H-3), 6.97–6.99 (m, 2H, H_ar_), 7.02–7.04 (m, 2H, H_ar_), 7.22–7.27 (m, 4H, H_ar_); ^13^C-NMR (150 MHz, CDCl_3_) δ −5.3, −5.2 (2 × CH_3_), 18.4 (Cq), 25.9 (3 × CH_3_), 63.0 (C-6), 67.3 (C-4), 75.9 (C-5), 92.9 (C-1), 118.1, 118.3, 126.5 (C_ar_), 126.7 (C-3), 127.4 (C_ar_), 128.6 (C-2), 129.5, 129.6, 155.6, 156.2 (C_ar_).

##### 4-Chlorophenyl 6-*O*-*tert*-Butyldimethylsilyl-2-*O*-(4-chlorophenyl)-3,4-dideoxy-β-d-erythro-hex-3-enopyranoside (**6i**)

Yellowish oil, 32 mg, 27% yield; R*_f_* = 0.74 (hexane/dichloromethane, 1:1); ^1^H-NMR (600 MHz, CDCl_3_) δ 0.03, 0.03 (2s, 6H, CH_3_ each), 0.89 (s, 9H, 3 × CH_3_), 3.61 (dd, 1H, *J* = 10.0, 6.7, H-6), 3.71 (dd, 1H, *J* = 10.0, 6.0, H-6′), 4.44–4.48 (m, 1H, H-5), 4.84–4.86 (m, 1H, H-2), 5.34 (d, 1H, *J* = 5.2, H-1), 5.97 (ddd ≈ dt, 1H, *J* = 10.3, 2.5, 2.5, H-4), 6.10 (d, 1H, *J* = 10.3, H-3), 6.93–6.96 (m, 4H, H_ar_), 7.22–7.27 (m, 4H, H_ar_); ^13^C-NMR (150 MHz, CDCl_3_) δ −5.3, −5.3 (2 × CH_3_), 18.4 (Cq), 25.9 (3 × CH_3_), 65.5 (C-6), 72.5 (C-2), 75.2 (C-5), 98.9 (C-1), 117.2, 117.7 (C_ar_), 123.7 (C-4), 126.8, 127.7, 129.5, 129.6 (C_ar_), 130.7 (C-3), 155.6, 156.5 (C_ar_).

##### 4-Chlorophenyl 6-*O*-*tert*-Butyldimethylsilyl-4-*O*-(4-chlorophenyl)-2,3-dideoxy-α-d-*erythro*-hex-2-enopyranoside (**7i**)

Yellowish oil, 12 mg, 10% yield; R*_f_* = 0.74 (hexane/dichloromethane, 1:1); ^1^H-NMR (600 MHz, CDCl_3_) δ 0.01, 0.03 (2s, 6H, CH_3_ each), 0.85 (s, 9H, 3 × CH_3_), 3.82 (dd, 1H, *J* = 11.6, 1.7, H-6), 3.86 (dd, 1H, *J* = 11.6, 3.8, H-6′), 4.07 (ddd, 1H, *J* = 9.1, 3.8, 1.7, H-5), 4.96 (dd, 1H, *J* = 9.1, 1.2, H-4), 5.68 (s, 1H, H-1), 5.97 (d, 1H, *J* = 10.2, H-2), 6.18 (d, 1H, *J* = 10.2, H-3), 6.89–6.92 (m, 2H, H_ar_), 7.05–7.08 (m, 2H, H_ar_), 7.22–7.27 (m, 4H, H_ar_); ^13^C-NMR (150 MHz, CDCl_3_) δ −5.3, −5.2 (2 × CH_3_), 18.5 (Cq), 26.0 (3 × CH_3_), 62.2 (C-6), 68.5 (C-4), 71.4 (C-5), 93.5 (C-1), 117.3, 118.5, 126.6, 127.3 (C_ar_), 128.6 (C-3), 129.5, 129.7 (C_ar_), 130.6 (C-2), 156.1, 156.1 (C_ar_).

Elemental analysis for the mixture of isomers: C_24_H_30_Cl_2_O_4_Si (481.49 g/mol) calculated: C% 59.87, H% 6.28; found: C% 59.76, H% 6.09.

#### 3.2.10. Reaction of Dicarbonate **3** with 2-Chlorophenol (**4j**)

##### 2-Chlorophenyl 6-*O*-*tert*-Butyldimethylsilyl-4-*O*-(2-chlorophenyl)-2,3-dideoxy-β-d-*erythro*-hex-2-enopyranoside (**5j**)

Yellowish oil, 10 mg, 8% yield; R*_f_* = 0.74 (hexane/dichloromethane, 1:1); ^1^H-NMR (600 MHz, CDCl_3_) δ 0.00, 0,04 (2s, 6H, CH_3_ each), 0.87 (s, 9H, 3 × CH_3_), 3.76 (dd, 1H, *J* = 10.6, 4.7, H-6), 3.99 (dd, 1H, *J* = 10.6, 7.6, H-6′), 4.25–4.29 (m, 1H, H-5), 4.90 (dd ≈ t, 1H, *J* = 3.8, 3.8, H-4), 5.87 (s, 1H, H-1), 6.20 (ddd, 1H, *J* = 10.2, 2.3, 1.0, H-2), 6.29 (ddd, 1H, *J* = 10.2, 3.8, 0.9, H-3), 6.91–7.02 (m, 2H, H_ar_), 7.16–7.24 (m, 3H, H_ar_), 7.30–7.40 (m, 3H, H_ar_); ^13^C-NMR (150 MHz, CDCl_3_) δ −5.4, −5.3 (2 × CH_3_), 18.3 (Cq), 25.9 (3 × CH_3_), 62.9 (C-6), 68.9 (C-4), 76.2 (C-5), 93.4 (C-1), 115.3, 116.2, 122.3, 122.5, 124.0, 124.1, 126.6, 127.0 (C_ar_), 127.8 (C-3), 128.6 (C-2), 130.4, 130.4, 152.5, 153.4 (C_ar_).

##### 2-Chlorophenyl 6-*O*-*tert*-Butyldimethylsilyl-2-*O*-(2-chlorophenyl)-3,4-dideoxy-β-d-erythro-hex-3-enopyranoside (**6j**)

Yellowish oil, 70 mg, 58% yield; R*_f_* = 0.74 (hexane/dichloromethane, 1:1); ^1^H-NMR (600 MHz, CDCl_3_) δ 0.00, 0.03 (2s, 6H, CH_3_ each), 0.87 (s, 9H, 3 × CH_3_), 3.62 (dd, 1H, *J* = 10.0, 6.8, H-6), 3.71 (dd, 1H, *J* = 10.0, 6.1, H-6′), 4.48–4.51 (m, 1H, H-5), 4.95–4.98 (m, 1H, H-2), 5.57 (d, 1H, *J* = 5.0, H-1), 6.06 (ddd ≈ dt, 1H, *J* = 10.3, 2.6, 2.6, H-4), 6.13 (ddd ≈ dt, 1H, *J* = 10.3, 1.5, 1.5, H-3), 6.93–6.97 (m, 2H, H_ar_), 7.17–7.23 (m, 3H, H_ar_), 7.25–7.29 (m, 1H, H_ar_), 7.32–7.37 (m, 2H, H_ar_); ^13^C-NMR (150 MHz, CDCl_3_) δ −5.5, −5.4 (2 × CH_3_), 18.9 (Cq), 25.9 (3 × CH_3_), 65.6 (C-6), 74.1 (C-2), 75.1 (C-5), 98.7 (C-1), 116.7, 117.8, 122.8, 123.1, 123.7 (C_ar_), 124.0 (C-4), 124.3, 127.9, 128.0, 130.4, 130.5 (C_ar_), 130.6 (C-3), 152.5, 153.8 (C_ar_).

##### 2-Chlorophenyl 6-*O*-*tert*-Butyldimethylsilyl-4-*O*-(2-chlorophenyl)-2,3-dideoxy-α-d-*erythro*-hex-2-enopyranoside (**7j**)

Yellowish oil, 9 mg, 8% yield; R*_f_* = 0.74 (hexane/dichloromethane, 1:1); ^1^H-NMR (600 MHz, CDCl_3_) δ −0.03, 0.00 (2s, 6H, CH_3_ each), 0.83 (s, 9H, 3 × CH_3_), 3.91 (dd, 1H, *J* = 11.6, 1.7, H-6), 3.95 (dd, 1H, *J* = 11.6, 4.0, H-6′), 4.32 (ddd, 1H, *J* = 9.2, 4.0, 1.7, H-5), 5.02 (dd, 1H, *J* = 9.2, 1.3, H-4), 5.68 (s, 1H, H-1), 6.07 (ddd ≈ dt, 1H, *J* = 10.2, 2.3, 2.3, H-2), 6.27 (d, 1H, *J* = 10.2, H-3), 6.91–7.02 (m, 2H, H_ar_), 7.03–7.07 (m, 1H, H_ar_), 7.16–7.24 (m, 2H, H_ar_), 7.30–7.40 (m, 3H, H_ar_); ^13^C-NMR (150 MHz, CDCl_3_) δ −5.4, −5.3 (2 × CH_3_), 18.5 (Cq), 26.0 (3 × CH_3_), 62.2 (C-6), 69.8 (C-4), 71.6 (C-5), 95.0 (C-1), 117.4, 119.3, 123.1, 123.6, 124.3, 125.0, 127.8, 127.9 (C_ar_), 128.6 (C-3), 130.8 (C-2), 130.9, 131.0, 153.4, 153.5 (C_ar_).

Elemental analysis for the mixture of isomers: C_24_H_30_Cl_2_O_4_Si (481.49 g/mol) calculated: C% 59.87, H% 6.28; found: C% 59.96, H% 6.19.

#### 3.2.11. Reaction of Dicarbonate **3** with 1,2:5,6-Di-*O*-isopropylidene-α-d-glucofuranose (**4k**)

##### 1,2:5,6-Di-*O*-isopropylidene-α-d-glucofuranos-3-yl 6-*O*-*tert*-butyldimethylsilyl-4-*O*-(1,2:5,6-di-*O*-isopropylidene-α-d-glucofuranos-3-yl)-2,3-dideoxy-β-d-*erythro*-hex-2-enopyranoside (**5k**)

Yellowish oil, 120 mg, 64% yield; R*_f_* = 0.42 (hexane/ethyl acetate, 4:1); [α]_D_^20^ = −56.6 (*c* 1.4, CHCl_3_); ^1^H-NMR (600 MHz, CDCl_3_) δ 0.09, 0.10 (2s, 6H, CH_3_ each), 0.92 (s, 9H, 3 × CH_3_), 1.30, 1.32, 1.34, 1.35, 1.42, 1.42, 1.49, 1.49 (8s, 24H, CH_3_ each), 3.59–3.62 (m, 1H, H-5), 3.83 (dd, 1H, *J* = 11.3, 3.1, H-6), 3.97 (dd, 1H, *J* = 11.3, 5.5, H-6), 3.98–4.10 (m, 5H, H-4″, H-6′, H-6′, H-6″, H-6″), 4.13 (d, 1H, *J* = 3.1, H-4′), 4.19 (dd, 1H, *J* = 6.8, 3.1, H-3′), 4.21–4.24 (m, 1H, H-4), 4.25–4.32 (m, 3H, H-3″, H-5′, H-5″), 4.50 (d, 1H, *J* = 3.7, H-2″), 4.64 (d, 1H, *J* = 3.6, H-2′), 5.34 (d, 1H, *J* = 1.2, H-1), 5.79 (d, 1H, *J* = 10.3, H-2), 5.87–5.89 (m, 2H, H-1′, H-1″), 6.04 (d, 1H, *J* = 10.3, H-3); ^13^C-NMR (150 MHz, CDCl_3_) δ −5.2, −5.1 (2 × CH_3_), 18.5 (Cq), 26.1 (3 × CH_3_), 25.6, 25.7, 26.4, 26.6, 26.9, 26.9, 27.0, 27.0 (8 × CH_3_), 62.3 (C-6), 66.9 (C-6″), 67.7 (C-6′), 69.8 (C-4), 72.2 (C-5″), 73.0 (C-5′), 77.1 (C-3″), 78.1 (C-5), 80.6 (C-3′), 81.3 (C-4″), 82.1 (C-4′), 83.7 (C-2″), 83.9 (C-2′), 95.8 (C-1), 105.3 (C-1″), 105.2 (C-1′), 108.9, 109.2, 111.9, 112.1 (4 × Cq), 129.0 (C-3), 132.3 (C-2).

Elemental analysis: C_36_H_60_O_14_Si (744.95 g/mol) calculated: C% 58.04, H% 8.12; found: C% 58.08, H% 8.08.

##### 1,2:5,6-Di-*O*-isopropylidene-α-d-glucofuranos-3-yl 6-*O*-*tert*-butyldimethylsilyl-3-*O*-(1,2:5,6-di-*O*-isopropylidene-α-d-glucofuranos-3-yl)-3,4-dideoxy-β-d-*erythro*-hex-3-enopyranoside (**6k**)

Yellowish solid, 41 mg, 22% yield; R*_f_* = 0.36 (hexane/ethyl acetate, 4:1); m.p. = 55.7–56.9 °C; [α]_D_^20^ = +23.3 (*c* 1.3, CHCl_3_); ^1^H-NMR (600 MHz, CDCl_3_) δ 0.06 (s, 6H, 2 × CH_3_), 0.88 (s, 9H, 3 × CH_3_), 1.29, 1.30, 1.33, 1.35, 1.41, 1.44, 1.48, 1.50 (8s, 24H, CH_3_ each), 3.57 (dd, 1H, *J* = 10.3, 6.0, H-6), 3.69 (dd, 1H, *J* = 10.3, 5.6, H-6), 3.95 (dd, 1H, *J* = 8.6, 2.8, H-6′), 3.96–3.99 (m, 1H, H-5″), 4.01 (dd, 1H, *J* = 8.6, 2.9, H-6′), 4.03–4.12 (m, 3H, H-4″, 2 × H-6″), 4.17 (d, 1H, *J* = 2.9, H-4′), 4.19–4.23 (m, 2H, H-2, H-5′), 4.38 (d, 1H, *J* = 2.5, H-3″), 4.40–4.44 (m, 2H, H-5, H-3′), 4.50–4.54 (m, 2H, H-1, H-2″), 4.56 (d, 1H, *J* = 3.8, H-2′), 5.74 (ddd ≈ dt, 1H, *J* = 10.2, 1.9, 1.9, H-4), 5.81 (s, 1H, H-3), 5.83 (d, 1H, *J* = 3.5, H-1″), 6.00 (d, 1H, *J* = 3.8, H-1′); ^13^C-NMR (150 MHz, CDCl_3_) δ −5.2 (2 × CH_3_), 18.4 (Cq), 25.9 (3 × CH_3_), 25.5, 25.6, 26.3, 26.3, 26.7, 26.8, 27.0, 27.0 (8 × CH_3_), 65.3 (C-6), 65.9 (C-6″), 67.9 (C-6′), 72.7 (C-2), 73.8 (C-5), 75.9 (C-5″), 76.1 (C-5′), 79.5 (C-3″), 80.4 (C-3′), 81.6 (C-4″), 82.8 (C-2″), 83.2 (C-4′), 84.34 (C-2′), 100.3 (C-1), 105.3 (C-1″), 105.4 (C-1′), 108.5, 109.3, 112.0, 112.1 (4 × Cq), 126.3 (C-4), 129.3 (C-3).

Elemental analysis: C_36_H_60_O_14_Si (744.95 g/mol) calculated: C% 58.04, H% 8.12; found: C% 57.96, H% 7.98.

#### 3.2.12. Reaction of Dicarbonate **3** with 2,3:5,6-Di-*O*-isopropylidene-d-mannofuranose (**4l**)

##### 2,3:5,6-Di-*O*-isopropylidene-α-d-mannofuranosyl 6-*O*-*tert*-butyldimethylsilyl-4-*O*-(2,3:5,6-di-*O*-isopropylidene-α-d-mannofuranosyl)-2,3-dideoxy-β-d-*erythro*-hex-2-enopyranoside (**5l**)

Colorless solid, 123 mg, 66% yield; R*_f_* = 0.39 (hexane/ethyl acetate, 4:1); m.p. = 116.8–117.9 °C; [α]_D_^20^ = −4.8 (*c* 1.1, CHCl_3_); ^1^H-NMR (600 MHz, CDCl_3_) δ 0.06, 0.07 (2s, 6H, CH_3_ each), 0.89 (s, 9H, 3 × CH_3_), 1.31, 1.31, 1.37, 1.37, 1.42, 1.43, 1.45, 1.45 (8s, 24H, CH_3_ each), 3.70–3.80 (m, 3H, H-5, 2 × H-6), 3.94 (dd, 1H, *J* = 7.9, 3.6, H-4″), 3.98 (dd, 1H, *J* = 8.6, 4.8, H-6″), 4.02–4.06 (m, 2H, H-4′, H-6′), 4.08–4.12 (m, 2H, H-6′, H-6″), 4.16 (dd, 1H, *J* = 4.9, 3.7, H-4), 4.34–4.40 (m, 2H, H-5′, H-5″), 4.59 (d, 1H, *J* = 5.9, H-2″), 4.62 (d, 1H, *J* = 5.9, H-2′), 4.77–4.81 (m, 2H, H-3′, H-3″), 5.15 (s, 1H, H-1″), 5.25–5.28 (m, 2H, H-1, H-1′), 5.86 (ddd ≈ dt, 1H, *J* = 10.3, 1.2, 1.2, H-2), 6.07 (ddd, 1H, *J* = 10.3, 3.4, 1.6, H-3); ^13^C-NMR (150 MHz, CDCl_3_) −5.2, −5.0 (2 × CH_3_), 18.5 (Cq), 26.1 (3 × CH_3_), 24.7, 24.8, 25.4, 25.4, 26.1, 26.1, 27.0, 27.1 (8 × CH_3_), 63.1 (C-6), 65.9 (C-4), 67.2 (C-6″), 67.2 (C-6′), 73.1 (C-5″), 73.2 (C-5′), 77.3 (C-5), 79.6 (C-3″), 79.7 (C-3′), 81.2 (C-4″), 81.3 (C-4′), 85.6 (C-2″), 85.8 (C-2′), 94.2 (C-1), 104.6 (C-1″), 105.1 (C-1′), 109.4, 109.4, 112.8, 112.8 (4 × Cq), 127.2 (C-3), 129.4 (C-2).

Elemental analysis: C_36_H_60_O_14_Si (744.95 g/mol) calculated: C% 58.04, H% 8.12; found: C% 58.09, H% 8.07.

##### 2,3:5,6-Di-*O*-isopropylidene-α-d-mannofuranosyl 6-*O*-*tert*-butyldimethylsilyl-2-*O*-(2,3:5,6-di-*O*-isopropylidene-α-d-mannofuranosyl)-3,4-dideoxy-β-d-*erythro*-hex-3-enopyranoside (**6l**)

Colorless solid, 48 mg, 26% yield; R*_f_* = 0.28 (hexane/ethyl acetate, 4:1); m.p. = 143.5–144.8 °C; [α]_D_^20^ = +101.7 (*c* 1.5, CHCl_3_); ^1^H-NMR (600 MHz, CDCl_3_) δ 0.05 (s, 6H, 2 × CH_3_), 0.87 (s, 9H, 3 × CH_3_), 1.31, 1.32, 1.36, 1.37, 1.43, 1.44, 1.45, 1.46 (8s, 24H, CH_3_ each), 3.47 (dd, 1H, *J* = 9.8, 7.8, H-6), 3.76 (dd, 1H, *J* = 9.8, 4.9, H-6), 3.98–4.02 (m, 3H, H-4″, H-6′, H-6″), 4.05–4.11 (m, 3H, H-2, H-6′, H-6″), 4.13 (dd, 1H, *J* = 8.2, 3.5, H-4′), 4.19–4.23 (m, 1H, H-5), 4.36–4.41 (m, 2H, H-5′, H-5″), 4.59 (d, 1H, *J* = 5.9, H-2″), 4.63 (d, 1H, *J* = 6.8, H-1), 4.67 (d, 1H, *J* = 5.9, H-2′), 4.77 (dd, 1H, *J* = 5.9, 3.6, H-3″), 4.81 (dd, 1H, *J* = 5.9, 3.6, H-3′), 5.21 (s, 1H, H-1″), 5.25 (s, 1H, H-1′), 5.62 (ddd ≈ dt, 1H, *J* = 10.2, 2.2, 2.2, H-4), 5.93 (ddd ≈ dt, 1H, *J* = 10.2, 1.7, 1.7, H-3); ^13^C-NMR (150 MHz, CDCl_3_) δ −5.2 (2 × CH_3_), 18.4 (Cq), 26.0 (3 × CH_3_), 24.6, 24.7, 25.3, 25.4, 26.0, 26.1, 27.0, 27.1 (8 × CH_3_), 65.3 (C-6), 67.0 (C-6″), 67.1 (C-6′), 72.9 (C-2), 73.1 (C-5″), 73.3 (C-5′), 75.3 (C-5), 79.6 (C-3″), 79.56 (C-3′), 80.6 (C-4″), 81.2 (C-4′), 85.2 (C-2″), 85.23 (C-2′), 99.6 (C-1), 106.6 (C-1″), 107.13 (C-1′), 109.3, 109.5, 112.8, 112.8 (4 × Cq), 126.4 (C-4), 130.1 (C-3).

Elemental analysis: C_36_H_60_O_14_Si (744.95 g/mol) calculated: C% 58.04, H% 8.12; found: C% 57.98, H% 8.06.

## 4. Conclusions

In conclusion, a homogeneous Pd(0)-catalysed aryloxylation of 6-*O*-*tert*-butyldimethylsilyl-3,4-di-*O*-isobutyloxycarbonyl-d-glucal was developed as an efficient and stereoselective approach to 2,3- and 3,4-unsaturated aryl *O*-glycosides. The reactions proceeded with high β-selectivity, whereas the regioselectivity was strongly influenced by the structure of the nucleophile and the reaction conditions.

Comparison with the previously reported 6-*O*-*tert*-butyldiphenylsilyl analogue demonstrated a pronounced effect of the silyl protecting group on the regioselectivity of the reaction. The TBDMS-protected donor generally favored the formation of 2,3-unsaturated products, whereas the TBDPS analogue exhibited lower regioselectivity. These differences can be attributed to the combined steric and conformational effects of the protecting group on the carbohydrate-derived η^3^-π-allylpalladium intermediate, together with the electronic properties of the nucleophile. The results further indicated that reduced nucleophilicity can prolong the lifetime of the palladium intermediate and promote competitive isomerization, leading to the formation of an additional α-anomer.

Optimization studies showed that the ligand, solvent, and temperature significantly affected both the yield and regioselectivity of the transformation. Bidentate phosphines, particularly dppb and dppe, favored the formation of 2,3-unsaturated products, whereas the monodentate ligand PPh_3_ resulted in lower yields and regioselectivity. Among the solvents examined, THF provided the most favorable balance between yield and regioselectivity under the optimized conditions. These findings are consistent with a reaction pathway involving competition between nucleophilic attack on the η^3^-π-allylpalladium intermediate and its isomerization. Consequently, the structure of the protecting group and nucleophile, together with the choice of ligand and solvent, collectively determines the reactivity and lifetime of this intermediate. Further kinetic or computational studies would be required to quantify the individual contributions of these factors.

The methodology was also extended to carbohydrate nucleophiles, demonstrating its potential for synthesis of more complex glycosidic structures. However, the reactivity of the carbohydrate acceptors was strongly dependent on their protecting-group pattern and hydroxyl-group environment. Monosaccharides bearing ether-type protecting groups and free secondary hydroxyl groups proved to be suitable substrates, whereas ester-protected sugars and substrates containing primary hydroxyl groups remained unreactive under the optimized conditions.

Overall, this study demonstrates the utility of Pd(0)-catalysed allylic substitution as a versatile platform for the stereoselective synthesis of unsaturated *O*-glycosides and oligosaccharide derivatives, while providing valuable insights into the influence of protecting groups, nucleophile structure, and reaction conditions on reaction outcome.

## Figures and Tables

**Table 1:** Effect of ligand and solvent on Pd(0)-catalysed aryloxylation reaction unsaturated carbohydrate **3** with phenol **4a**.

Entry	Ligand	Solvent	Temp. [°C]	5a:6a:7a [%] ^1^	Yield 5a + 6a [%] ^2^
1	dppb	THF	20	94:6:0	20
2	dppb	THF	60	86:14:0	79
3	dppe	THF	60	87:13:0	74
4	PPh_3_	THF	60	56:44:0	39
5	dppb	CH_3_CN	60	84:16:0	43
6	dppb	CH_2_Cl_2_	35	72:28:0	59
7	dppb	C_6_H_5_CH_3_	60	70:30:0	52

Reagents and conditions: [**3**]:[**4a**]:[Pd_2_(dba)_3_]:[ligand] = 20:60:1:2.2(4.4), 24 h. ^1^ Determined by ^1^H NMR spectroscopy. ^2^ Isolated products.

**Table 2:** Selected ^1^H and ^13^C NMR data for compounds **5a**–**j** and **6a**–**j** ^a^.

Phenol	2,3-Unsaturated Isomer 5				3,4-Unsaturated Isomer 6			
	δ H-1	δ C-1	δ H-4	δ C-4	δ H-1	δ C-1	δ H-2	δ C-2
**4a**	5.79	93.8	4.84	67.9	5.37	99.3	4.93	72.9
**4b**	5.81	93.1	4.85	67.0	5.42	98.3	4.85	71.8
**4c**	5.79	93.3	4.83	67.3	5.38	98.8	4.83	72.3
**4d**	5.83	92.9	4.86	67.1	5.45	98.3	4.88	71.8
**4e**	5.81	92.7	4.83	69.9	5.44	97.9	4.83	71.5
**4f**	5.74	-	4.54	-	-	-	-	-
**4g**	5.83	92.8	4.87	66.9	5.45	98.3	4.91	71.8
**4h**	6.01	92.8	5.07	66.9	5.68	98.3	5.13	71.7
**4i**	5.80	92.9	4.83	67.3	5.34	98.9	4.85	72.5
**4j**	5.87	93.4	4.90	68.9	5.57	98.5	4.97	74.0

^a^ Recorded in CDCl_3_ with TMS as a internal standard.

**Table 3:** Pd(0)-catalysed aryloxylation reaction unsaturated carbohydrate **3** with phenols **4a**–**j** according to Scheme 2.

Entry	Phenol	5:6:7 [%] ^1^	Yield 5 + 6 + 7 [%] ^2^	5:6 [%] ^3^	Yield 5 + 6 [%] ^3^
1	**4a**	86:14:0	79	72:28	78
2	**4b**	91:9:0	75	70:30	73
3	**4c**	90:10:0	87	27:73	55
4	**4d**	88:12:0	64	-	-
5	**4e**	85:15:0	78	-	-
6	**4f**	100:0:0	5	-	-
7	**4g**	87:13:0	80	46:54	68
8	**4h**	84:16:0	49	54:46	54
9	**4i**	59:30:11	90	79:21 ^4^	28
10	**4j**	11:78:11	74	-	-

Reagents and conditions: [**3**]:[**4**]:[Pd_2_(dba)_3_]:[ligand] = 20:60:1:2.2, 60 °C, 24 h. ^1^ Determined by ^1^H NMR spectroscopy. ^2^ Isolated products. ^3^ Results obtained for 6-*O*-*tert*-butyldiphenylsilyl-3,4-di-*O*-isobutyloxycarbonyl-d-glucal [36]. ^4^ 2,3-dideoxy-α-d-*erythro*-hex-2-enopyranoside was not analyzed.

## Data Availability

The original contributions presented in this study are included in the article. Further inquiries can be directed to the corresponding authors.

## Supplementary Materials

The following supporting information can be downloaded at: https://www.mdpi.com/article/10.3390/molecules31172965/s1, NMR spectra of the synthesized compounds (^1^H NMR spectra of compounds **2**, **3**, **5a**–**l**, **6a**–**l**, and **7i**–**j**; ^13^C NMR spectra of compounds **2, 3, 5a**–**e, 5g**–**l, 6a**–**e, 6g**–**l** and **7i**–**j**; ^1^H–^1^H COSY spectra of compounds **5a**–**c**, **5e**, **5g**–**i**, **5k**–**l**, **6a**–**c**, **6e**, **6g**–**i**, **6k**–**l**, **7i** and ^1^H–^13^C HMQC spectra of compounds **5a**–**c**, **5e, 5h**–**g, 5k**–**l, 6a**–**c, 6e, 6g**–**h, 6k**–**l**), Determination of the stereochemical configuration: representative examples (**5b**, **6b**, **5i**, **6i** and **7i**).
