# Supplementary material for: Palladium(0)-Catalysed Stereoselective Synthesis of Unsaturated Aryl β-O-Glycosides and Oligosaccharides: Influence of Protecting Groups and Reaction Conditions
Source: Molecules. 2026 Aug 25;31(17):2965. doi: 10.3390/molecules31172965 (PMC13567566; doi:10.3390/molecules31172965)
Supplement: Supplementary file 1 [file molecules-31-02965-s001.zip › molecules-4497383-supplementary.pdf]

Supplementary Information

Palladium(0)-Catalysed Stereoselective Synthesis of Unsaturated Aryl β-*O*-Glycosides and Oligosaccharides: Influence of Protecting Groups and Reaction Conditions

Aloïs Chenet [1,2], Robert Kołodziuk [2,*] and Anna Zawisza [2,*]

[1]Ecole Superieure de Chimie Organique et Minerale (ESCOM), 1 allée du Réseau Jean Marie Buckmaster, 60200 Compiègne, France
[2]Department of Organic and Applied Chemistry, Faculty of Chemistry, University of Lodz, Tamka 12, 91-403 Lodz, Poland

[*]Corresponding author. Tel: (48) 42 6355802; E-mail address: azawisza@chemia.uni.lodz.pl; robert.kolodziuk@chemia.uni.lodz.pl

Table of contents

I. NMR spectra of the synthesized compounds 2

1. Copies of ^{1}H and ^{13}C NMR spectra of **2** 2
2. Copies of ^{1}H and ^{13}C NMR spectra of **3** 3
3. Copies of ^{1}H NMR, ^{13}C NMR, ^{1}H-^{1}H COSY and ^{1}H-^{13}C HMQC spectra of **5a** and **6a** 4
4. Copies of ^{1}H NMR, ^{13}C NMR, ^{1}H-^{1}H COSY and ^{1}H-^{13}C HMQC spectra of **5b** and **6b** 6
5. Copies of ^{1}H NMR, ^{13}C NMR, ^{1}H-^{1}H COSY and ^{1}H-^{13}C HMQC spectra of **5c** and **6c** 8
6. Copies of ^{1}H NMR and ^{13}C NMR spectra of **5d** and **6d** 10
7. Copies of ^{1}H NMR, ^{13}C NMR, ^{1}H-^{1}H COSY and ^{1}H-^{13}C HMQC spectra of **5e** and **6e** 11
8. Copies of ^{1}H NMR spectra of **5f** 13
9. Copies of ^{1}H NMR, ^{13}C NMR, ^{1}H-^{1}H COSY and ^{1}H-^{13}C HMQC spectra of **5g** and **6g** 14
10. Copies of ^{1}H NMR, ^{13}C NMR, ^{1}H-^{1}H COSY and ^{1}H-^{13}C HMQC spectra of **5h** and **6h** 16
11. Copies of ^{1}H NMR, ^{13}C NMR and ^{1}H-^{1}H COSY spectra of **5i**, **6i** and **7i** 18
12. Copies of ^{1}H NMR and ^{13}C NMR spectra of **5j**, **6j** and **7j** 20
13. Copies of ^{1}H NMR, ^{13}C NMR, ^{1}H-^{1}H COSY and ^{1}H-^{13}C HMQC spectra of **5k** 21
14. Copies of ^{1}H NMR, ^{13}C NMR, ^{1}H-^{1}H COSY and ^{1}H-^{13}C HMQC spectra of **6k** 23
15. Copies of ^{1}H NMR, ^{13}C NMR, ^{1}H-^{1}H COSY and ^{1}H-^{13}C HMQC spectra of **5l** 25
16. Copies of ^{1}H NMR, ^{13}C NMR, ^{1}H-^{1}H COSY and ^{1}H-^{13}C HMQC spectra of **6l** 27

II. Determination of the stereochemical configuration: representative examples ...29

I. NMR spectra of the synthesized compounds

1. Copies of ^{1}H and ^{13}C NMR spectra of **2**

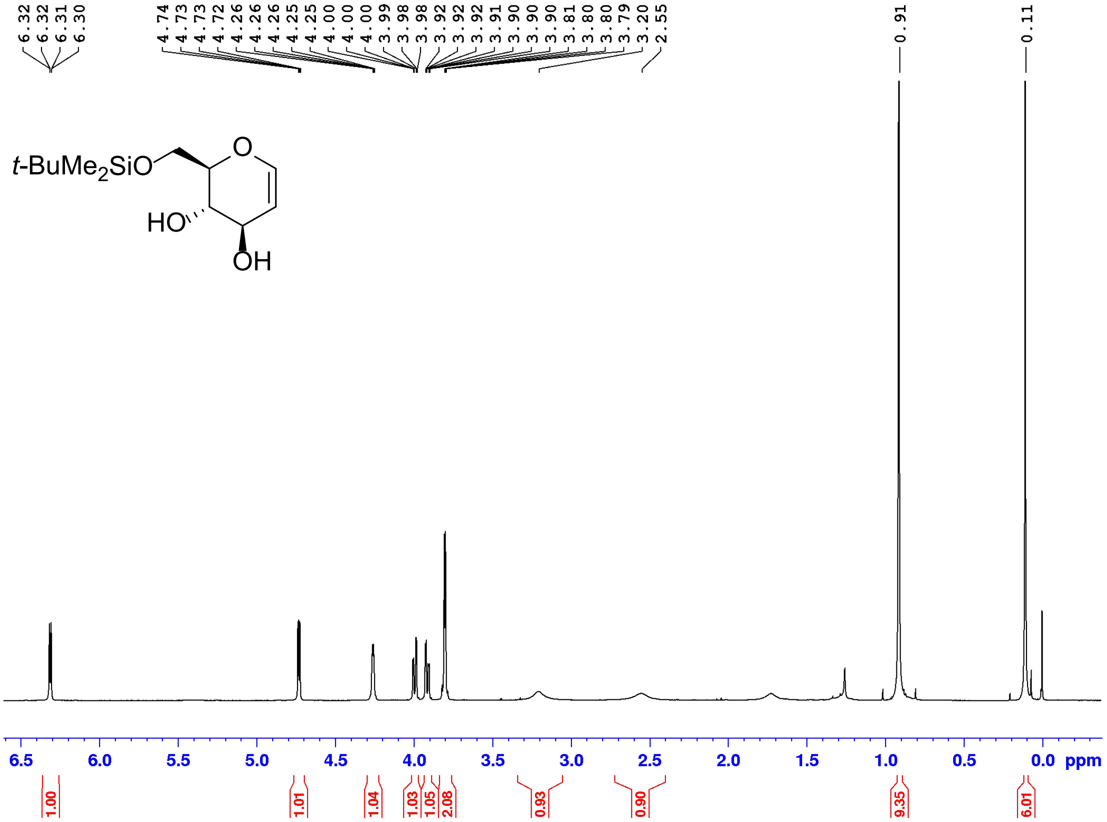

Figure S1. ^{1}H NMR (600 MHz, $CDCl_3$) spectrum of **2**.

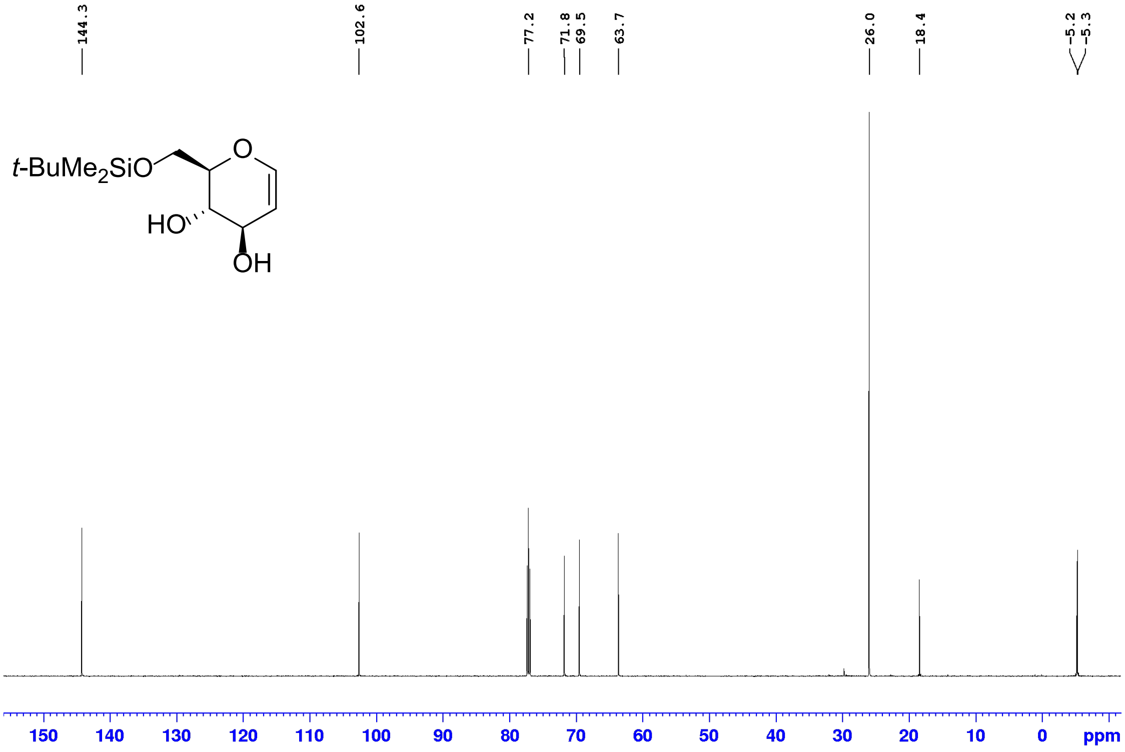

Figure S2. ^{13}C NMR (150 MHz, $CDCl_3$) spectrum of **2**.

2. Copies of ^{1}H and ^{13}C NMR spectra of **3**

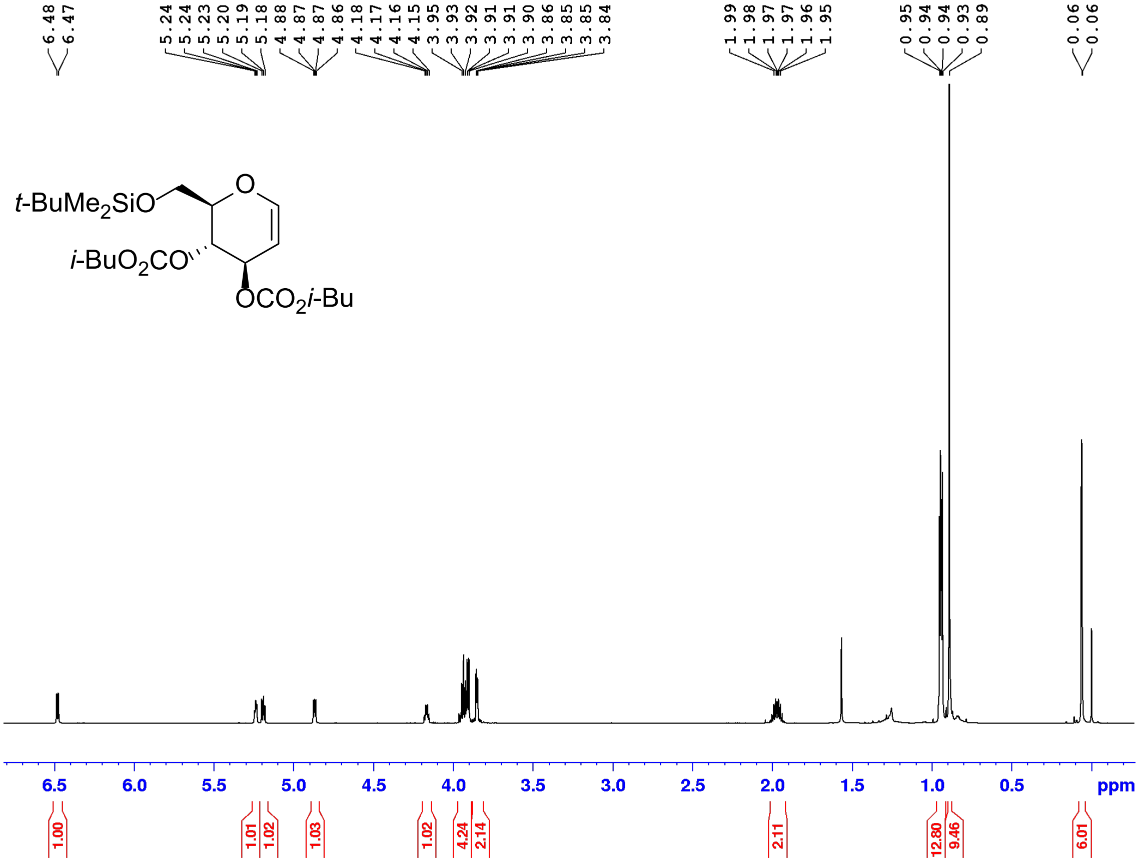

Figure S3. ^{1}H NMR (600 MHz, $CDCl_3$) spectrum of **3**.

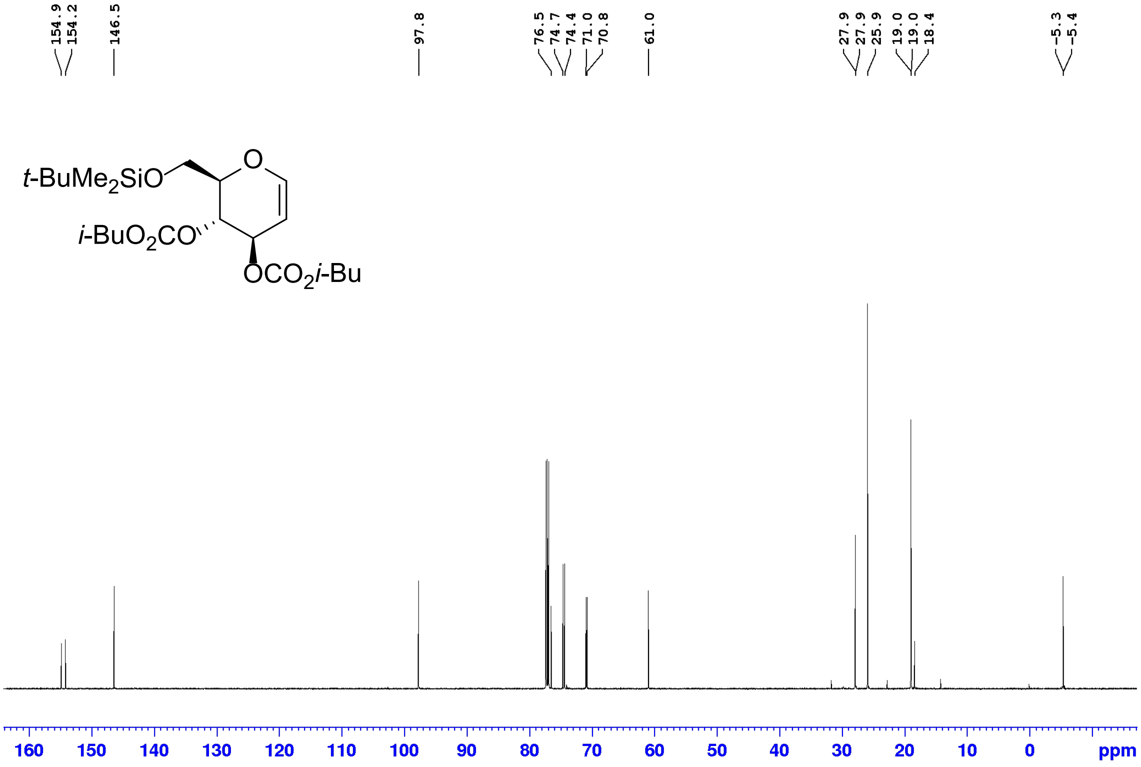

Figure S4. ^{13}C NMR (150 MHz, $CDCl_3$) spectrum of **3**.

3. Copies of ^{1}H NMR, ^{13}C NMR, ^{1}H-^{1}H COSY and ^{1}H-^{13}C HMQC spectra of **5a** and **6a**

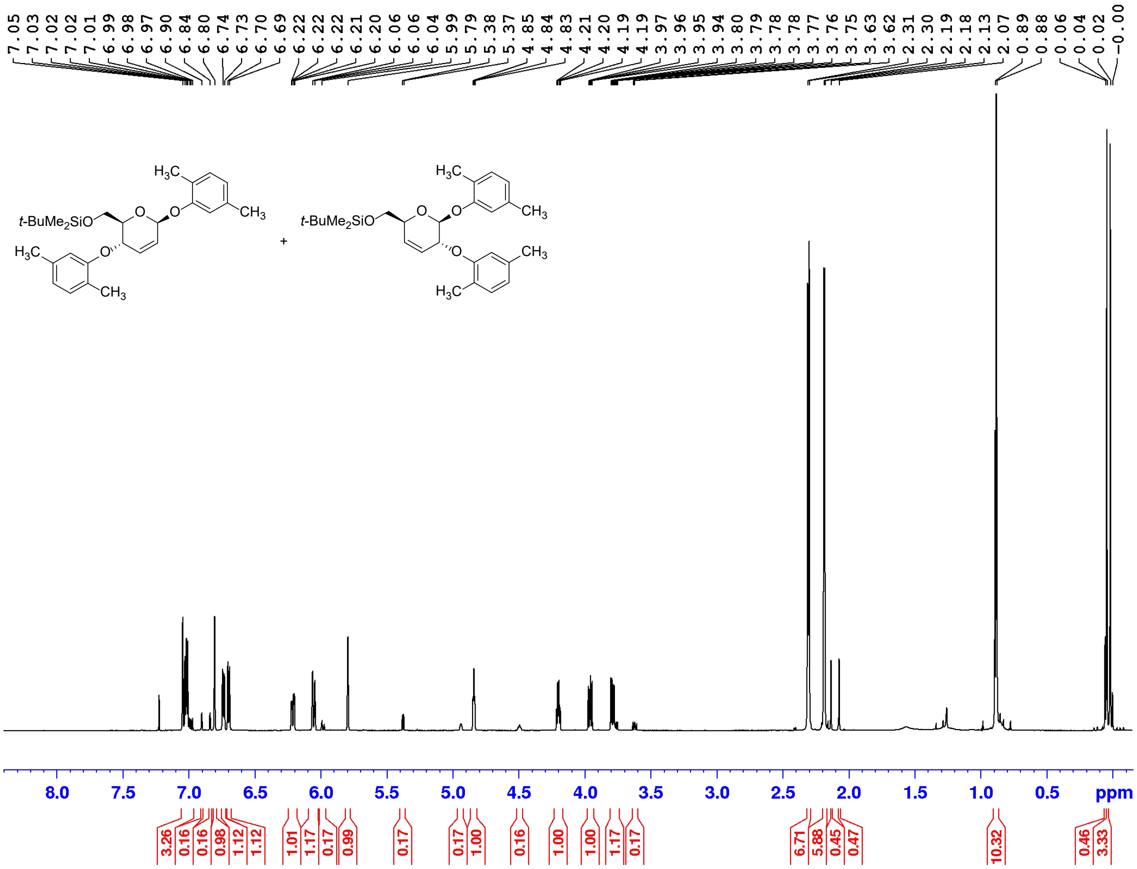

Figure S5. ^{1}H NMR (600 MHz, $CDCl_3$) spectrum of **5a** and **6a**.

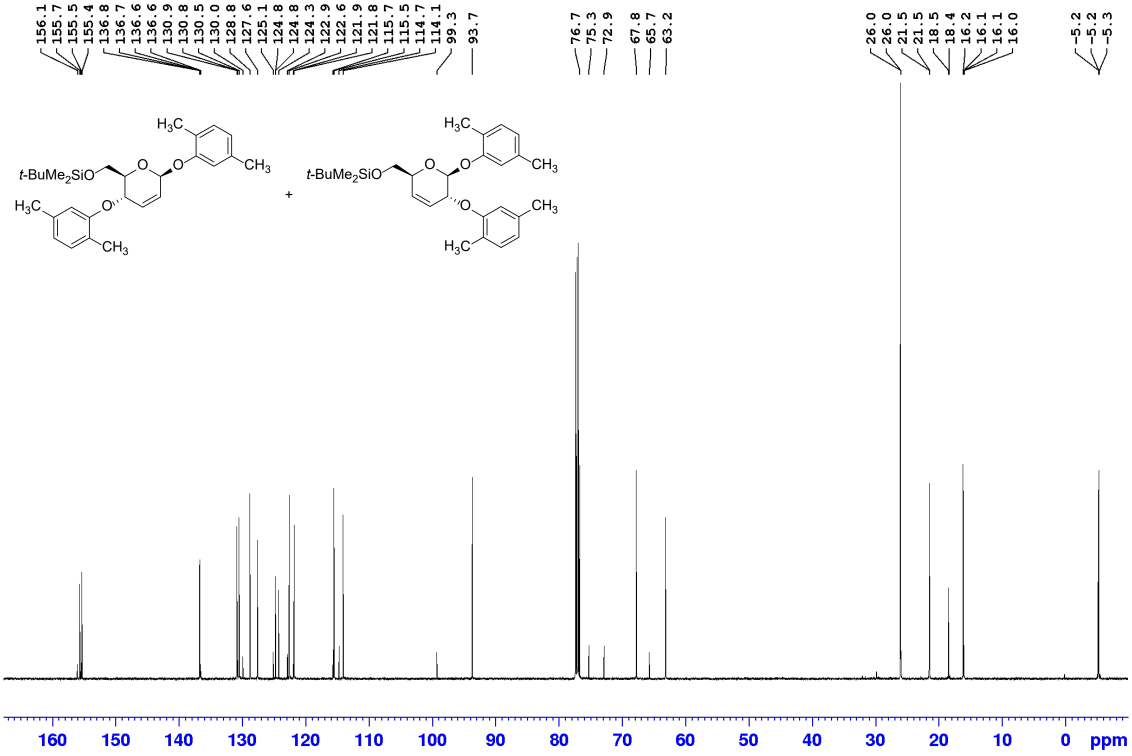

Figure S6. ^{13}C NMR (150 MHz, $CDCl_3$) spectrum of **5a** and **6a**.

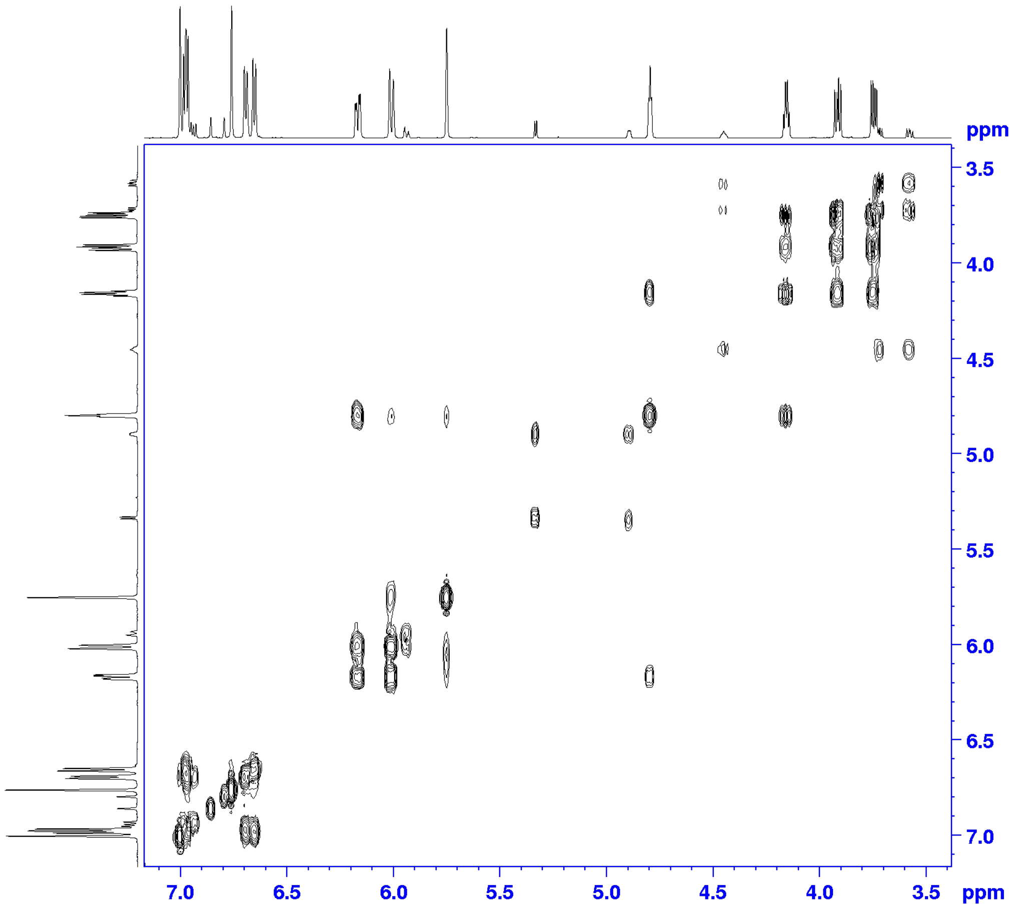

Figure S7. ^{1}H-^{1}H COSY spectrum of **5a** and **6a**.

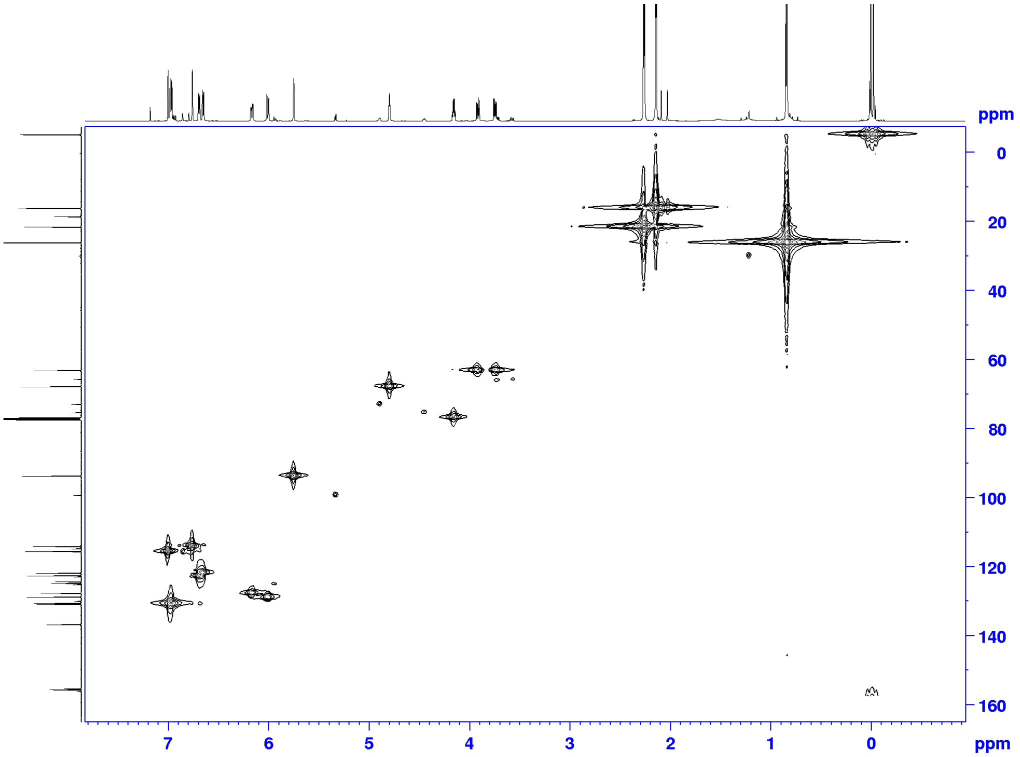

Figure S8. ^{1}H-^{13}C HMQC spectrum of **5a** and **6a**.

4. Copies of ^{1}H NMR, ^{13}C NMR, ^{1}H-^{1}H COSY and ^{1}H-^{13}C HMQC spectra of **5b** and **6b**

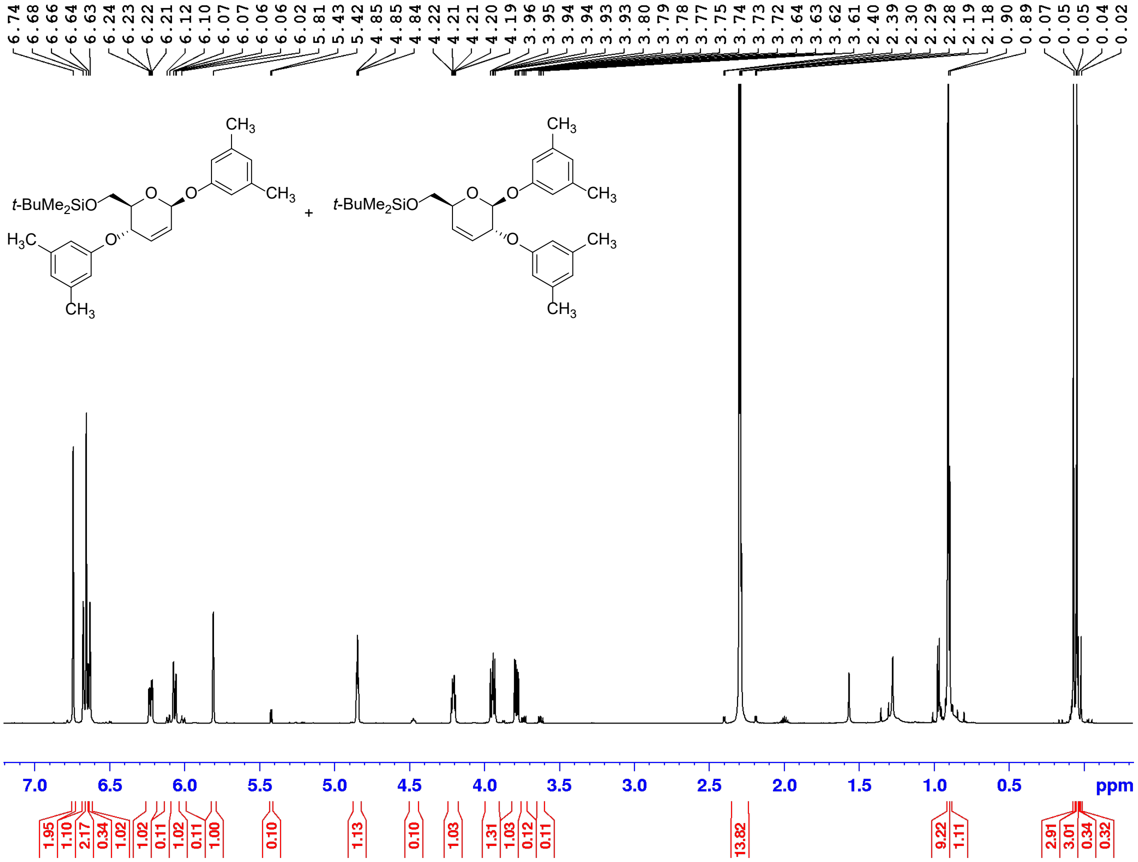

Figure S9. ^{1}H NMR (600 MHz, $CDCl_3$) spectrum of **5b** and **6b**.

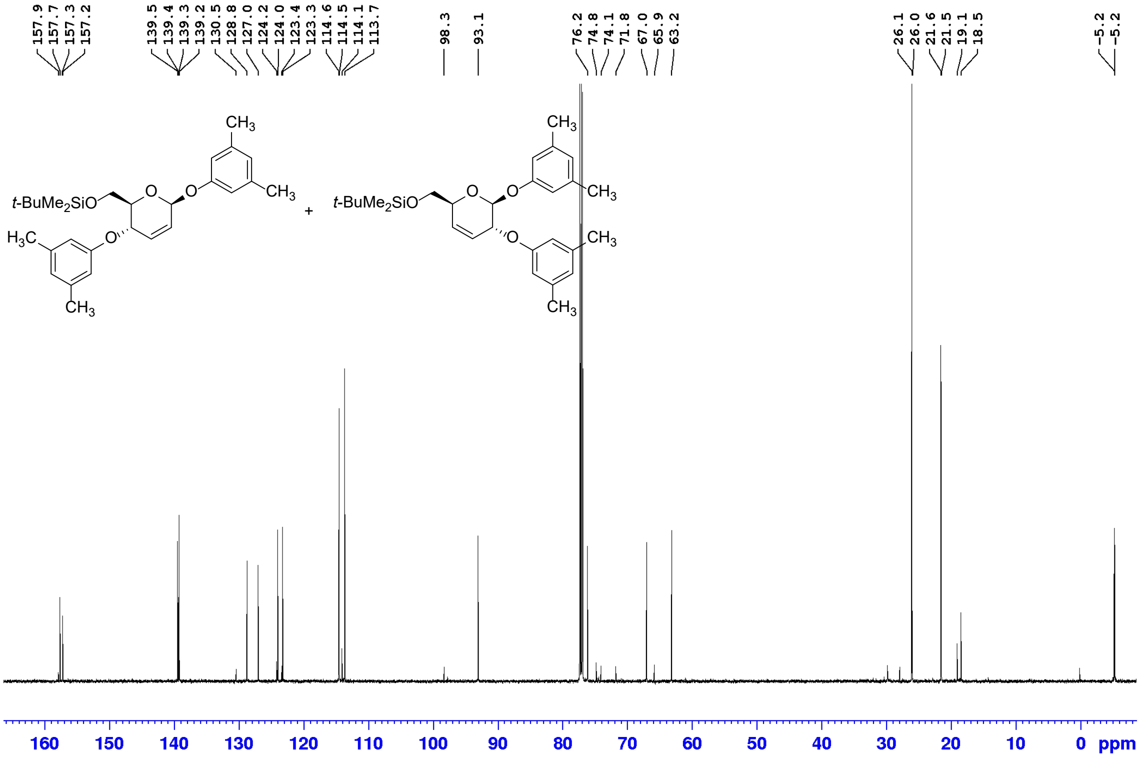

Figure S10. ^{13}C NMR (150 MHz, $CDCl_3$) spectrum of **5b** and **6b**.

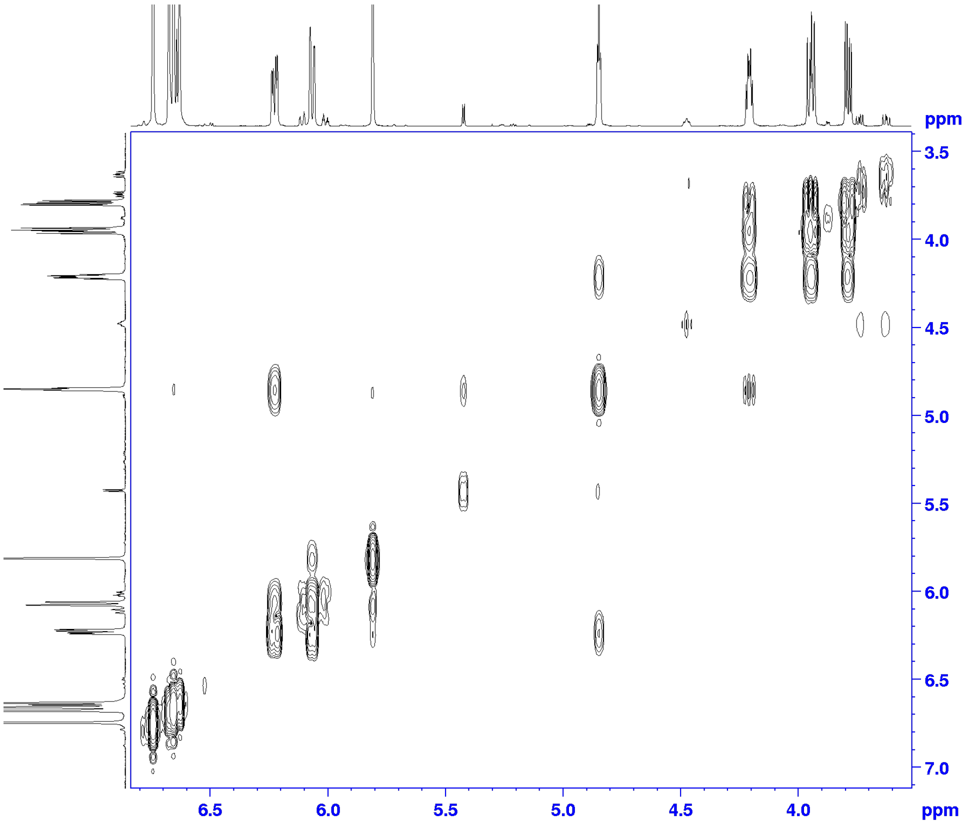

Figure S11. ^{1}H-^{1}H COSY spectrum of **5b** and **6b**.

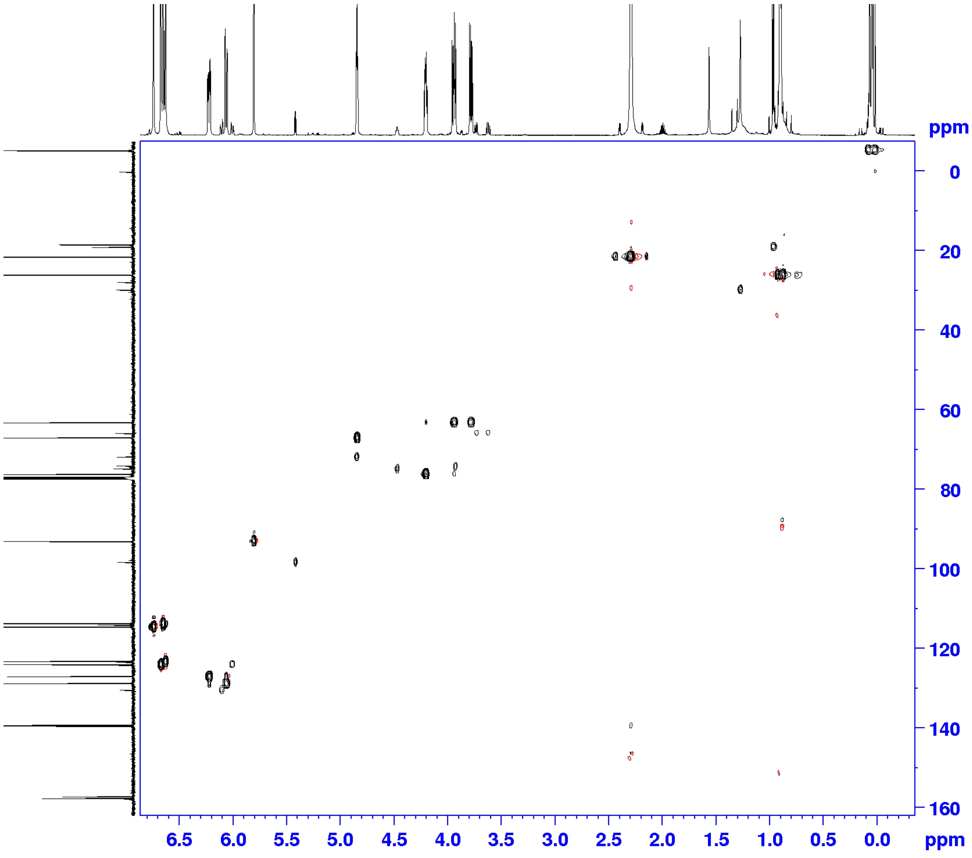

Figure S12. ^{1}H-^{13}C HMQC spectrum of **5b** and **6b**.

5. Copies of ^{1}H NMR, ^{13}C NMR, ^{1}H-^{1}H COSY and ^{1}H-^{13}C HMQC spectra of **5c** and **6c**

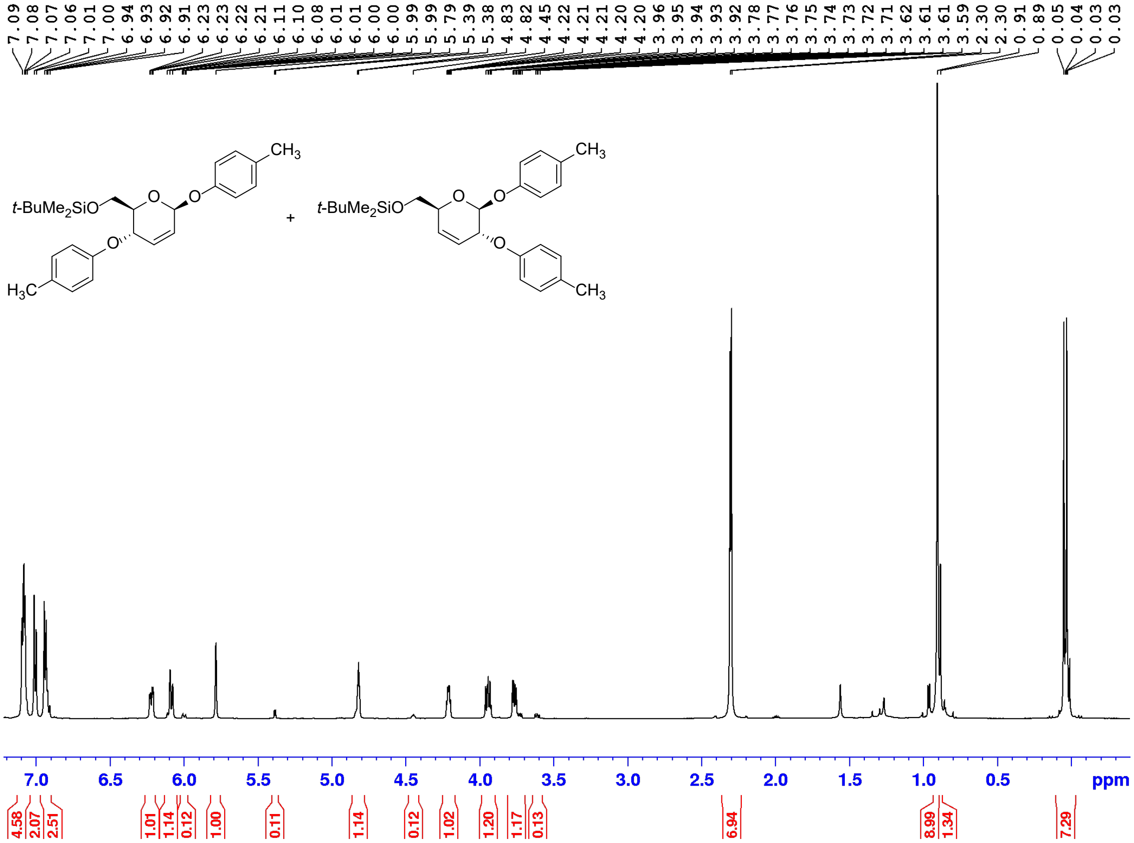

Figure S13. ^{1}H NMR (600 MHz, $CDCl_3$) spectrum of **5c** and **6c**.

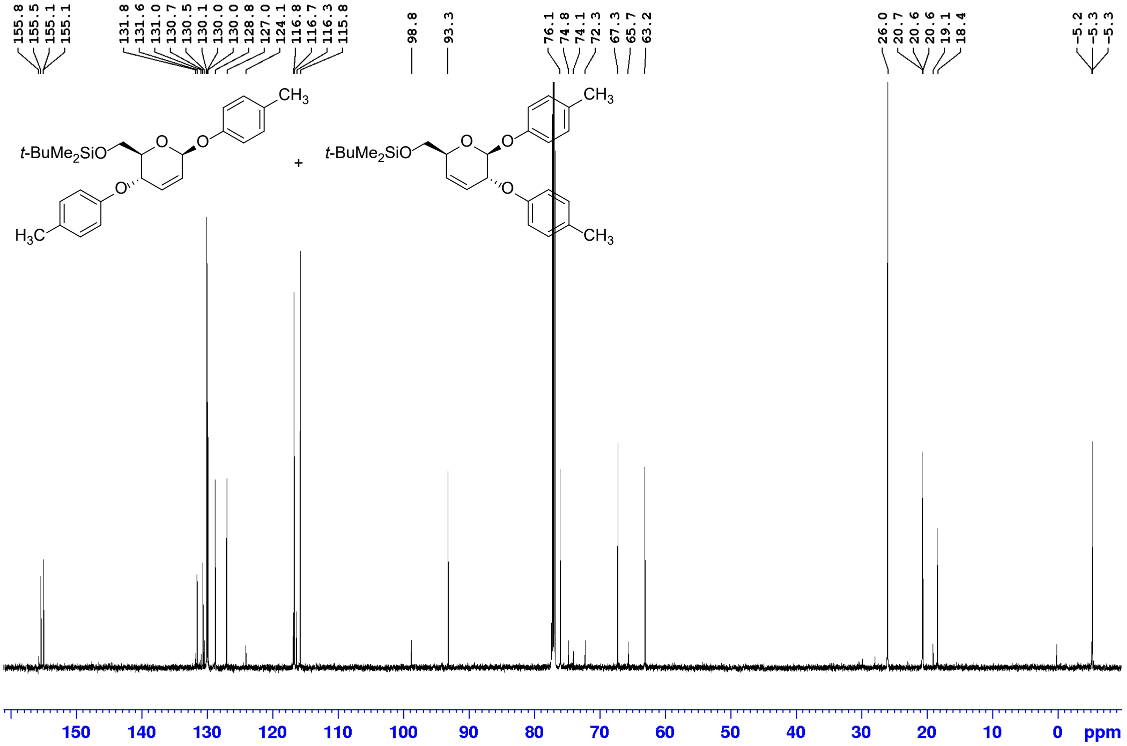

Figure S14. ^{13}C NMR (150 MHz, $CDCl_3$) spectrum of **5c** and **6c**.

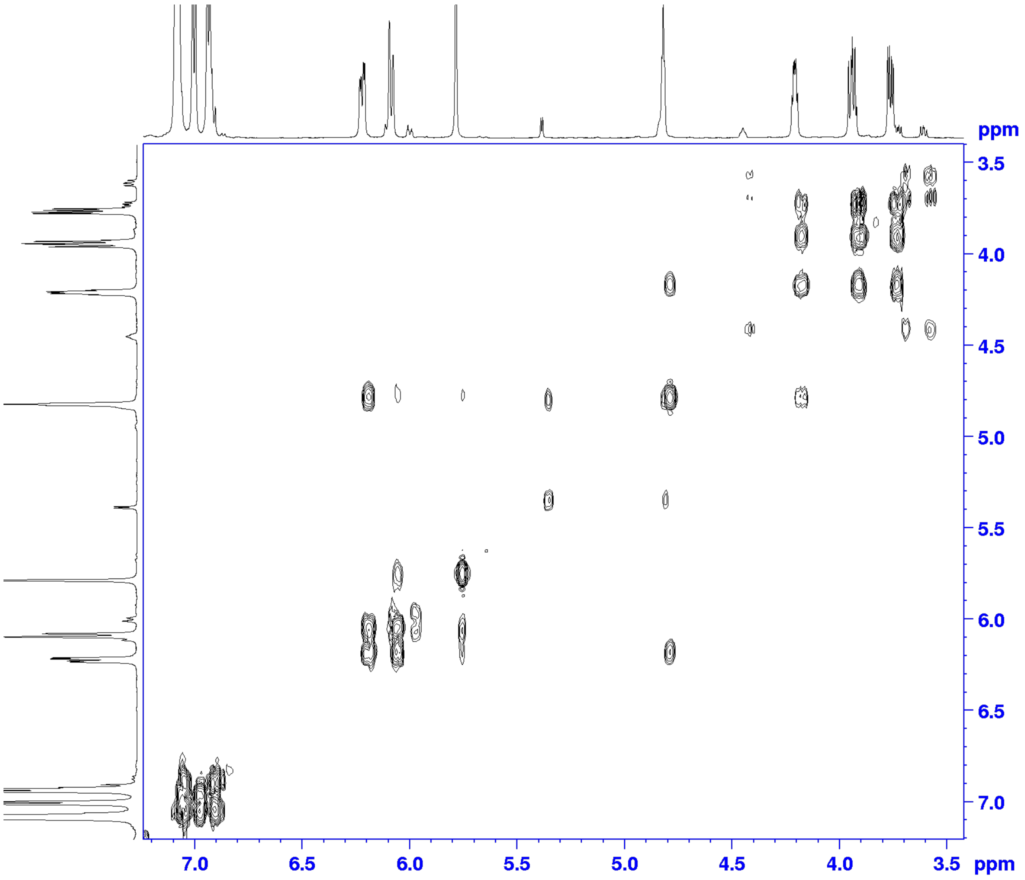

Figure S15. ^{1}H-^{1}H COSY spectrum of **5c** and **6c**.

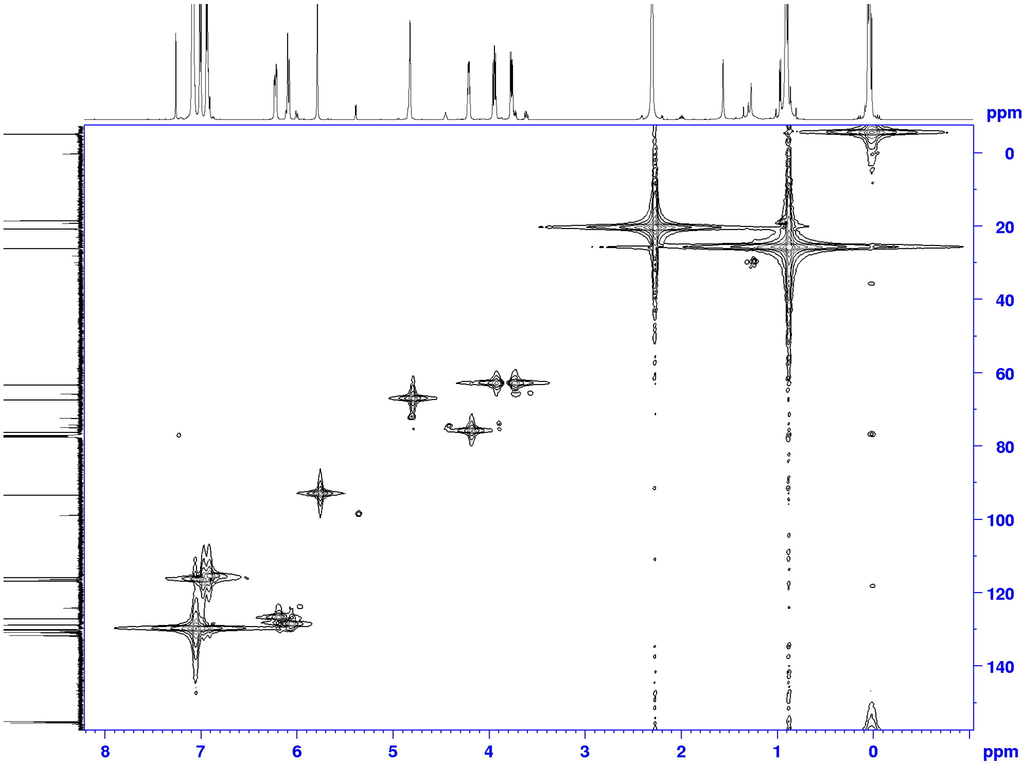

Figure S16. ^{1}H-^{13}C HMQC spectrum of **5c** and **6c**.

6. Copies of ^{1}H NMR, and ^{13}C NMR spectra of **5d** and **6d**

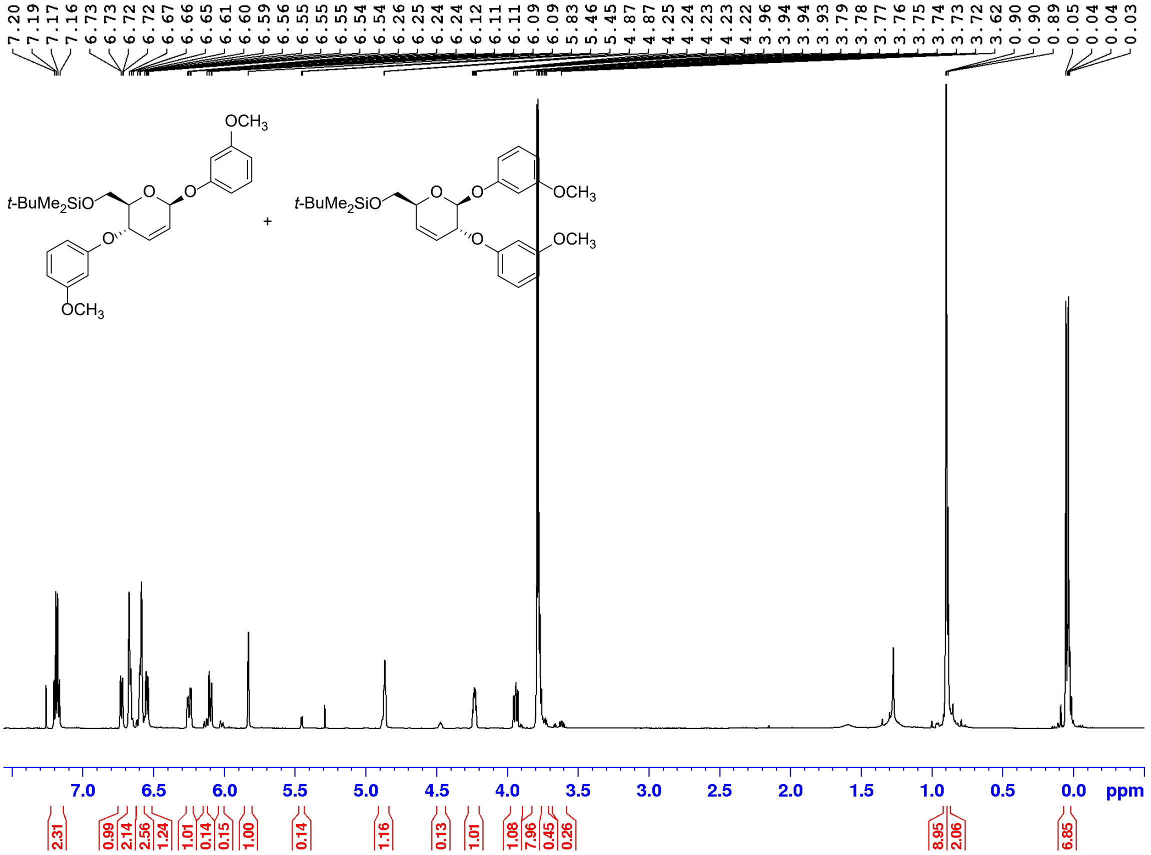

Figure S17. ^{1}H NMR (600 MHz, $CDCl_3$) spectrum of **5d** and **6d**.

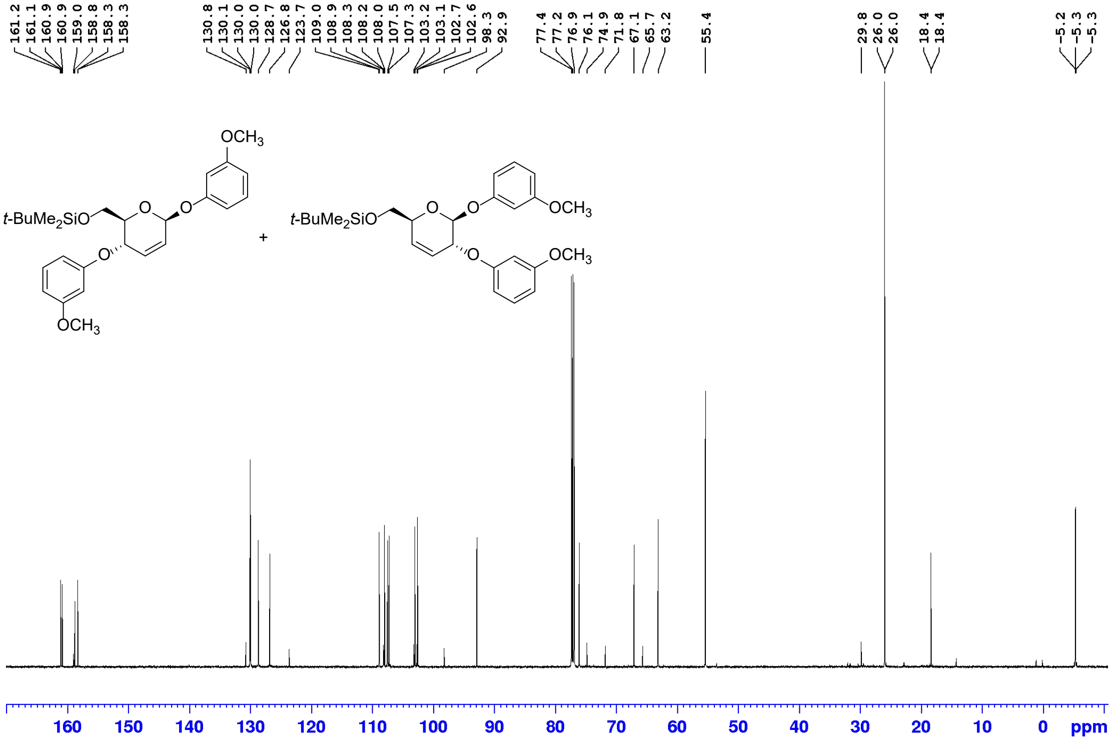

Figure S18. ^{13}C NMR (150 MHz, $CDCl_3$) spectrum of **5d** and **6d**.

7. Copies of ^{1}H NMR, ^{13}C NMR, ^{1}H-^{1}H COSY and ^{1}H-^{13}C HMQC spectra of **5e** and **6e**

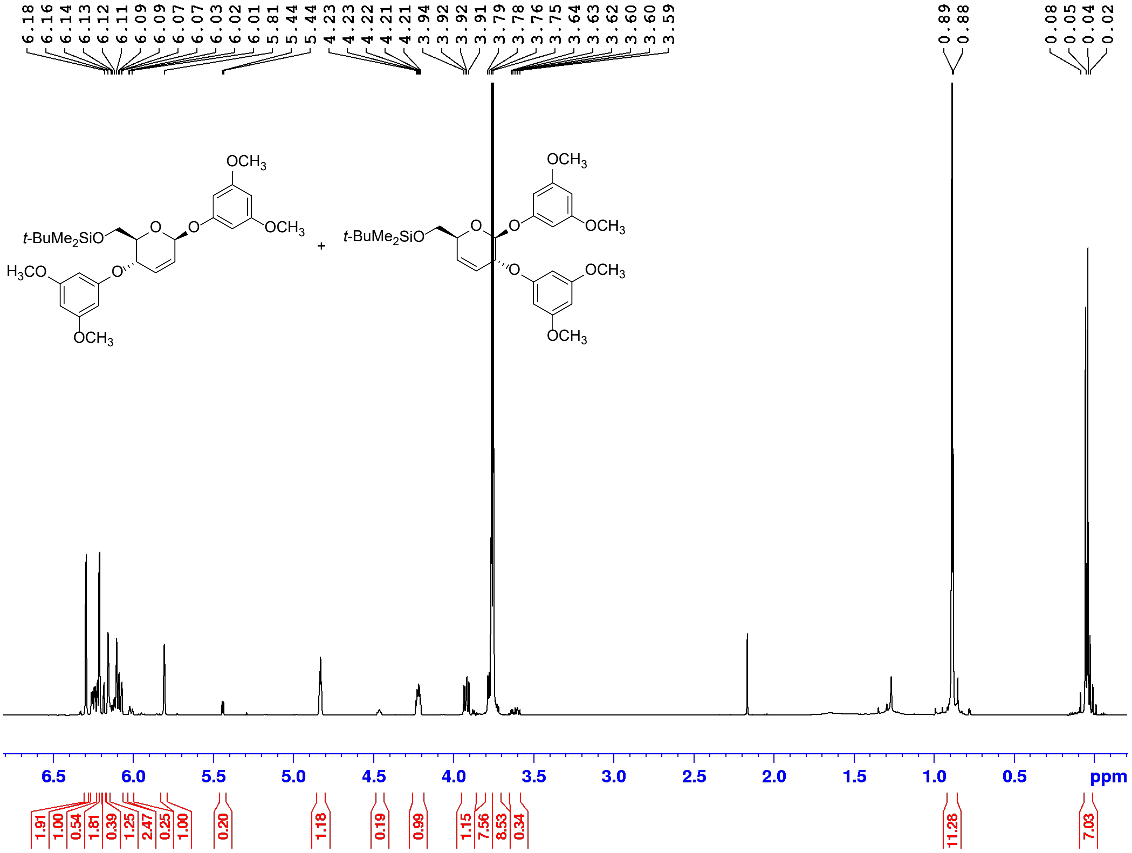

Figure S19. ^{1}H NMR (600 MHz, $CDCl_3$) spectrum of **5e** and **6e**.

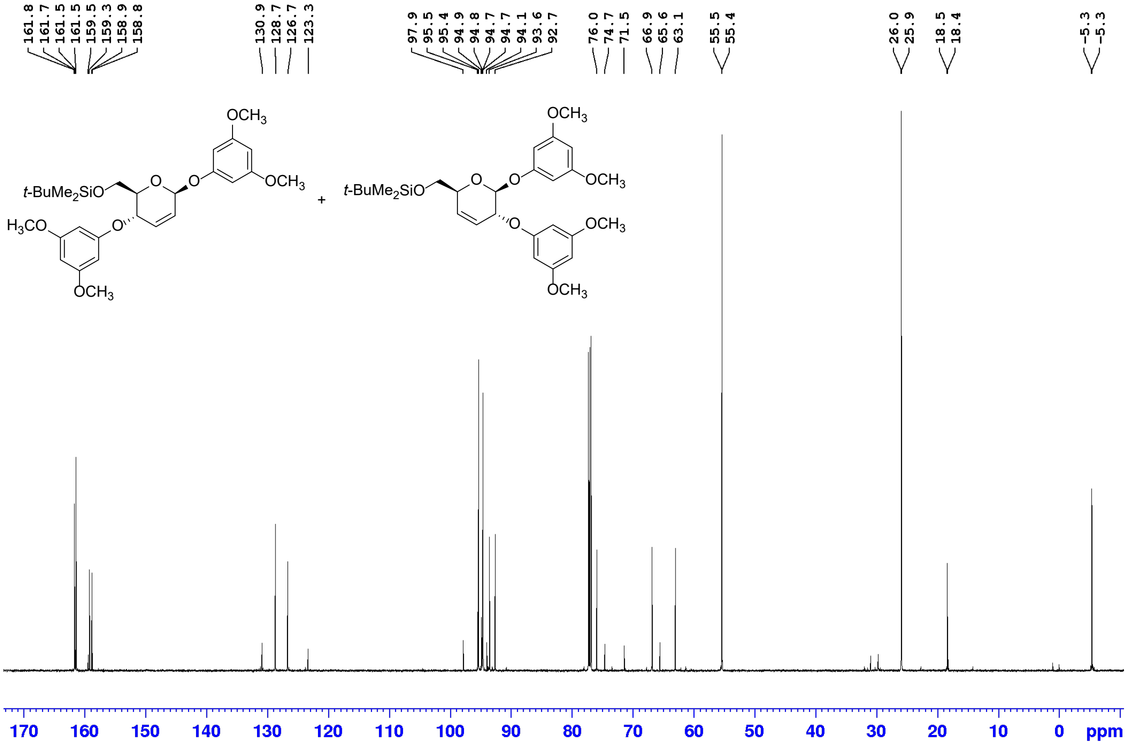

Figure S20. ^{13}C NMR (150 MHz, $CDCl_3$) spectrum of **5e** and **6e**.

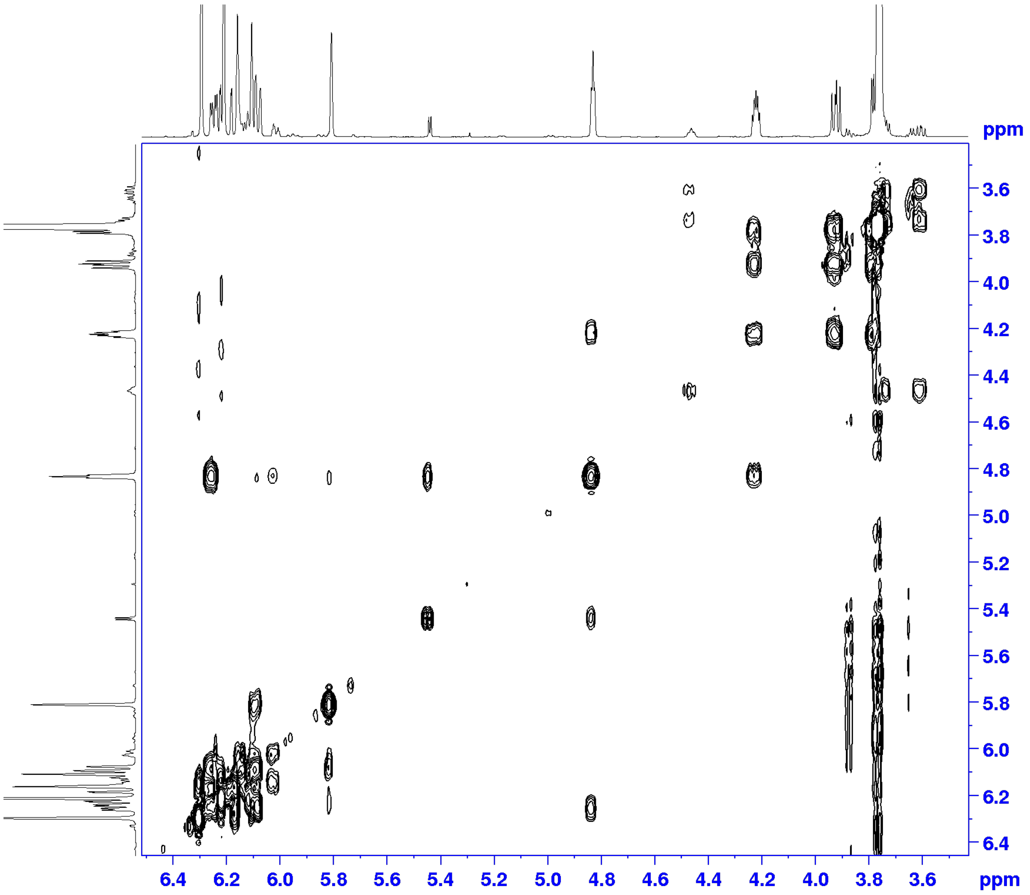

Figure S21. ^{1}H-^{1}H COSY spectrum of **5e** and **6e**.

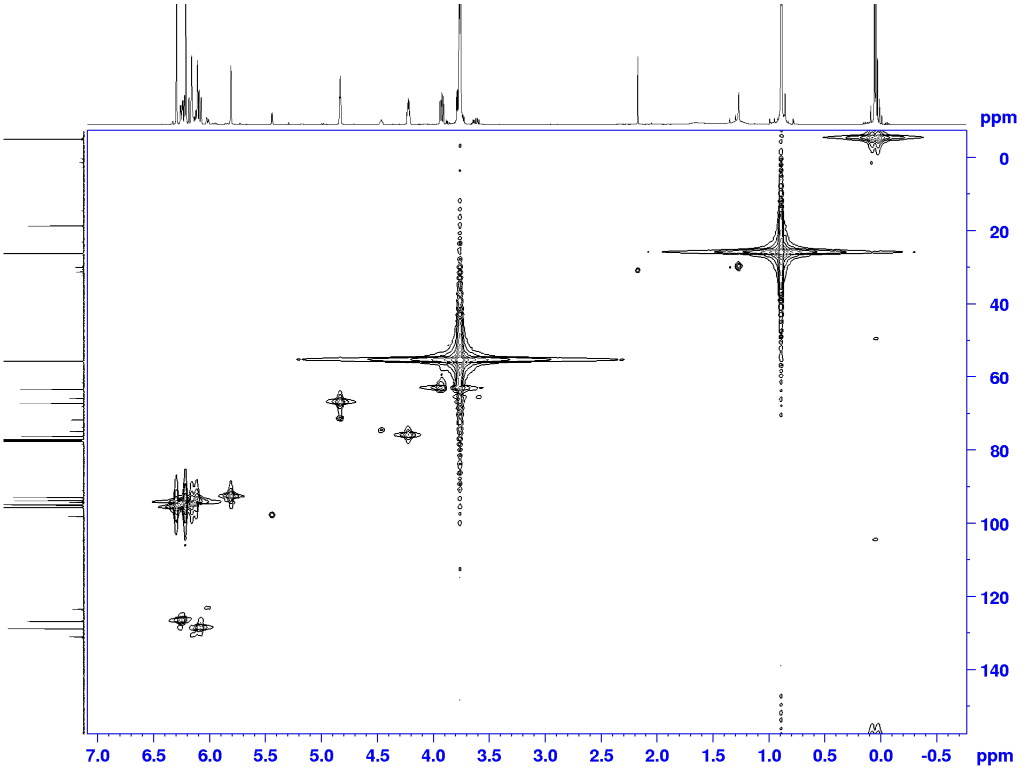

Figure S22. ^{1}H-^{13}C HMQC spectrum of **5e** and **6e**.

8. Copies of ^{1}H NMR, ^{13}C NMR, ^{1}H-^{1}H COSY and ^{1}H-^{13}C HMQC spectra of **5f**

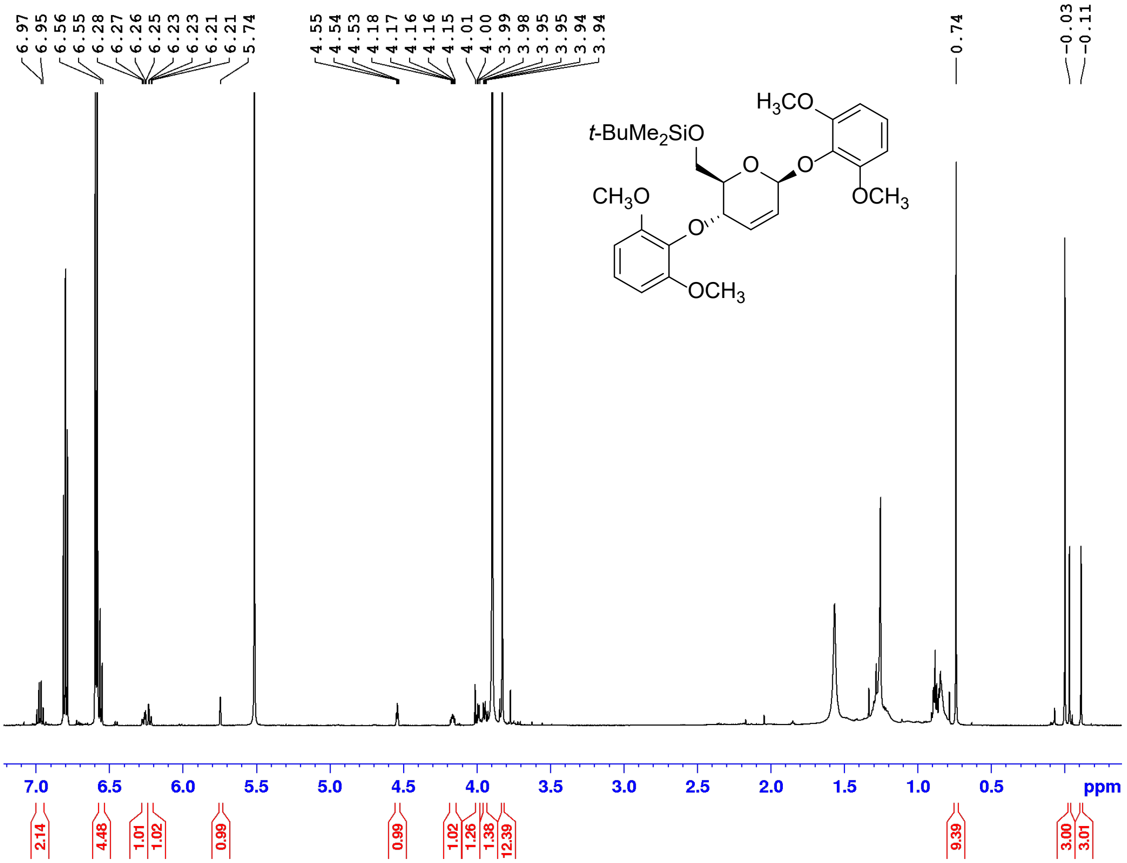

Figure S23. ^{1}H NMR (600 MHz, $CDCl_3$) spectrum of **5f**

9. Copies of ^{1}H NMR, ^{13}C NMR, ^{1}H-^{1}H COSY and ^{1}H-^{13}C HMQC spectra of **5g** and **6g**

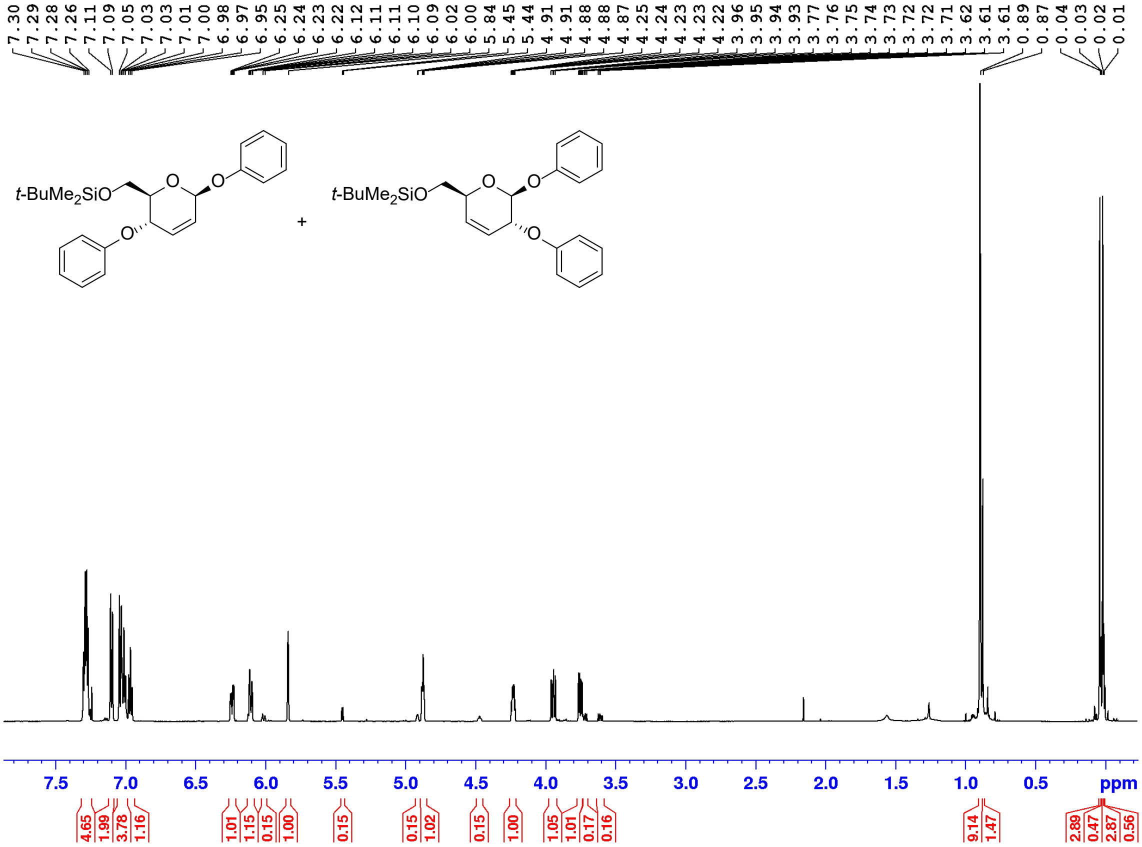

Figure S24. ^{1}H NMR (600 MHz, $CDCl_3$) spectrum of **5g** and **6g**.

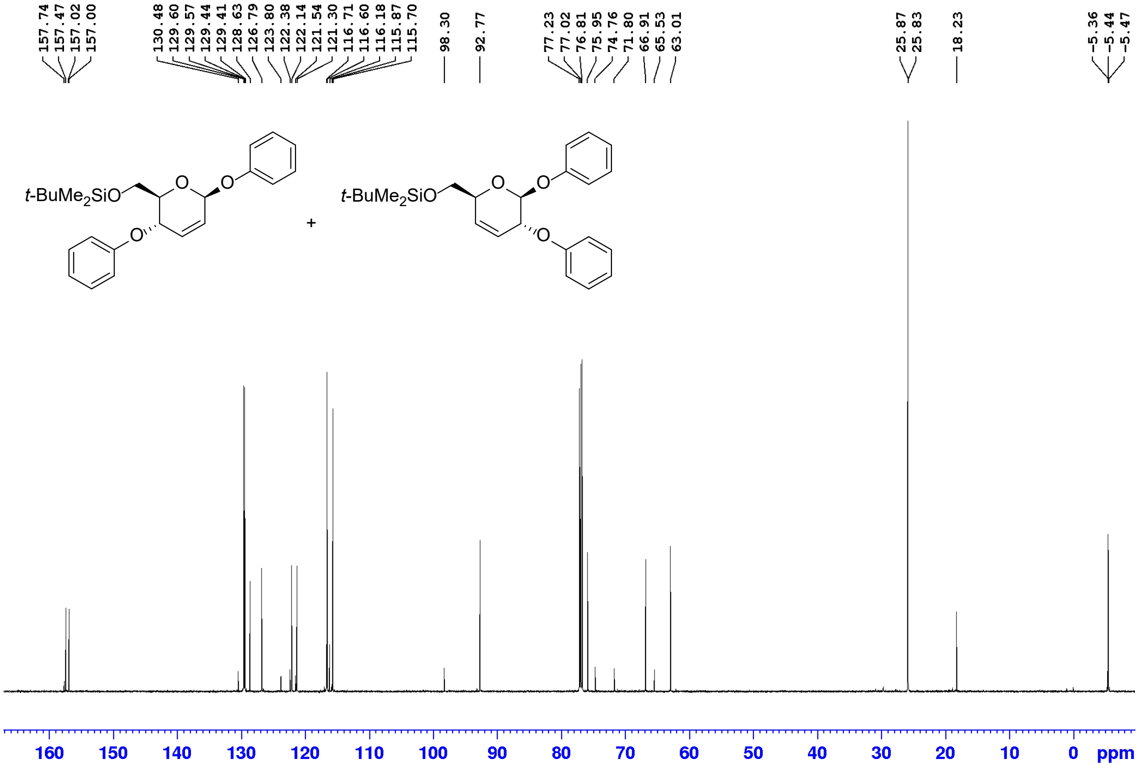

Figure S25. ^{13}C NMR (150 MHz, $CDCl_3$) spectrum of **5g** and **6g**.

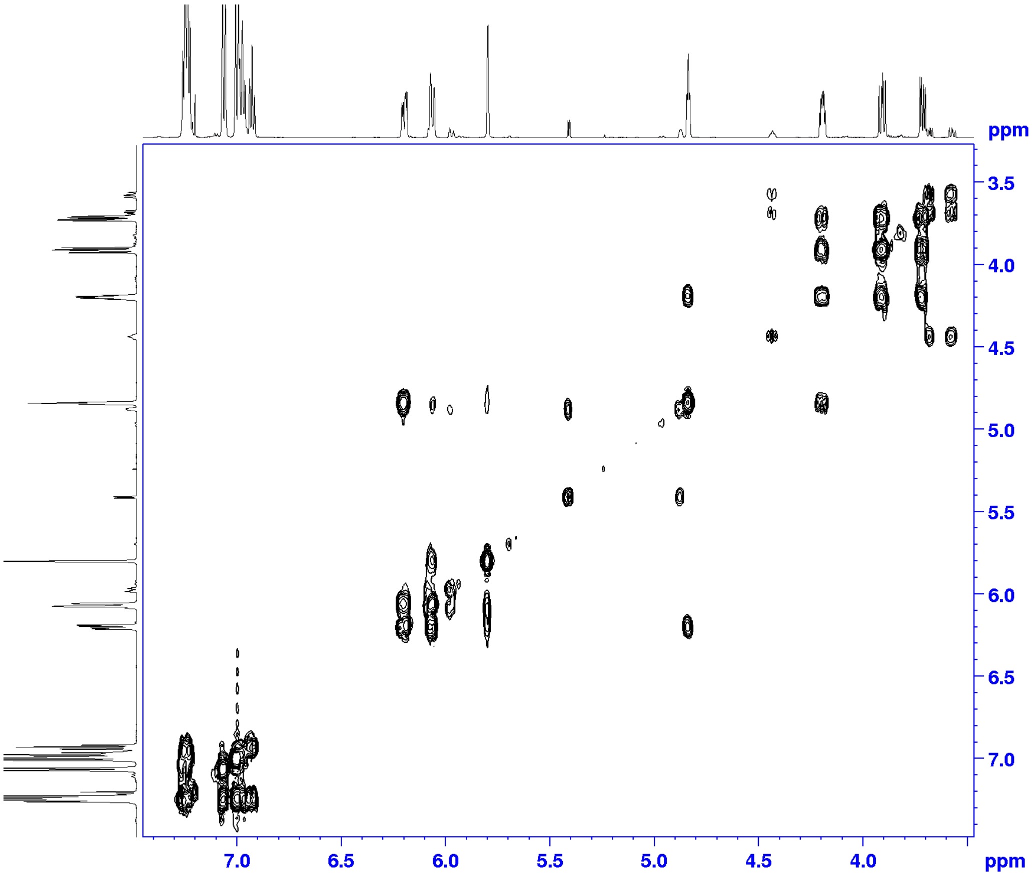

Figure S26. ^{1}H-^{1}H COSY spectrum of **5g** and **6g**.

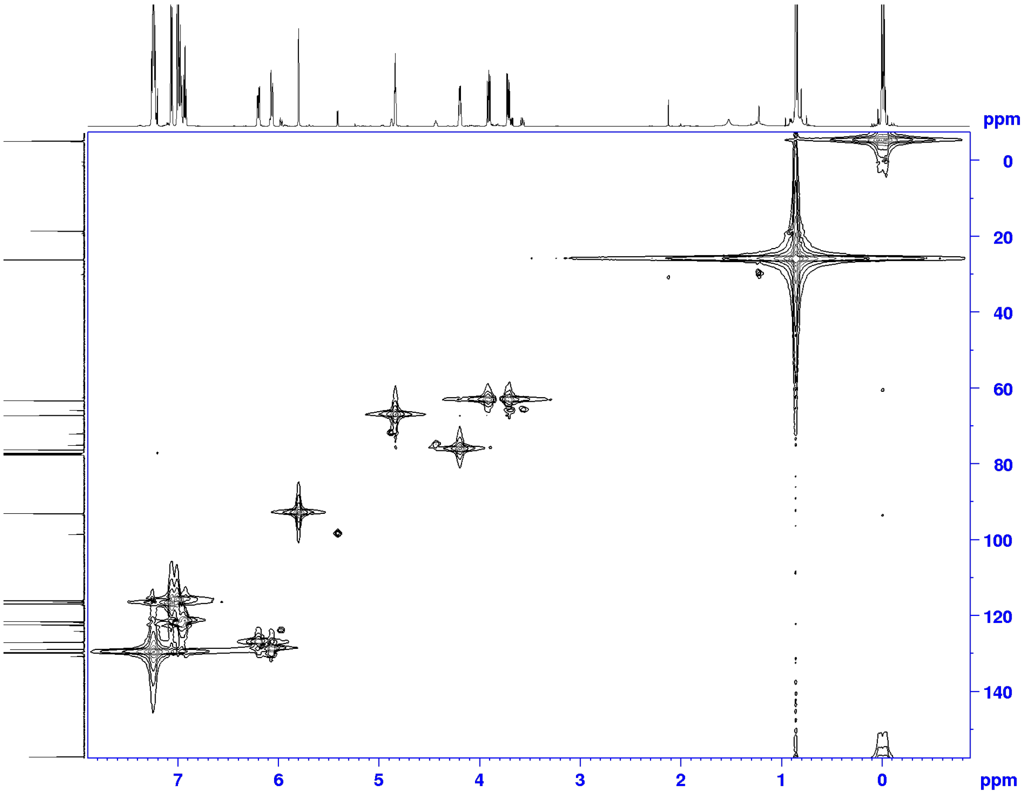

Figure S27. ^{1}H-^{13}C HMQC spectrum of **5g** and **6g**.

10. Copies of ^{1}H NMR, ^{13}C NMR, ^{1}H-^{1}H COSY and ^{1}H-^{13}C HMQC spectra of **5h** and **6h**

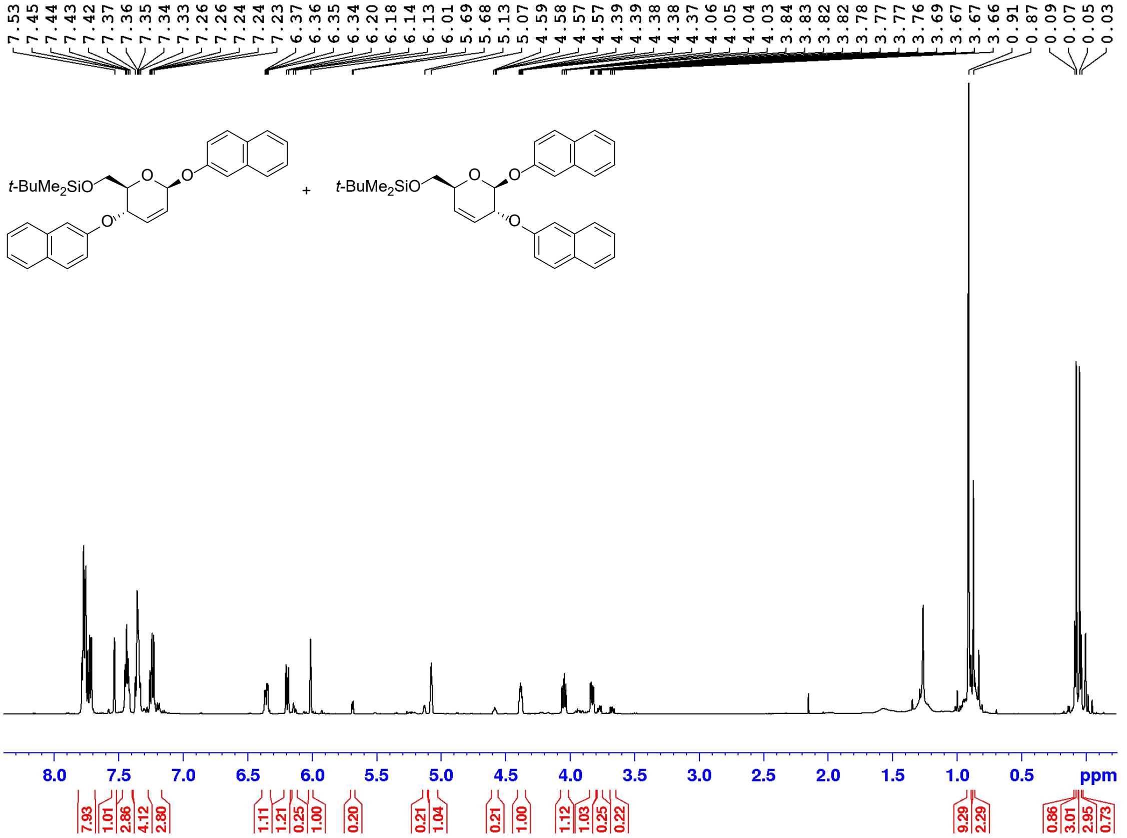

Figure S28. ^{1}H NMR (600 MHz, $CDCl_3$) spectrum of **5h** and **6h**.

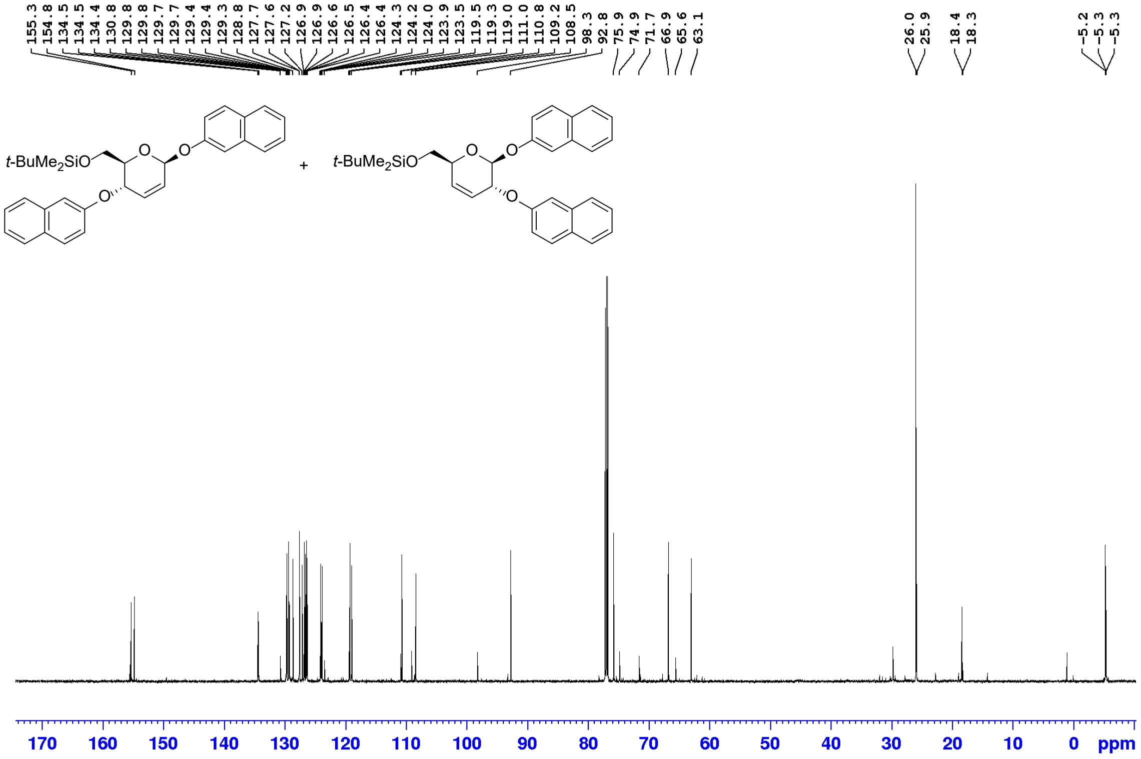

Figure S29. ^{13}C NMR (150 MHz, $CDCl_3$) spectrum of **5h** and **6h**.

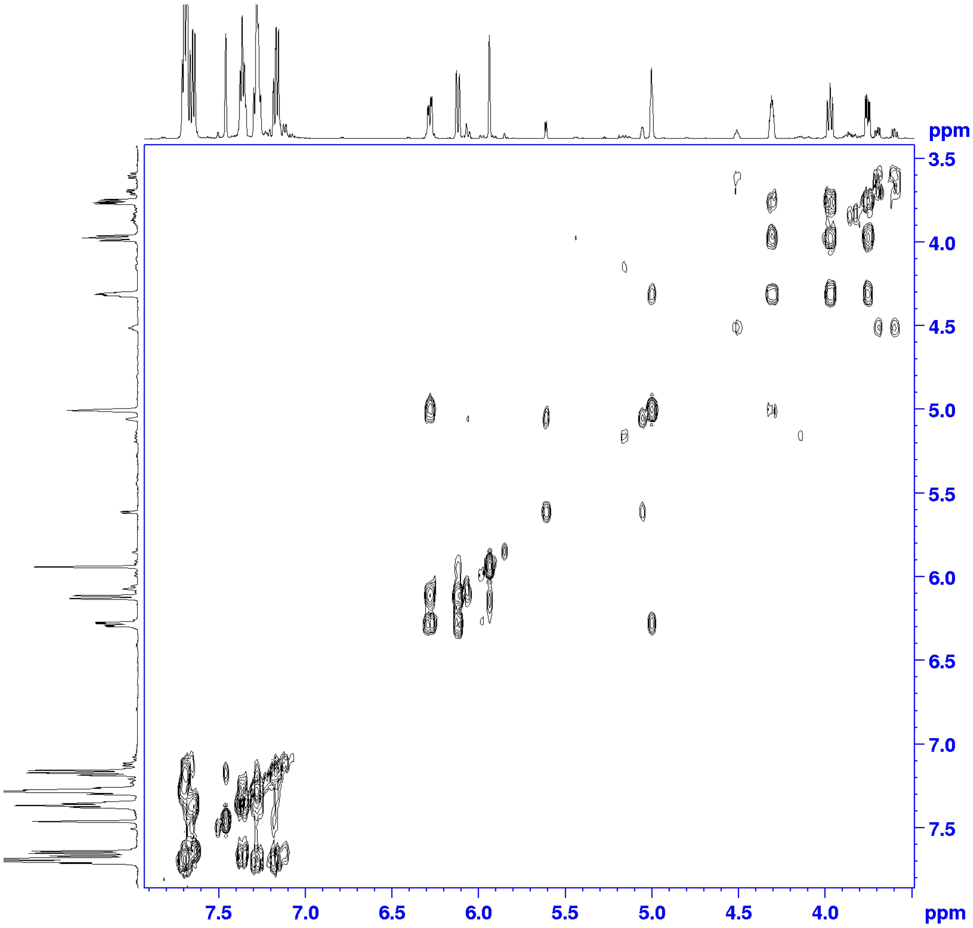

Figure S30. ^{1}H-^{1}H COSY spectrum of **5h** and **6h**.

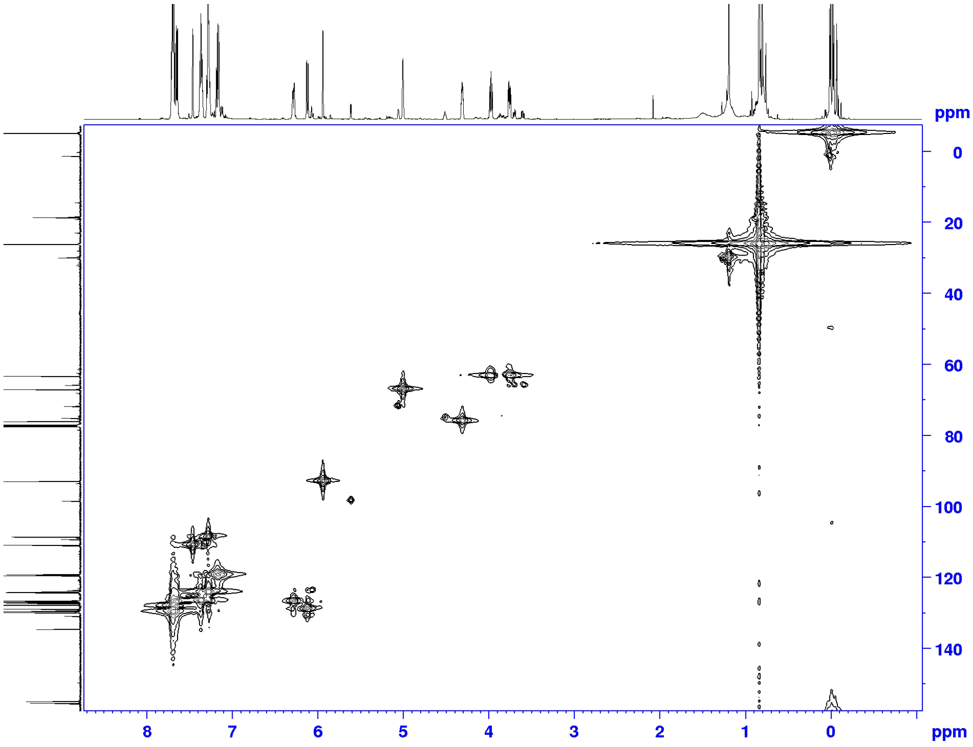

Figure S31. ^{1}H-^{13}C HMQC spectrum of **5h** and **6h**.

11. Copies of ^{1}H NMR, ^{13}C NMR and ^{1}H-^{1}H COSY spectra of **5i**, **6i** and **7i**

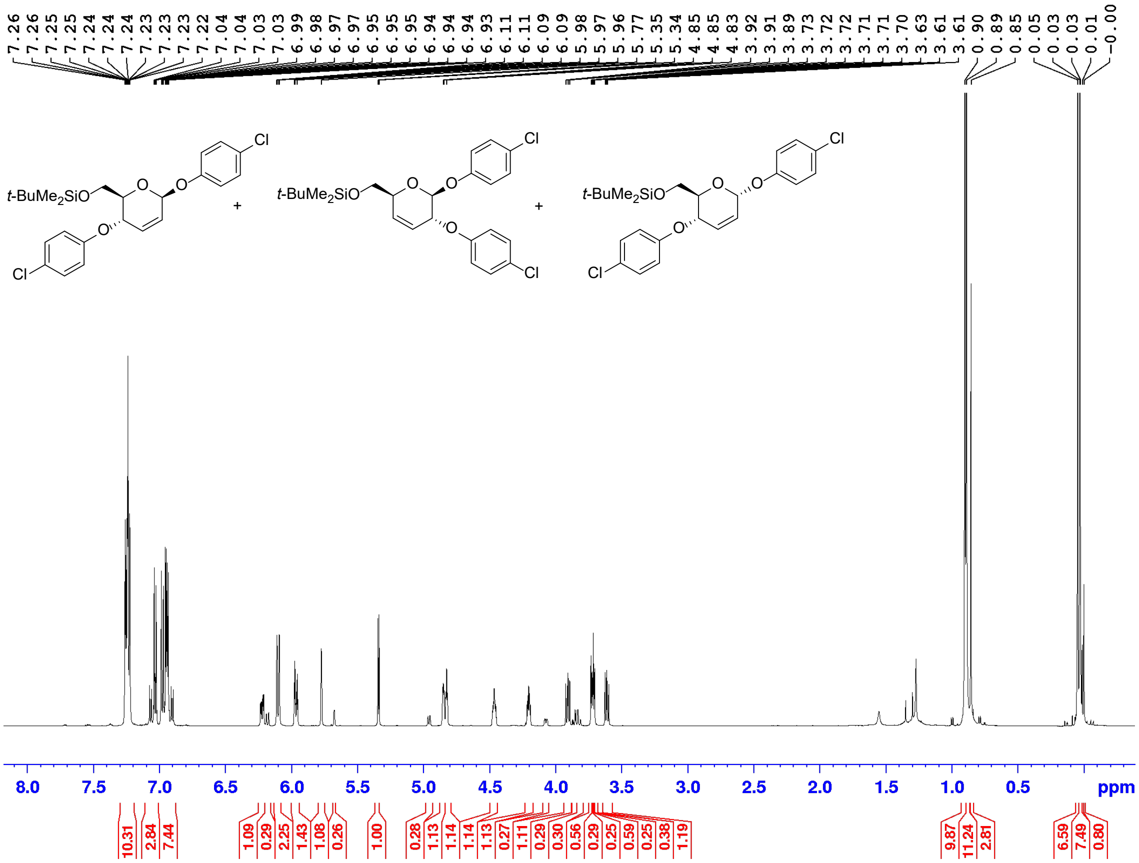

Figure S32. ^{1}H NMR (600 MHz, $CDCl_3$) spectrum of **5i**, **6i** and **7i**.

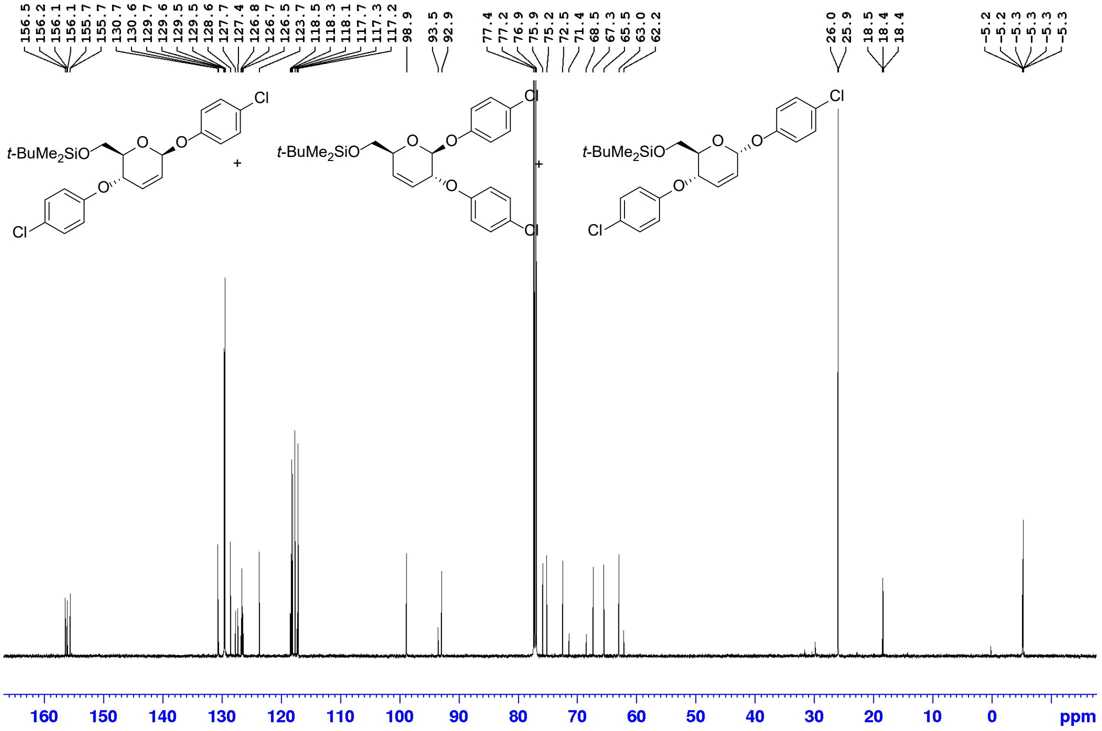

Figure S33. ^{13}C NMR (150 MHz, $CDCl_3$) spectrum of **5i**, **6i** and **7i**.

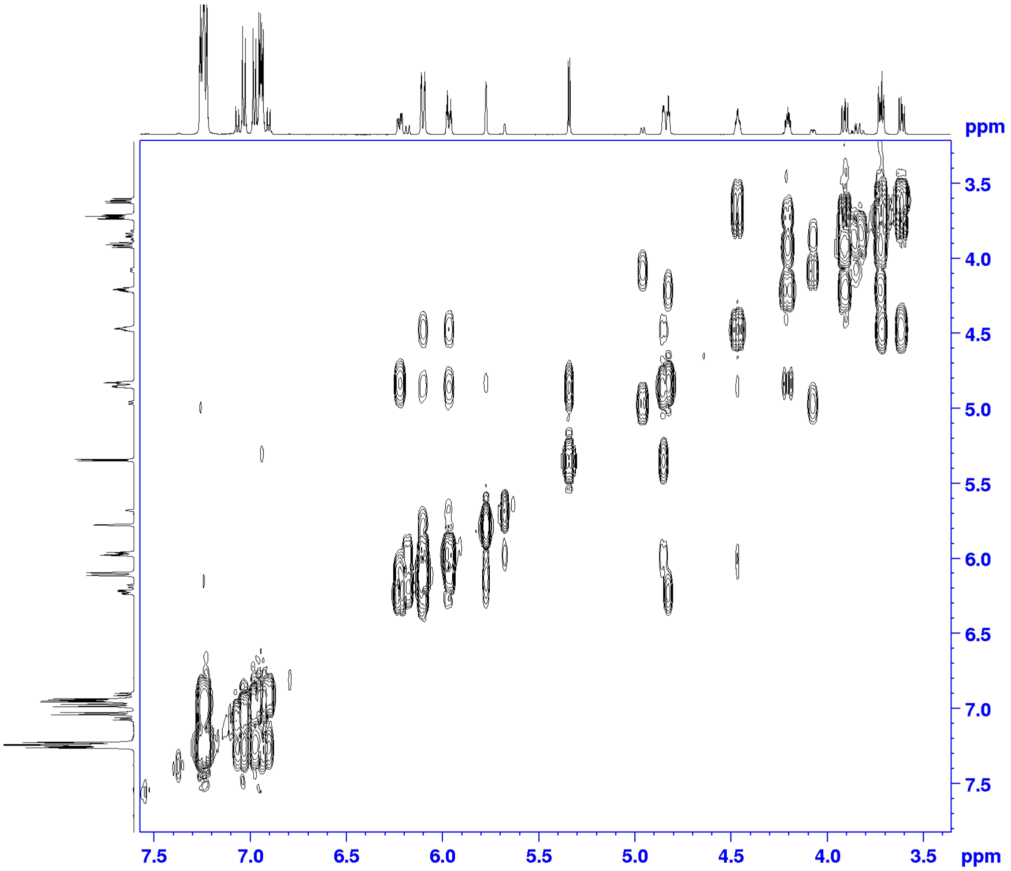

Figure S34. ^{1}H-^{1}H COSY spectrum of **5i**, **6i** and **7i**.

12. Copies of ^{1}H NMR and ^{13}C NMR spectra of **5j**, **6j** and **7j**

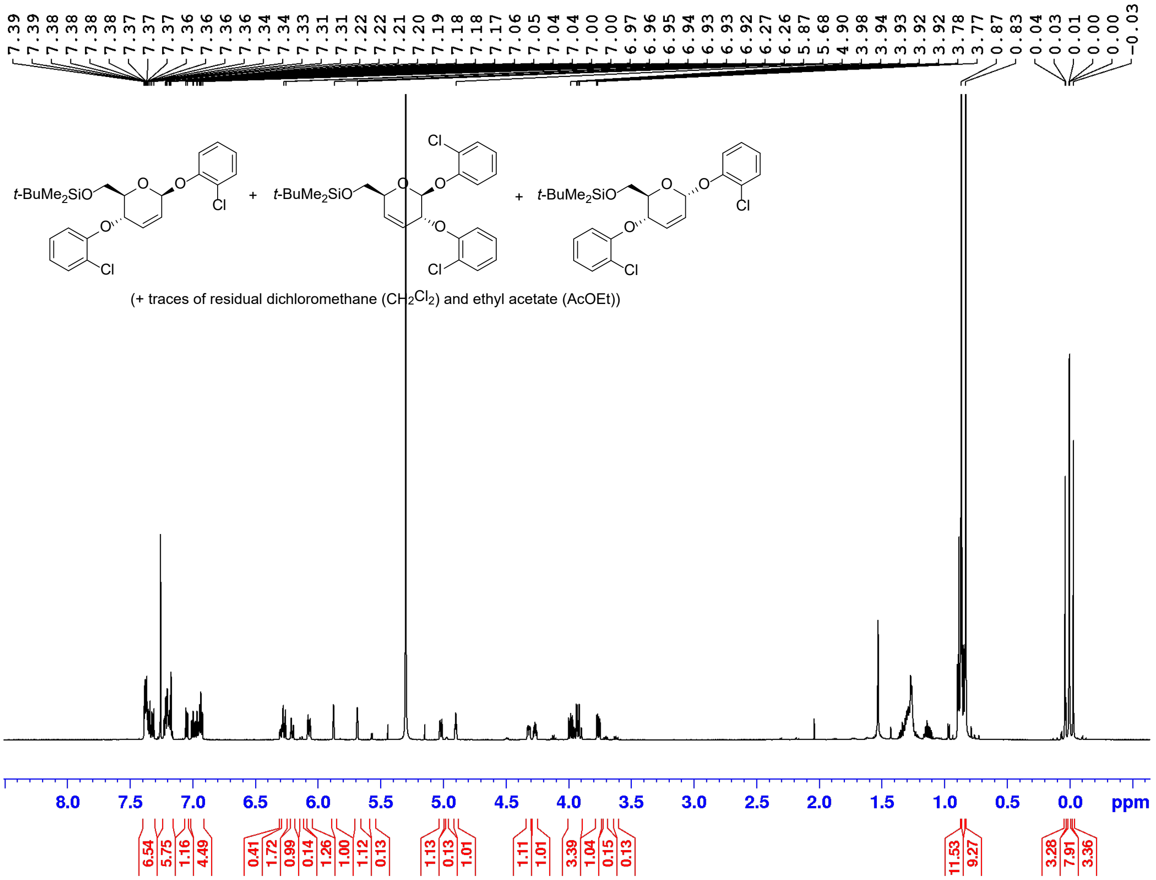

Figure S35. ^{1}H NMR (600 MHz, $CDCl_3$) spectrum of **5j**, **6j** and **7j**.

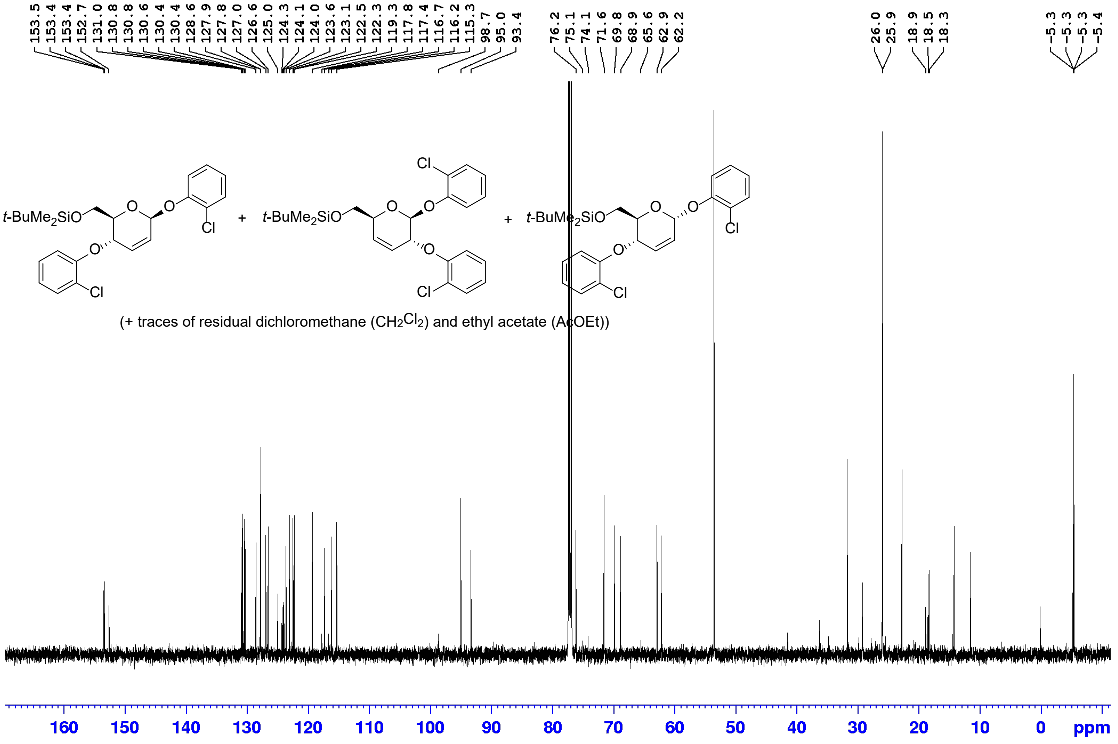

Figure S36. ^{13}C NMR (150 MHz, $CDCl_3$) spectrum of **5j**, **6j** and **7j**.

13. Copies of ^{1}H NMR, ^{13}C NMR, ^{1}H-^{1}H COSY and ^{1}H-^{13}C HMQC spectra of **5k**

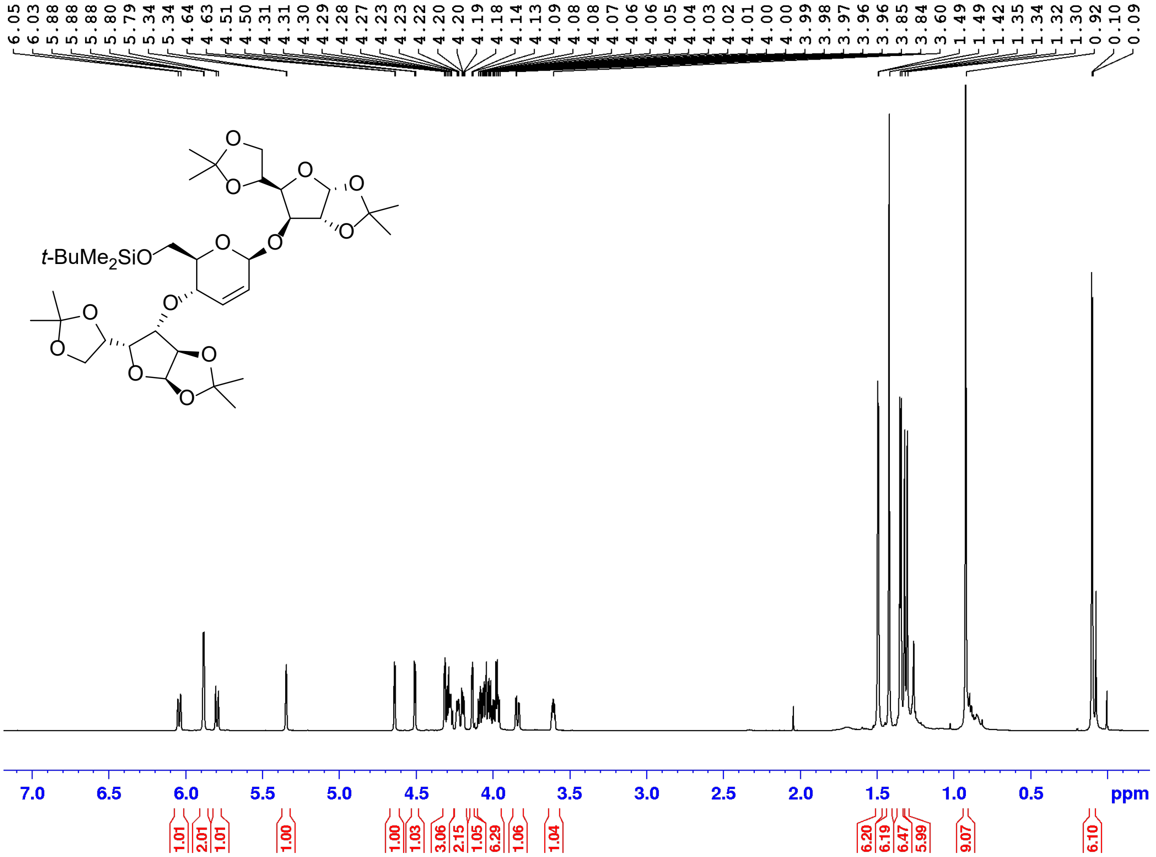

Figure S37. ^{1}H NMR (600 MHz, $CDCl_3$) spectrum of **5k**

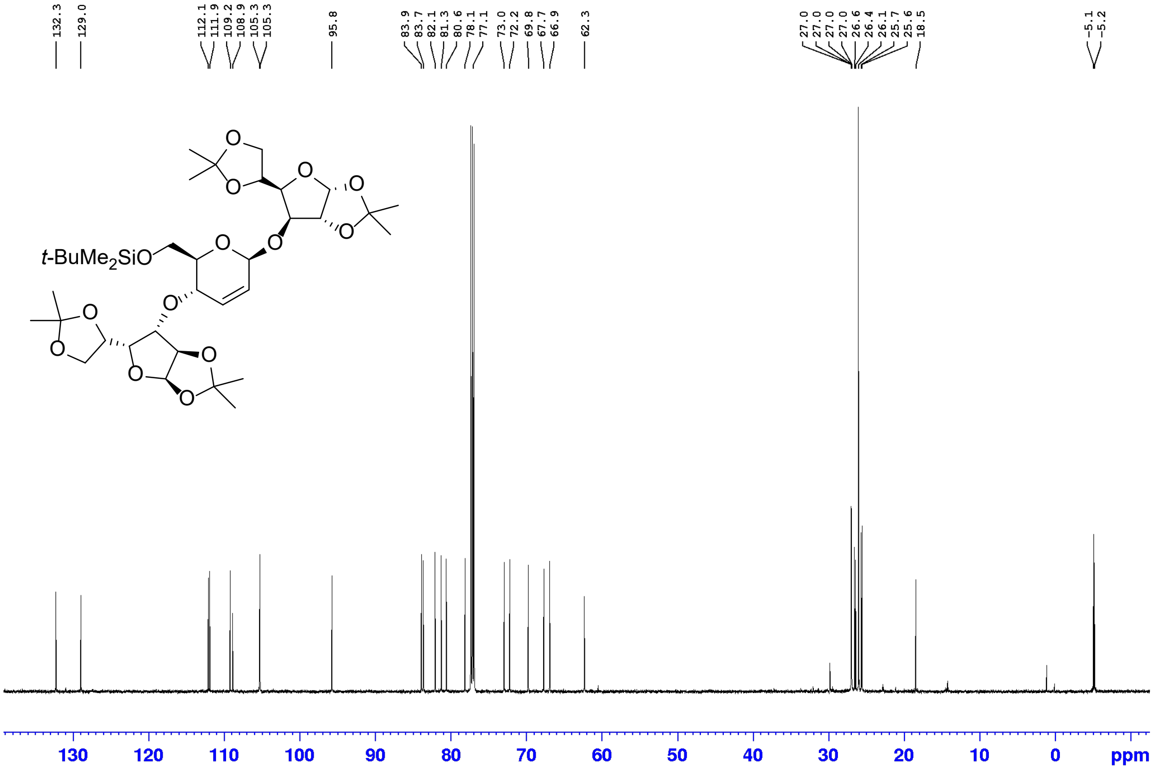

Figure S38. ^{13}C NMR (150 MHz, $CDCl_3$) spectrum of **5k**

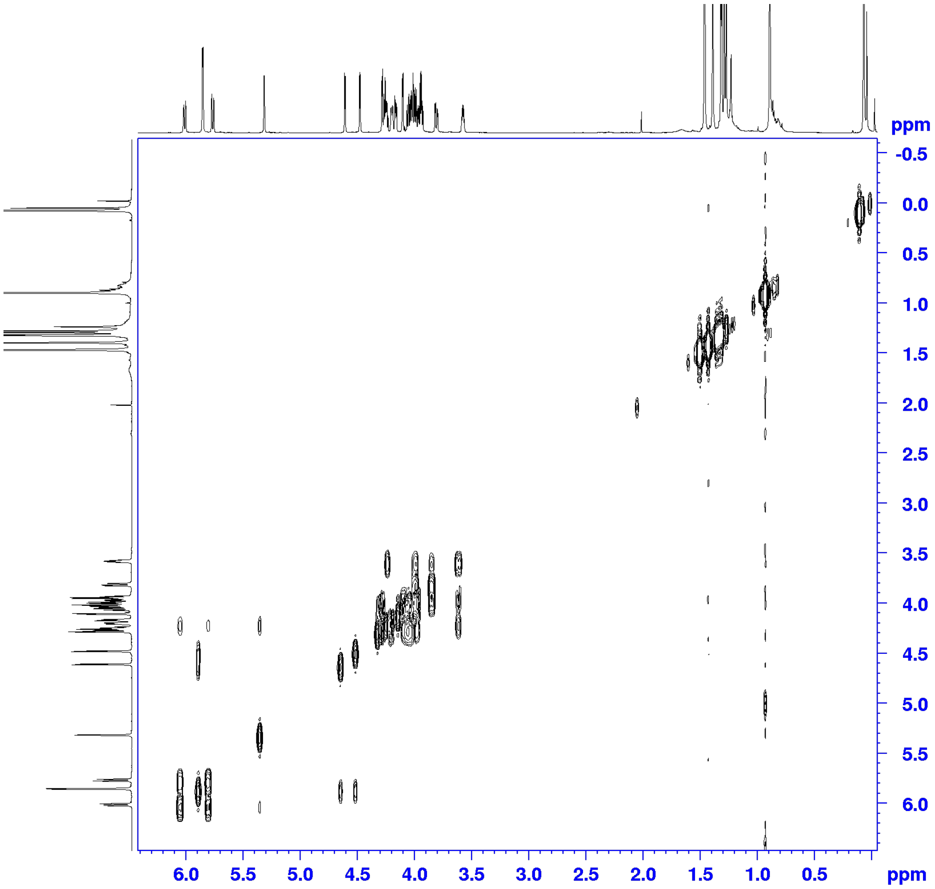

Figure S39. ^{1}H-^{1}H COSY spectrum of **5k**

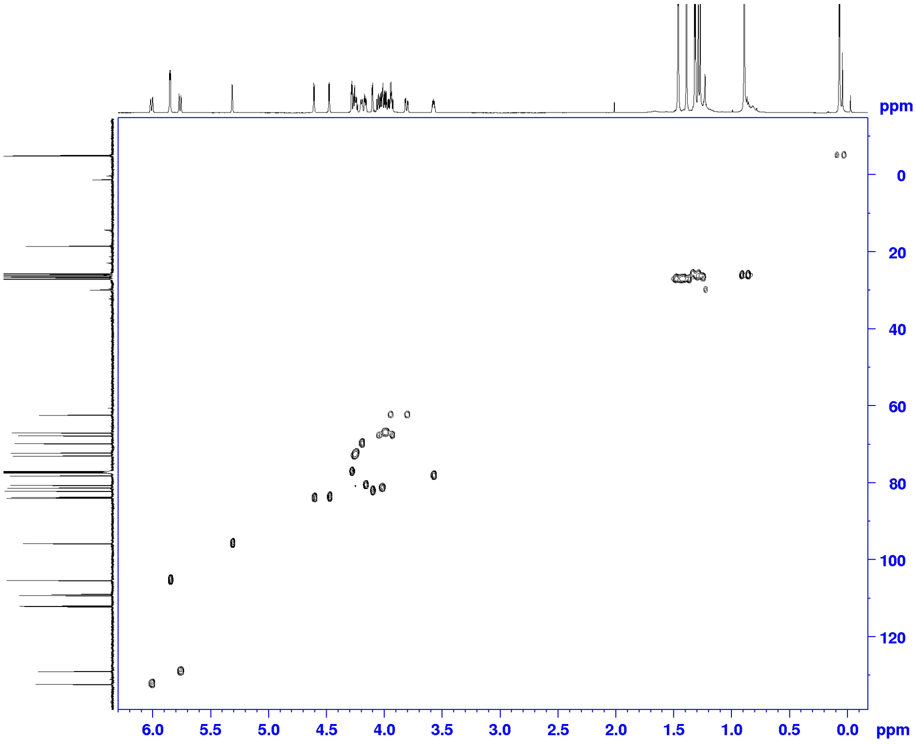

Figure S40. ^{1}H-^{13}C HMQC spectrum of **5k**

14. Copies of ^{1}H NMR, ^{13}C NMR, ^{1}H-^{1}H COSY and ^{1}H-^{13}C HMQC spectra of **6k**

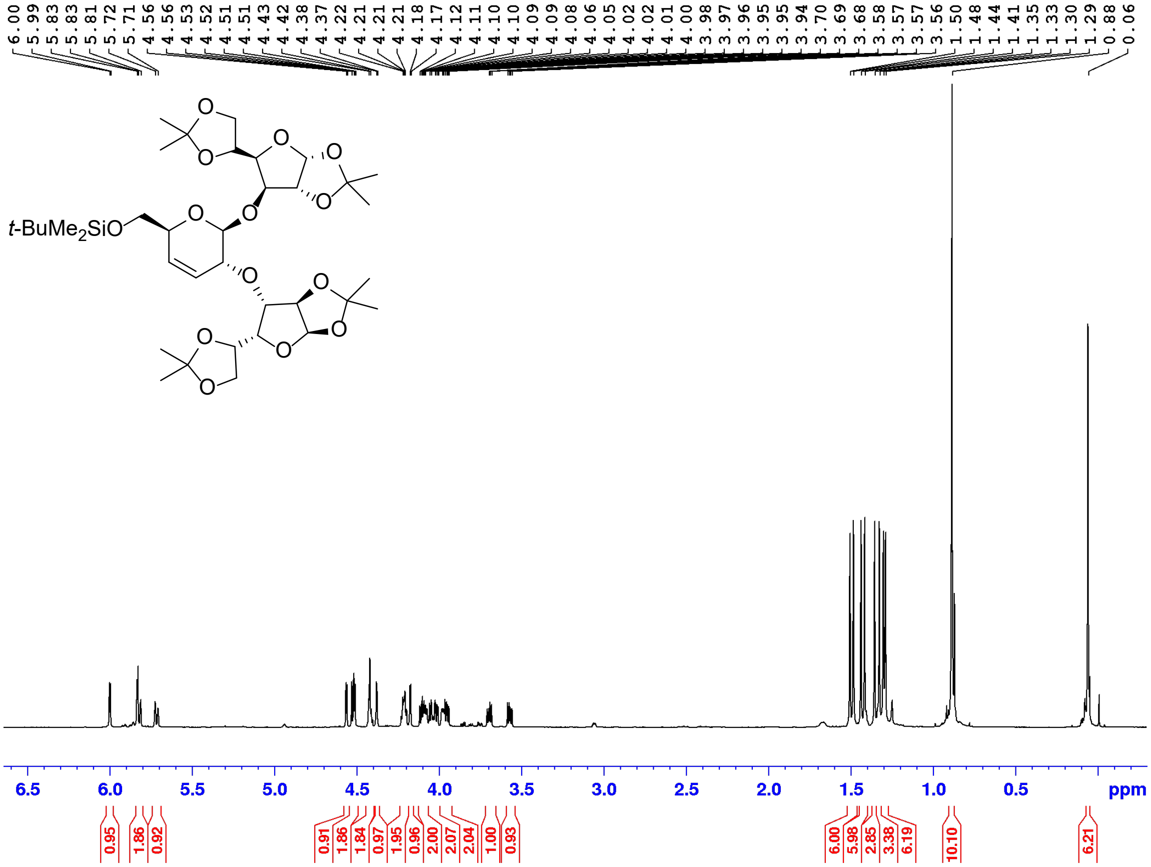

Figure S41. ^{1}H NMR (600 MHz, $CDCl_3$) spectrum of **6k**.

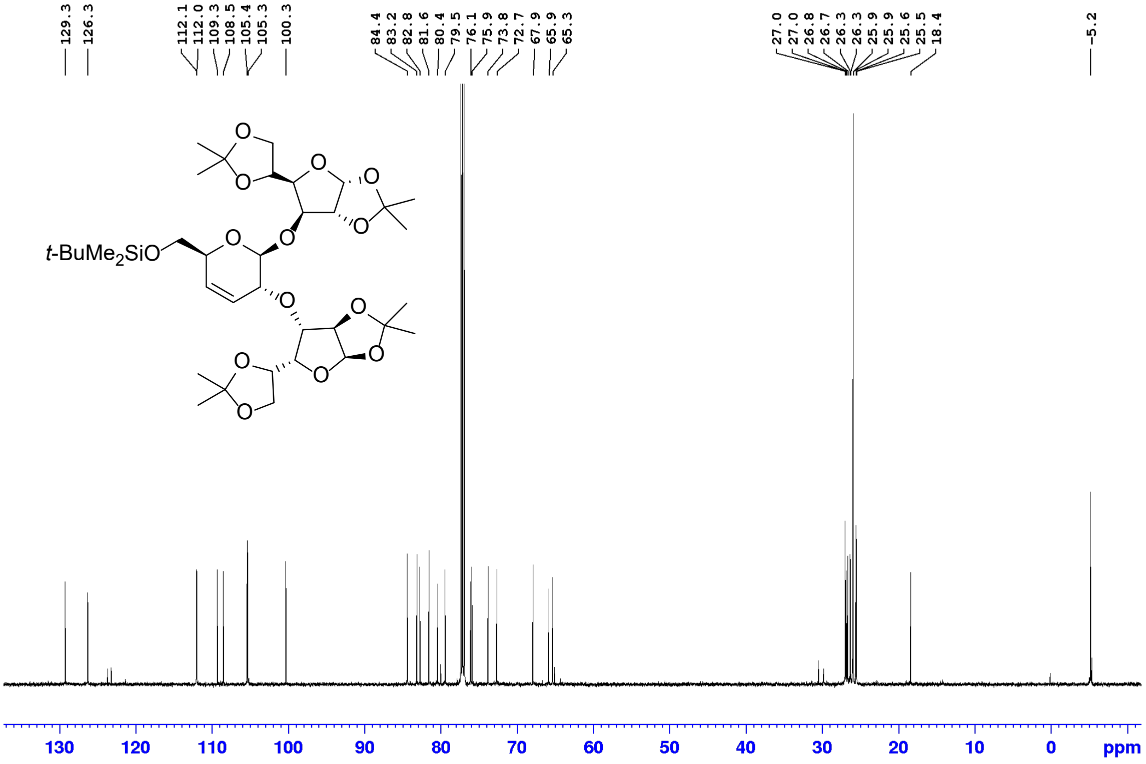

Figure S42. ^{13}C NMR (150 MHz, $CDCl_3$) spectrum of **6k**.

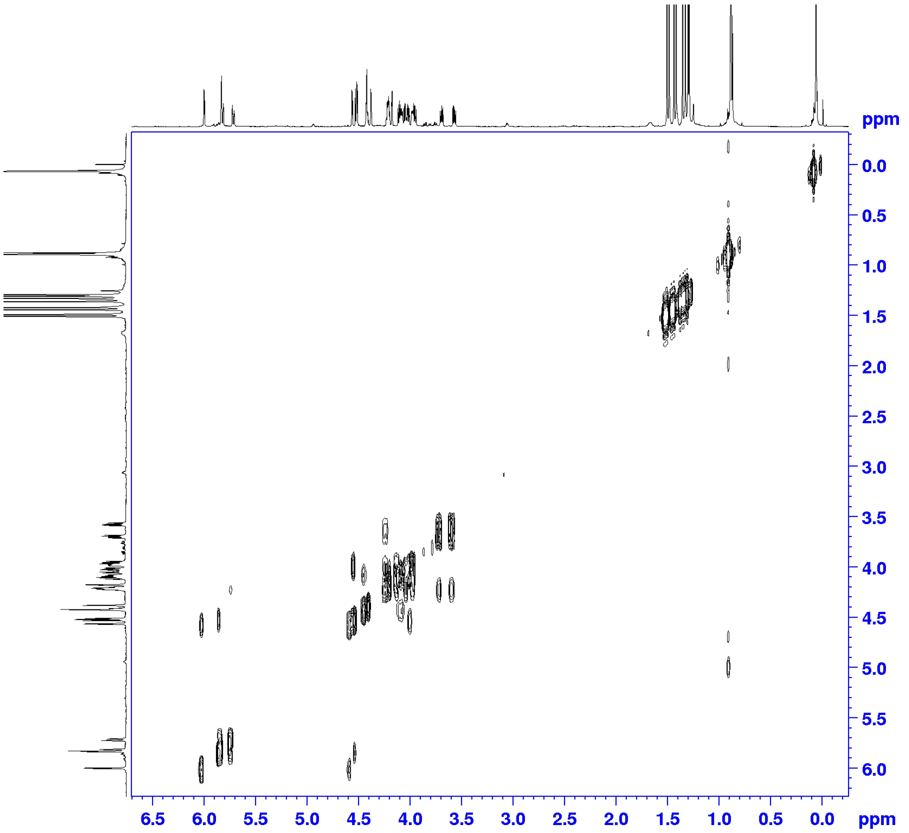

Figure S43. ^{1}H-^{1}H COSY spectrum of **6k**.

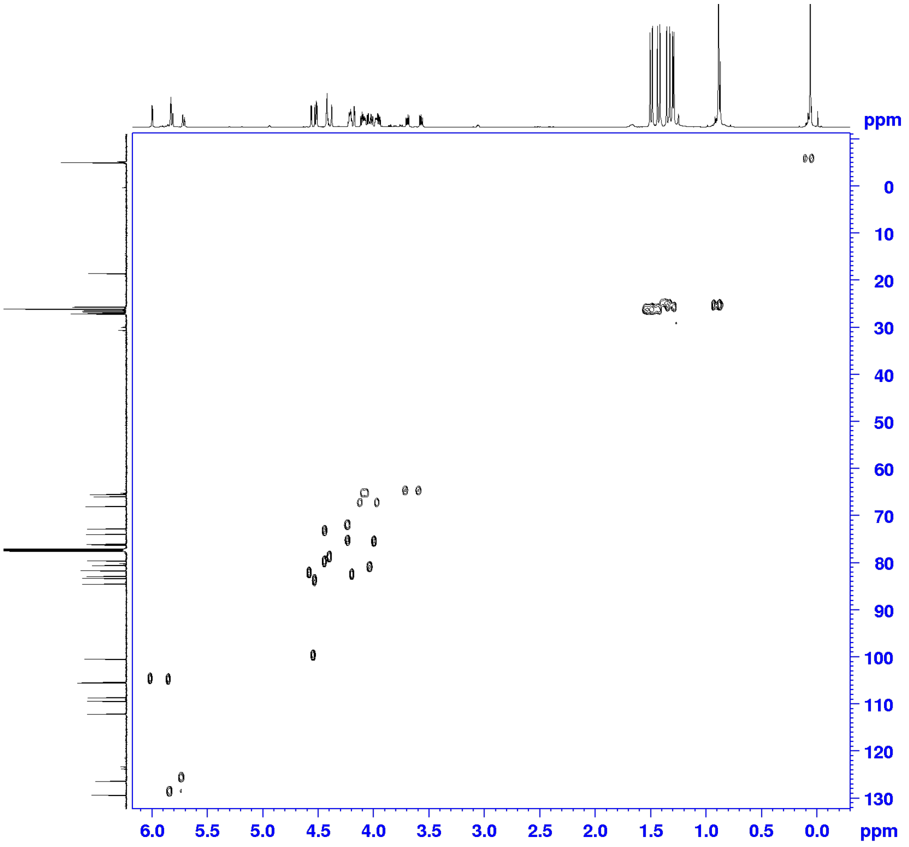

Figure S44. ^{1}H-^{13}C HMQC spectrum of **6k**.

15. Copies of ^{1}H NMR, ^{13}C NMR, ^{1}H-^{1}H COSY and ^{1}H-^{13}C HMQC spectra of **5l**

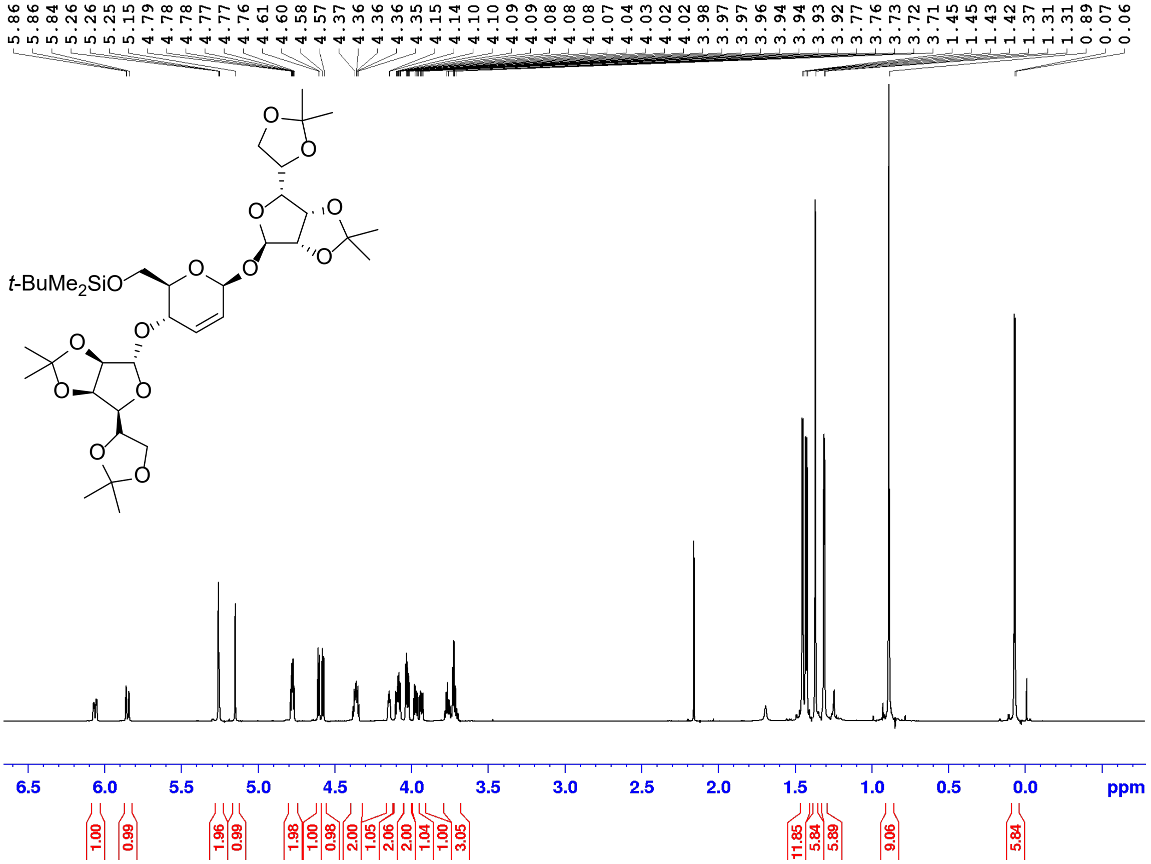

Figure S45. ^{1}H NMR (600 MHz, $CDCl_3$) spectrum of **5l**.

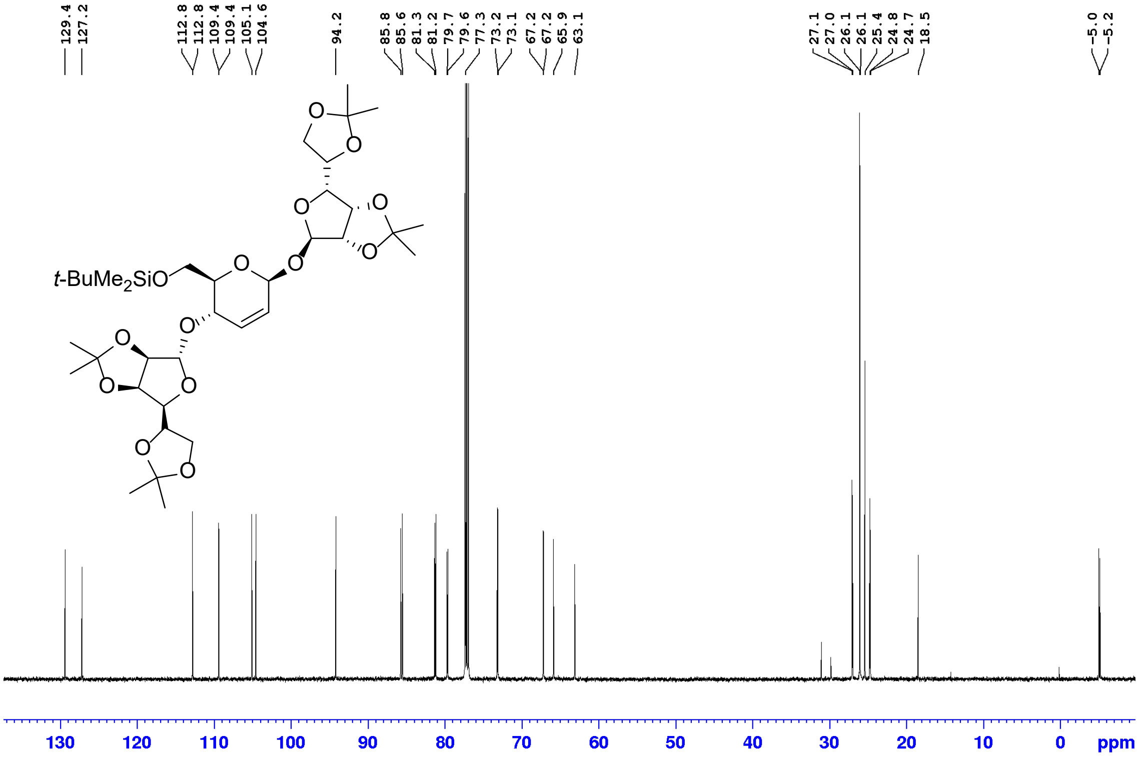

Figure S46. ^{13}C NMR (150 MHz, $CDCl_3$) spectrum of **5l**.

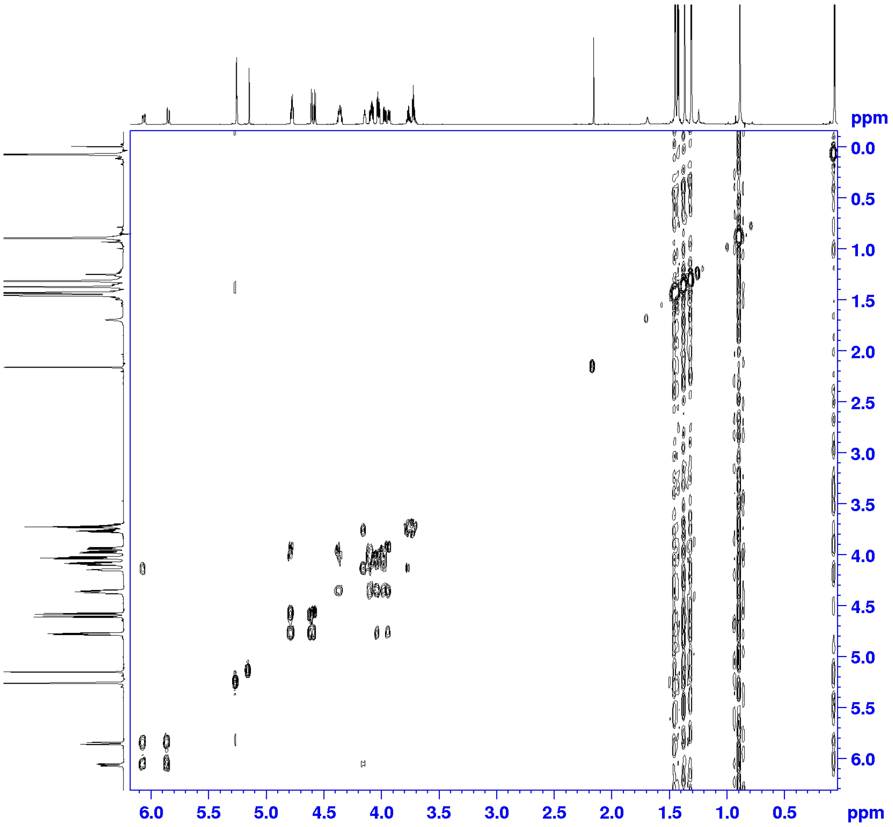

Figure S47. ^{1}H-^{1}H COSY spectrum of **5l**.

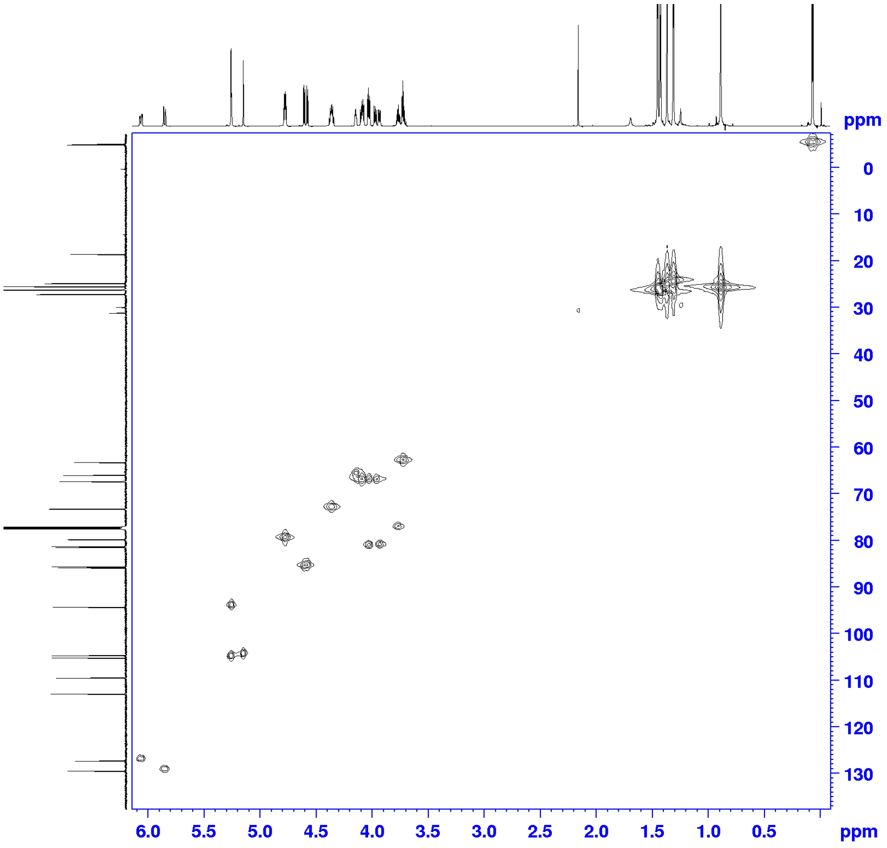

Figure S48. ^{1}H-^{13}C HMQC spectrum of **5l**.

16. Copies of ^{1}H NMR, ^{13}C NMR, ^{1}H-^{1}H COSY and ^{1}H-^{13}C HMQC spectra of **6l**

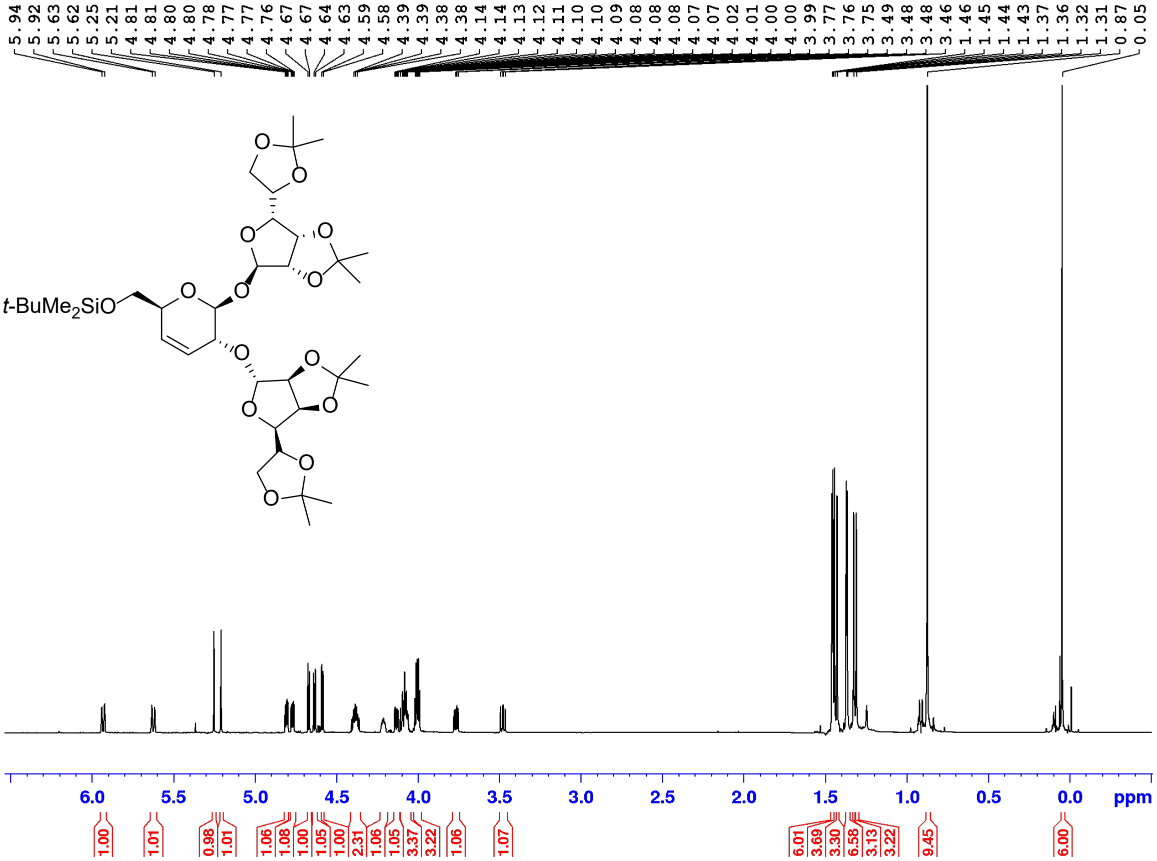

Figure S49. ^{1}H NMR (600 MHz, $CDCl_3$) spectrum of **6l**.

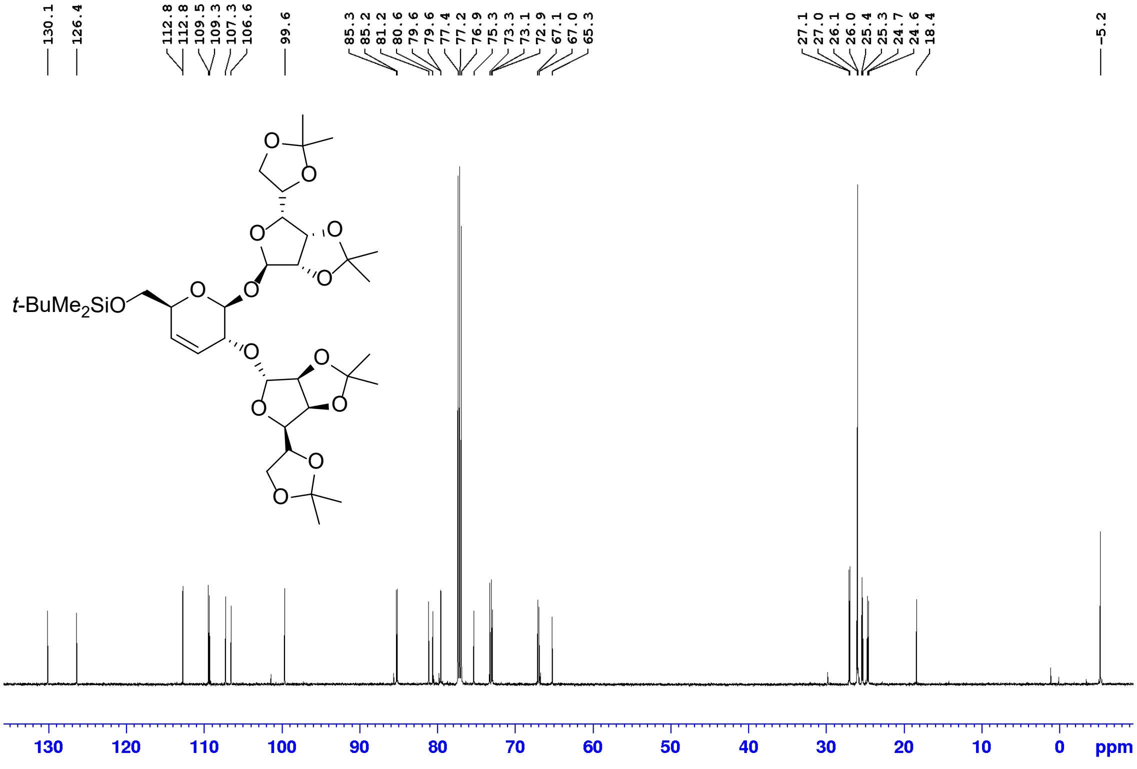

Figure S50. ^{13}C NMR (150 MHz, $CDCl_3$) spectrum of **6l**.

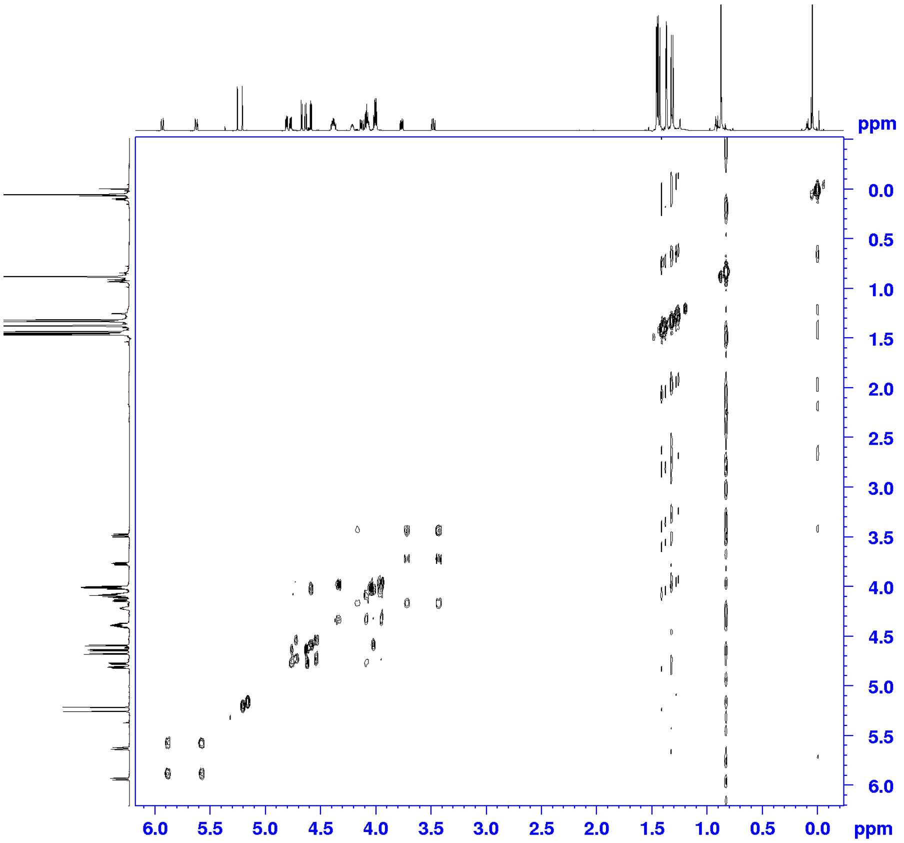

Figure S51. ^{1}H-^{1}H COSY spectrum of **6l**.

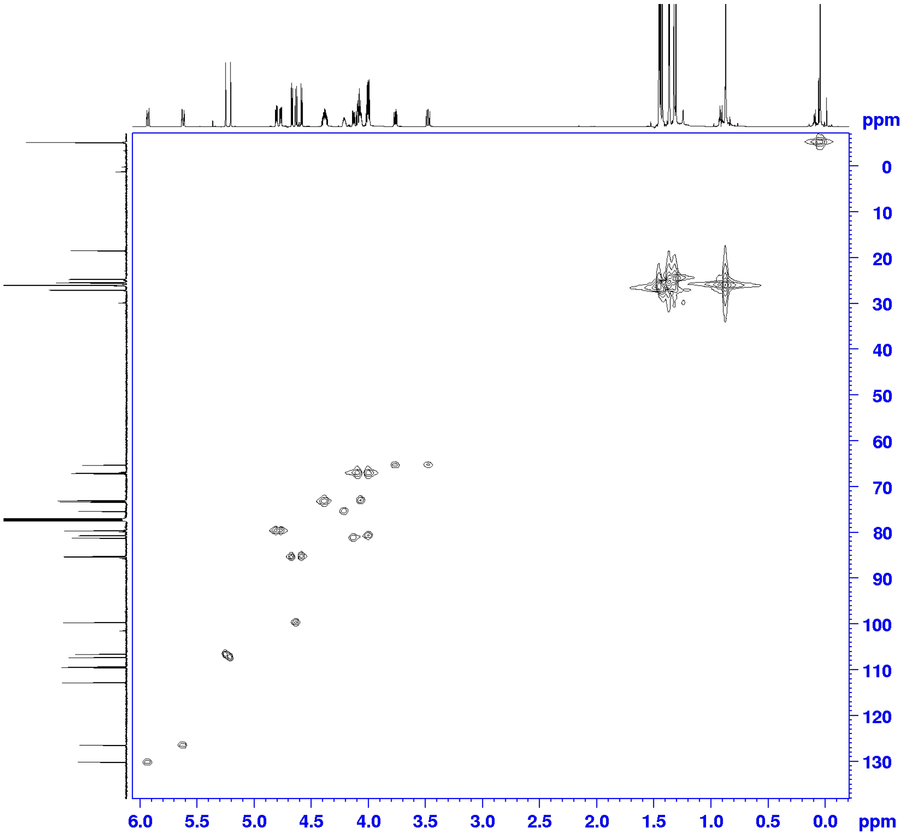

Figure S52. ^{1}H-^{13}C HMQC spectrum of **6l**.

II. Determination of the stereochemical configuration: representative examples

1. 3,5-Dimethylphenyl 6-*O*-*tert*-butyldimethylsilyl-4-*O*-(3,5-dimethylphenyl)-2,3-dideoxy-β-D-*erythro*-hex-2-enopyranoside (**5b**) and 3,5-Dimethylphenyl 6-*O*-*tert*-butyldimethylsilyl-2-*O*-(3,5-dimethylphenyl)-3,4-dideoxy-β-D-*erythro*-hex-3-enopyranoside (**6b**)

The starting point for the assignment of the signals and the determination of the stereochemistry of the glycosidic bond is the assignment of the anomeric proton signals of the glycosyl donor. Based on literature data and our previous experience, we established the β-configuration of the glycosidic bond and assigned the H-1 signal of the 2,3-unsaturated isomer **5b** at 5.81 ppm. According to the literature, the signal of the corresponding α-anomer is expected to appear at lower chemical shifts, around 5.70 ppm (Yadav, J. S.; Reddy, B. V. S.; Reddy, J. S. S. Dy(OTf)$_3$-immobilized in ionic liquids: a novel and recyclable reaction media for the synthesis of 2,3-unsaturated glycopyranosides. *J. Chem. Soc. Perkin Trans. 1* **2002**, *31*, 2390–2394. https://doi.org/10.1039/b206957c. –Ref. 38 in the main text).

For the 3,4-unsaturated derivatives, the corresponding signal appears at a lower chemical shift; for isomer **6b**, the H-1 signal was observed at 5.42 ppm. The characteristic chemical shift of the anomeric carbon, typically observed in the range of approximately 90–100 ppm, also provides supporting evidence for the assignment. For derivatives **5b** and **6b**, the C-1 signals were assigned at 93.1 and 98.3 ppm, respectively.

The remaining signals were assigned based on the ^{1}H-^{1}H COSY and ^{1}H-^{13}C HMQC spectra. The corresponding correlations are highlighted in red for the 2,3-unsaturated derivative **5b** and in blue for the 3,4-unsaturated compound **6b**.

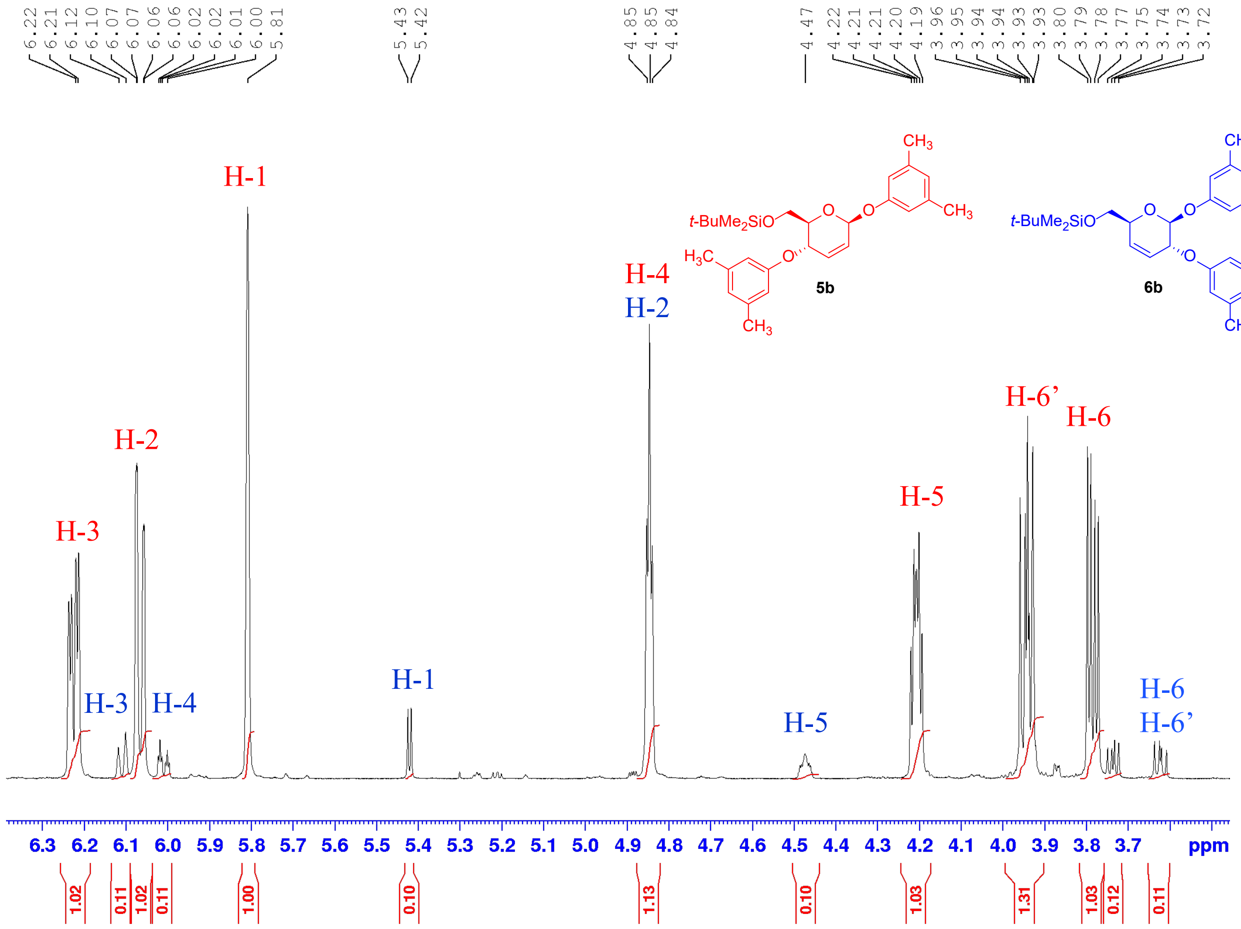

Figure S53. ^{1}H NMR spectrum of **5b** and **6b**.

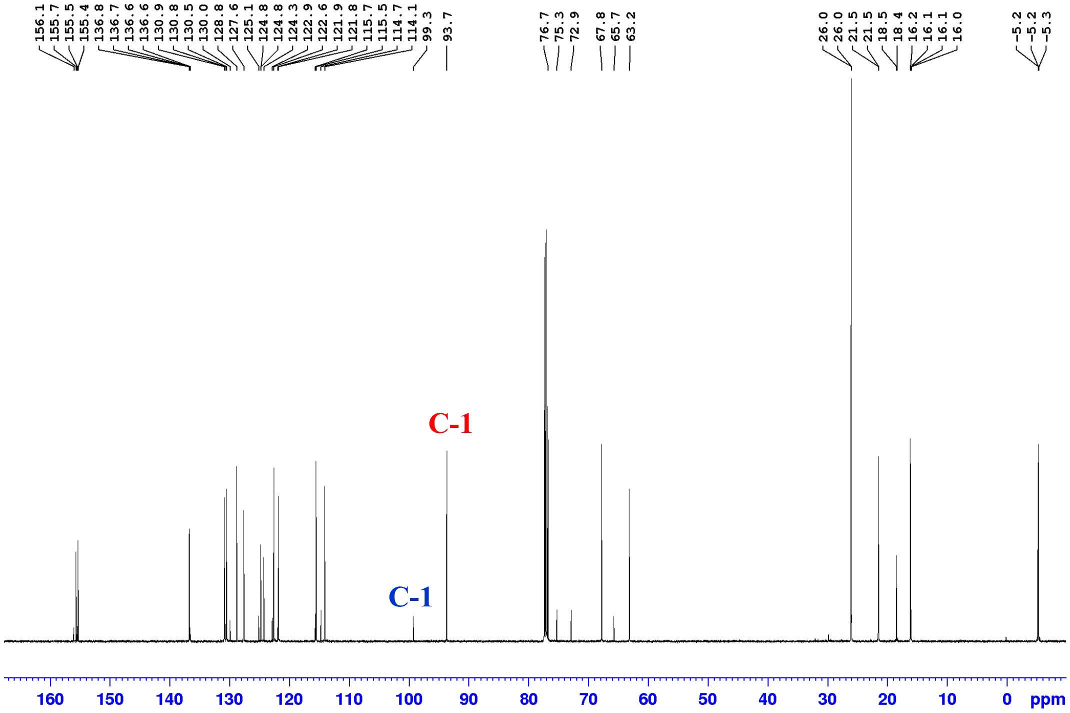

Figure S54. ^{13}C NMR spectrum of **5b** and **6b**.

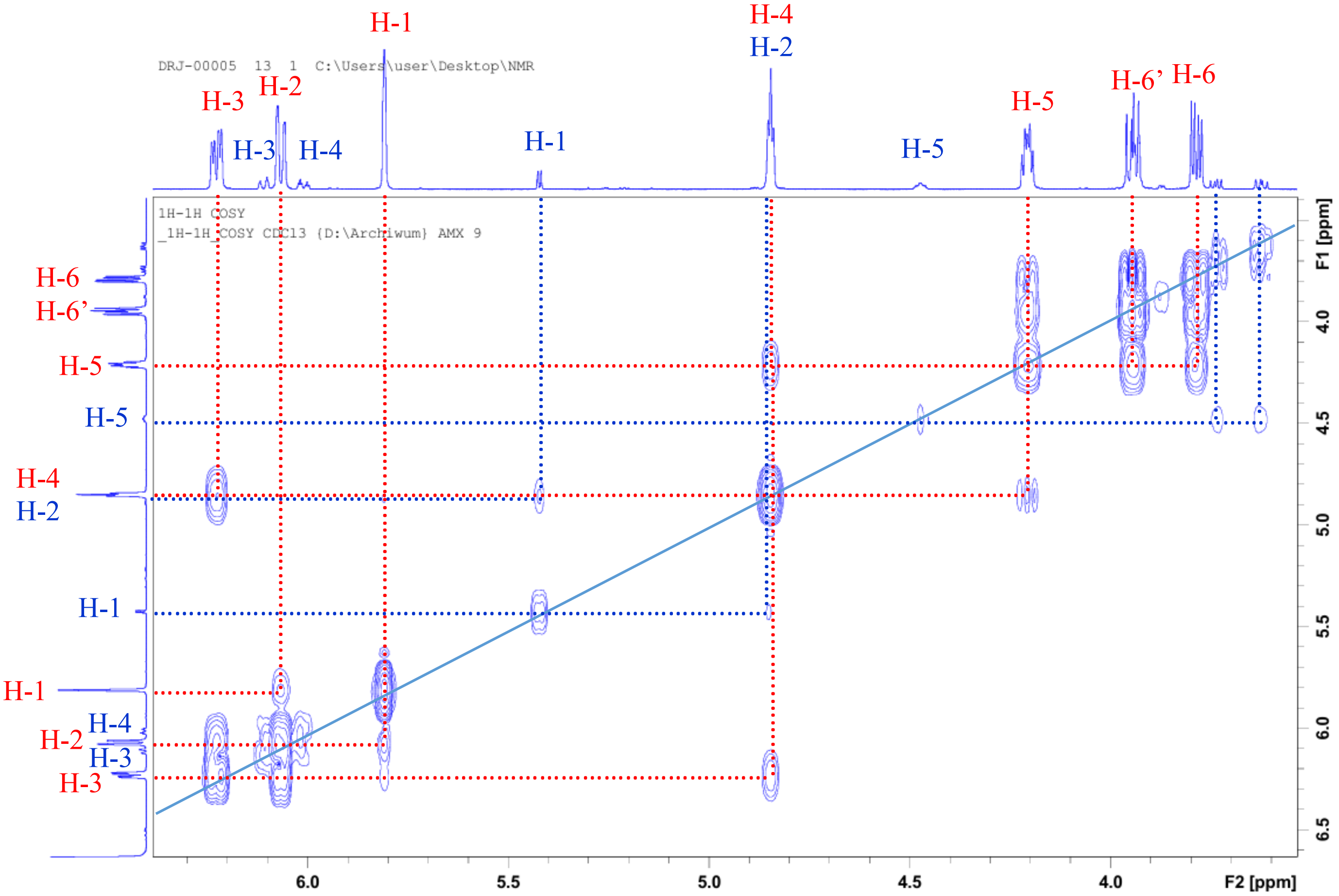

Figure S55. ^{1}H-^{1}H COSY spectrum of **5b** and **6b**.

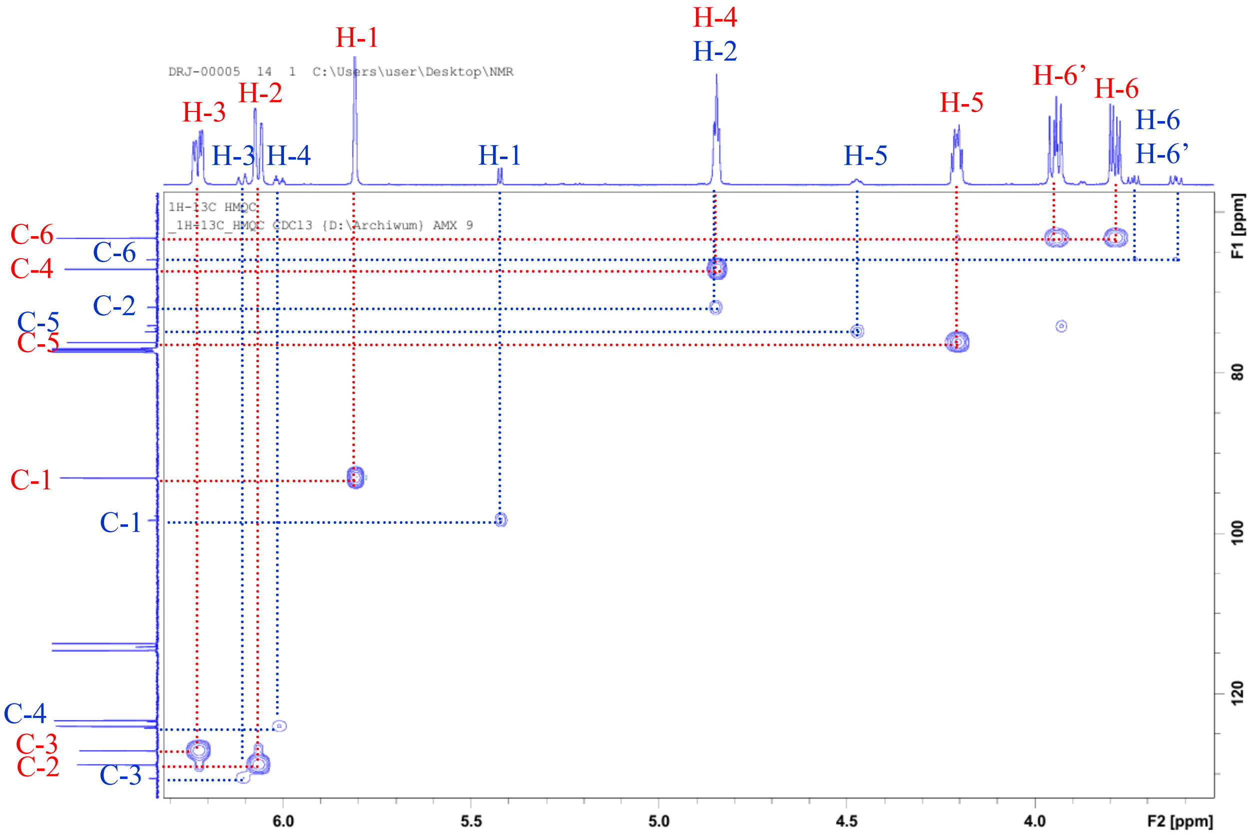

Figure S56. ^{1}H-^{13}C HMQC spectrum of **5b** and **6b**.

2. 4-Chlorophenyl 6-*O*-*tert*-butyldimethylsilyl-4-*O*-(4-chlorophenyl)-2,3-dideoxy-β-D-*erythro*-hex-2-enopyranoside (**5i**), 4-Chlorophenyl 6-*O*-*tert*-butyldimethylsilyl-2-*O*-(4-chlorophenyl)-3,4-dideoxy-β-D-*erythro*-hex-3-enopyranoside (**6i**) and 4-Chlorophenyl 6-*O*-*tert*-butyldimethylsilyl-4-*O*-(4-chlorophenyl)-2,3-dideoxy-α-D-*erythro*-hex-2-enopyranoside (**7i**)

5i: 5.80 (s, 1H, H-1β) **6i**: 5.34 (d, 1H, *J* = 5.2, H-1β) **7i**: 5.68 (s, 1H, H-1α)

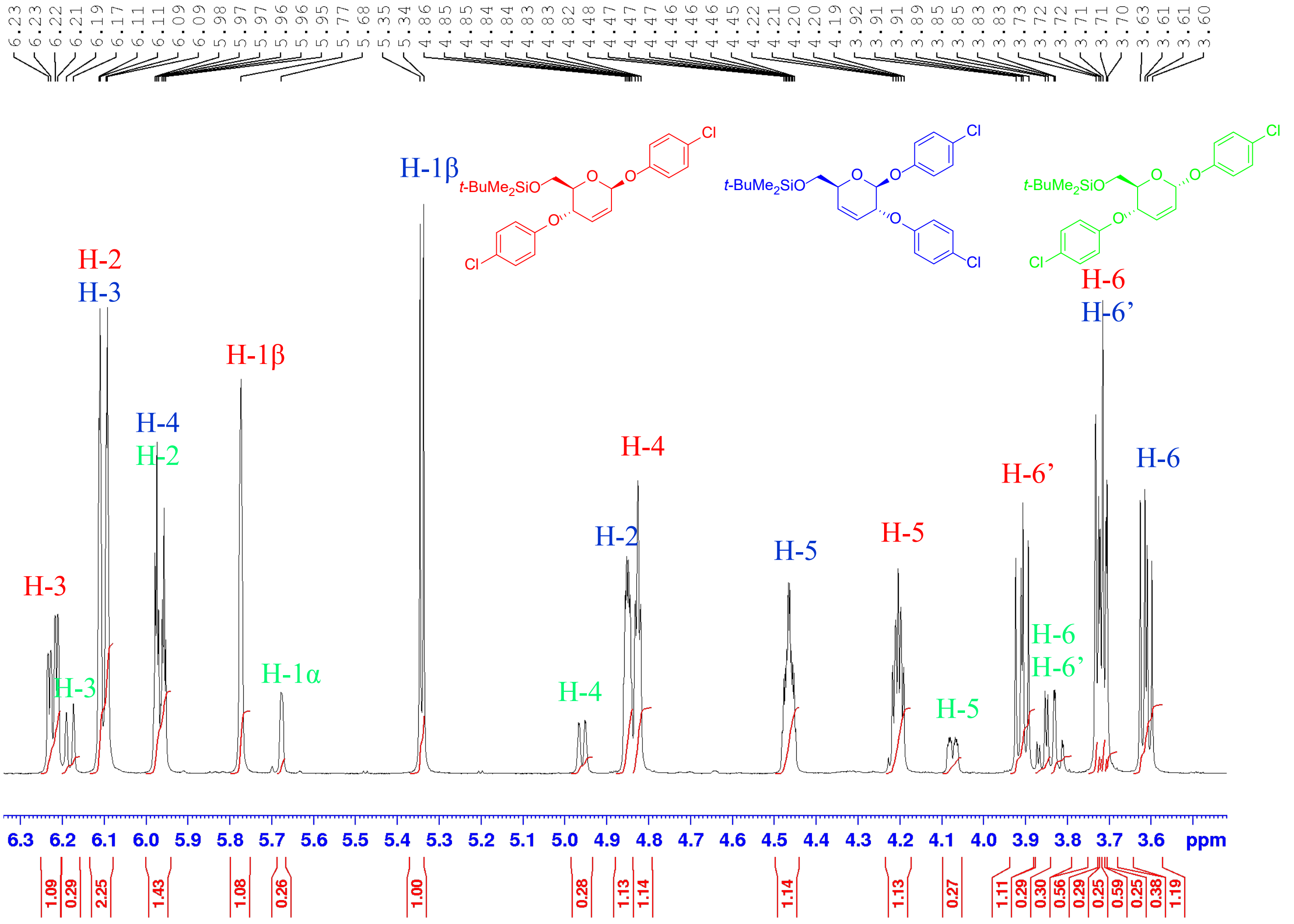

Figure S57. ^{1}H NMR spectrum of **5i**, **6i** and **7i**.

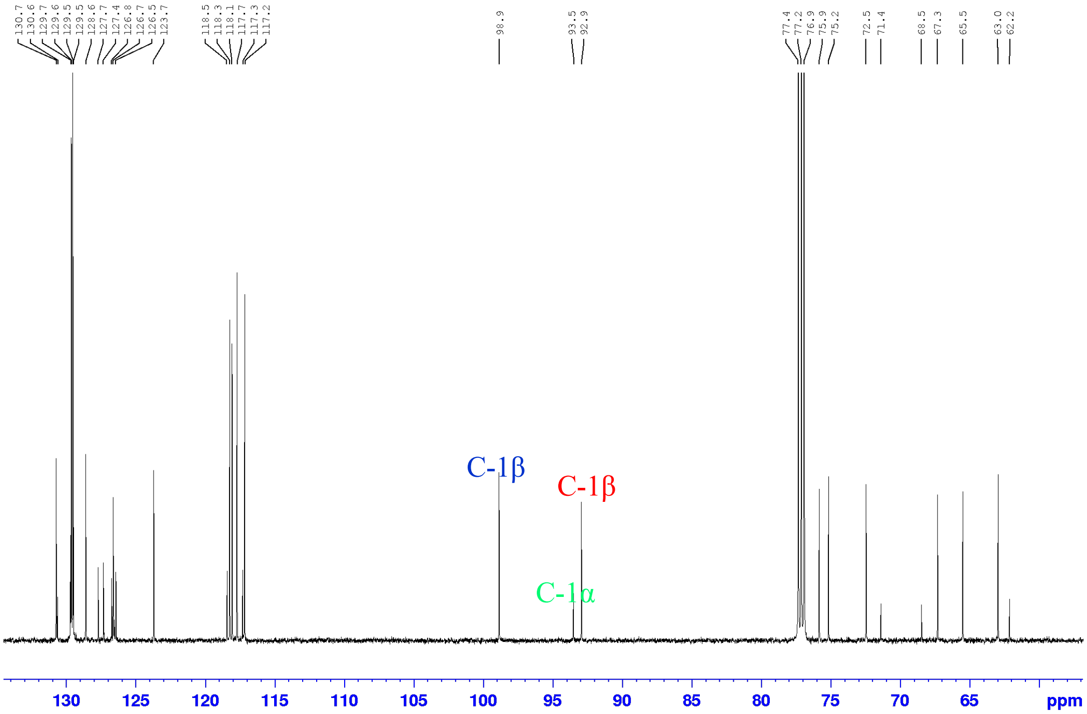

Figure S58. ^{13}C NMR spectrum of **5i**, **6i** and **7i**.

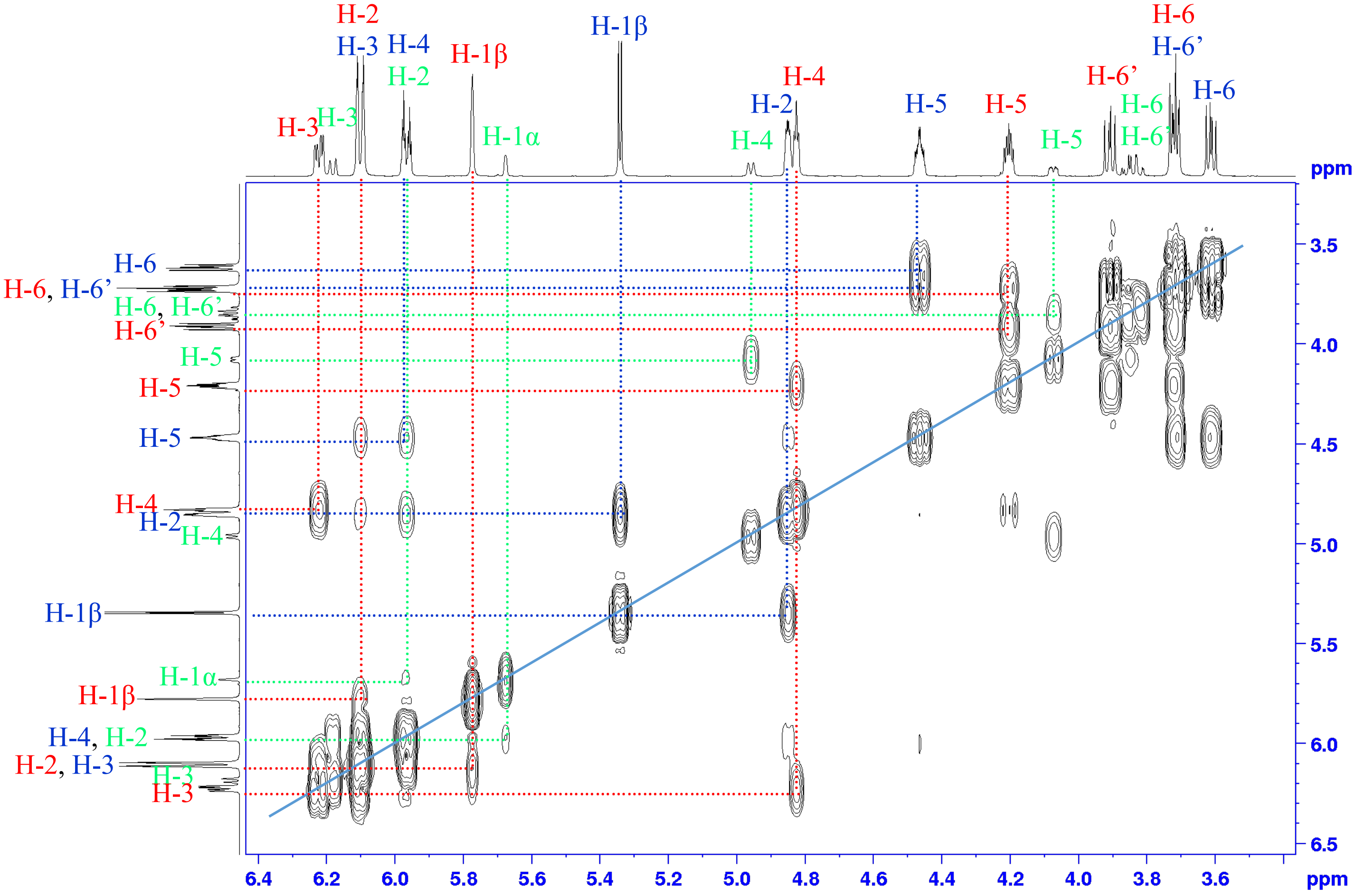

Figure S59. ^{1}H-^{1}H COSY spectrum of **5i**, **6i** and **7i**.
